# PIN1 protects auditory hair cells from senescence via autophagy

**DOI:** 10.7717/peerj.14267

**Published:** 2022-11-01

**Authors:** Zhe Lv, Yanzhuo Zhang, Huan Cao, Qingjuan Liu, Xiaojuan Feng, Huan Yin, BaoShan Wang

**Affiliations:** 1 Department of Otorhinolaryngology, The Second Hospital of Hebei Medical University, Shijiazhuang, Hebei, China; 2 Department of Otorhinolaryngology, Hebei Eye Hospital, Xingtai, Hebei, China; 3 Department of Pathology, Hebei Key Laboratory of Nephrology, Center of Metabolic Diseases and Cancer Research, Hebei Medical University, Shijiazhuang, Hebei, China

**Keywords:** ARHL, HEI-OC1 cells, PIN1, Autophagy, Senescence

## Abstract

**Background:**

Age-related hearing loss is an increasing sensorineural hearing loss. But the pathogenesis of ARHL has not been clarified. Herein, we studied the role and significance of PIN1 in regulating autophagy activity in senescence HEI-OC1cells and HCs.

**Methods and Results:**

C57BL/6 mice and HEI-OC1 cells were contained in our research. Transfection of plasmids and juglone were used to upregulate or inhibit the PIN 1 expression. Immunofluorescence and Western blot were used to detect the expression of PIN1, LC3, p62, p21 and p16 protein levels in the hair cells of C57BL/6 mice cochleae and HEI-OC1 cells. Senescence-associated *β*-galactosidase (SA-*β*-gal) staining was used to investigate the senescent level.The results of this study showed that the level of autophagy increased in the senescent auditory hair cells. When inhibited the autophagy level with 3-MA, the senescent HEI-OC1 cells were alleviated. The autophagy activity in senescent HEI-OC1 cells also could be reduced by overexpressing PIN1 protein. On the contrary, inhibiting PIN1 could increase the autophagy level of senescent cells and cochlear hair cells.

**Conclusion:**

PIN1 might regulate autophagy activity to induce the senescent of HEI-OC1cells and HCs, which will provide a theoretical support for the prevention and treatment of age-related hearing loss.

## Introduction

Age-related hearing loss (ARHL) refers to the symmetrical and slowly progressive sensorineural hearing loss due to the aging of the auditory organs. For the elderly, ARHL seriously affect the quality of life. However, the exact pathogenesis of ARHL is still unclear.

Autophagy is important for cell homeostasis and adaptation to stress, and is an effective way to eliminate harmful and toxic substances for cells (Mizushima et al., 2001). At present, accumulating evidence have indicated that autophagy and senescence are significantly interrelated, and stimulating autophagy could promote longevity (Leidal, Levine & Debnath, 2018). Although the activation of autophagy has been shown to be beneficial in many pathological and physiological conditions, excessive activation could cause cell death. Recent research has found that autophagy was essential for HCs development (Fujimoto et al., 2017). In addition, autophagy participated in the prevention of neomycin-, noise-, and cisplatin-induced auditory damage (Fang & Xiao, 2014; He et al., 2017; Yang et al., 2018; Yuan et al., 2015). In the D-gal induced aging animal model, the autophagy’s level in cochlea obviously increased in the 12th month, however, the level of autophagy significantly decreased in the cochlea in the 24th month of D-gal treatment, and the senescent HEI-OC1 cells autophagy activated that induced by D-gal, accompanying with both reduced dead and apoptotic cells (He et al., 2021). By contrast, Peineau et al. (2021) reported that compared with young mice, LC3B-II was reduced in the cochlear of aged mice. Above all, the precise role of autophagy in ARHL and the underlying mechanism still needs to be investigated.

Accumulating evidence have suggested that prolyl-isomerase (PIN1), a novel signal regulator of post-phosphorylation, could influence cell death via regulating autophagy. In a study of liver cancer, there was negative correlationship between the level of autophagy and the expression of PIN1 (So & Oh, 2015). Our previous research has shown that PIN1 regulated cell senescence through PI3K/Akt/mTOR pathway, treatment with juglone led to C57BL/6 mice hearing loss (Zhang et al., 2021). Since the mechanistic target of rapamycin (mTOR) is known to be a master regulator of autophagy and as a sequel to the previous experiment, we further explored the changes of autophagy during aging, and investigated whether PIN1 played the important regulatory role in autophagy.

Therefore, this study investigated the correlation between PIN1 expression and autophagy in the aging of auditory HCs and HEI-OC1 cells *in vivo* and *in vitro*. Interestingly, the results showed that PIN1 deficiency could induce autophagy activation of auditory HCS and HEI-OC1 cells, thus mediate cell aging.

## Material and Methods

### In vivo

1. Animals and treatment

The 20–30 g male C57BL/6 mice were purchased from the Charles River (Beijing China). All the animals were approved by the Institutional Animal Care and Use Committee of The Second Hospital of Hebei Medical University (approval ID:2019-AE001) and were fed in standard environment that with regular light/dark cycles and free access to water and food diet. Then the mice were randomly divided into young aged group (two-month mice) and old aged group (12-month mice). Additionally, the mice of young aged group (two-month mice) were assigned as control group, juglone group (intraperitoneal injection 1 mg/kg three times a week for four week) and DMSO group again. At the different time, the cochleae were obtained and fixed in 4% PFA overnight at 4 °C after ABR recordings. The labyrinth, stria vascularis, spiral ligament under dissecting microscope were cut according to the previous method (Zhang et al., 2021), and the corresponding measures (immunofluorescence staining and Senescence-associated *β*-Galactosidase Staining) were carried out.

Mice which loss the ability of ambulate or wound healing were euthanized. Necessary measures were used to minimize the animals suffering during the experiment.

2. Immunofluorescence staining

The micro-dissected Corti were washed with PBS three times, and then treated with Triton X-100 (0.3%) for 20 min at room temperature. After washing with PBS three times, goat serum was used to block nonspecific antibody binding. Then the organ of Corti sections were incubated with primary antibodies against p62 (dilute in 1:200; Proteintech, 18420-1-AP, USA) or anti-light chain 3 (LC3, dilute in 1:400; Proteintech, 12135-1-AP, USA) overnight at 4 °C. At the second day, corti sections were incubated with phalloidin and secondary fluorescent antibody kept away from light at 37 °C for 2 h after washing three times with PBS, and then stained with DAPI at room temperature for 10 min. Finally, the positive signals were observed by Olympus microscope (Olympus, Tokyo, Japan). The Image Pro-Plus 5.0 software (Media Cybernetics, Silver Spring, MD, USA) was used to quantified the expression level of stained proteins by digital image analysis, according to the integrated optical density (IOD) of the positive region.

### In vitro

1. Cells culture and groups

The auditory cell line HEI-OC1 was developed at the House Ear Institute (Los Angeles, CA, USA) and Provided by Biofeng (Shanghai, China), which was cultured in high glucose dulbecco’s modified eagle medium (DMEM) with 10% fetal bovine serum (FBS) under the conditions of 33 °C and 10% CO_2_.

2. Transfection of plasmids

HEI-OC1 cells with 70–90% fusion, cultured in 6-well plates, were incubated with mixture of Lipofectamine 3000 and diluted DNA reagent (1:1) at room temperature for 15 min. Then the DNA-lipid complex was added to the cell supernatant and incubated at 33 °C for 48 h.

3. Reagent treatment

We seeded the cells in six-well plates for each experiment for 24 h and then were stimulated by Juglone (a PIN1 inhibitor) (0, 1, 5, 10 µM, STBH9858, Sigma-Aldrich, USA) for 45 min.

3. Senescence-associated *β*-galactosidase staining

*β*-galactosidase staining kit (Beyotime, Shanghai, China) was used to detect cell senescence. Firstly, the cells were washed with PBS, and then fixed with *β*-galactosidase staining fixative before the staining. After washing for 3 times, one mL staining solution was added to each well. The wells were sealed with sealing film and incubated overnight at 37 °C without carbon dioxide.

4. Western blot

Cultured cells were lysed with radioimmunoprecipitation assay buffer (BestBio, Shanghai, China) containing 1% protease inhibitor cocktail (BestBio, Shanghai, China) for 40 min, and then centrifuged at 4 °C for 20 min. The supernatant was collected. Protein samples (30 µg) were electrophoresed on the sodium dodecy1 sulfate-polyacrylamide gel. After transfer to a polyvinylidene fluoride membrane, the blocking procedure was performed with 5% nonfat milk, and the membranes were incubated with primary antibodies of anti-p62 (dilution, 1:1000; #18420-1-AP; Proteintech, Rosemont, IL, USA), anti-LC3 (dilution, 1:1000; 12135-1-AP; Proteintech, Rosemont, IL, USA) and anti- *β*-actin (dilution, 1:1000; 20536-1-AP; Proteintech, Rosemont, IL, USA) overnight at 4 °C. After washing the membranes, horseradish peroxidase-conjugated secondary antibody was applied at room temperature for 60 min. The enhanced chemiluminescence (ECL) was used to identify the protein bands. In the end, the internal reference bands and target protein bands were visualized and calculated using the Gel-Pro analyzer software.

### Statistical analysis

We analyzed the data with SPSS 21.0. The results were expressed as mean ± standard error of mean and were analyzed by One-way ANOVA. *P* < 0.05 was considered as the criterion for statistical significance.

## Results

1. The autophagy was activated in HCs of aging C57BL/6 mice

**Figure 1:**
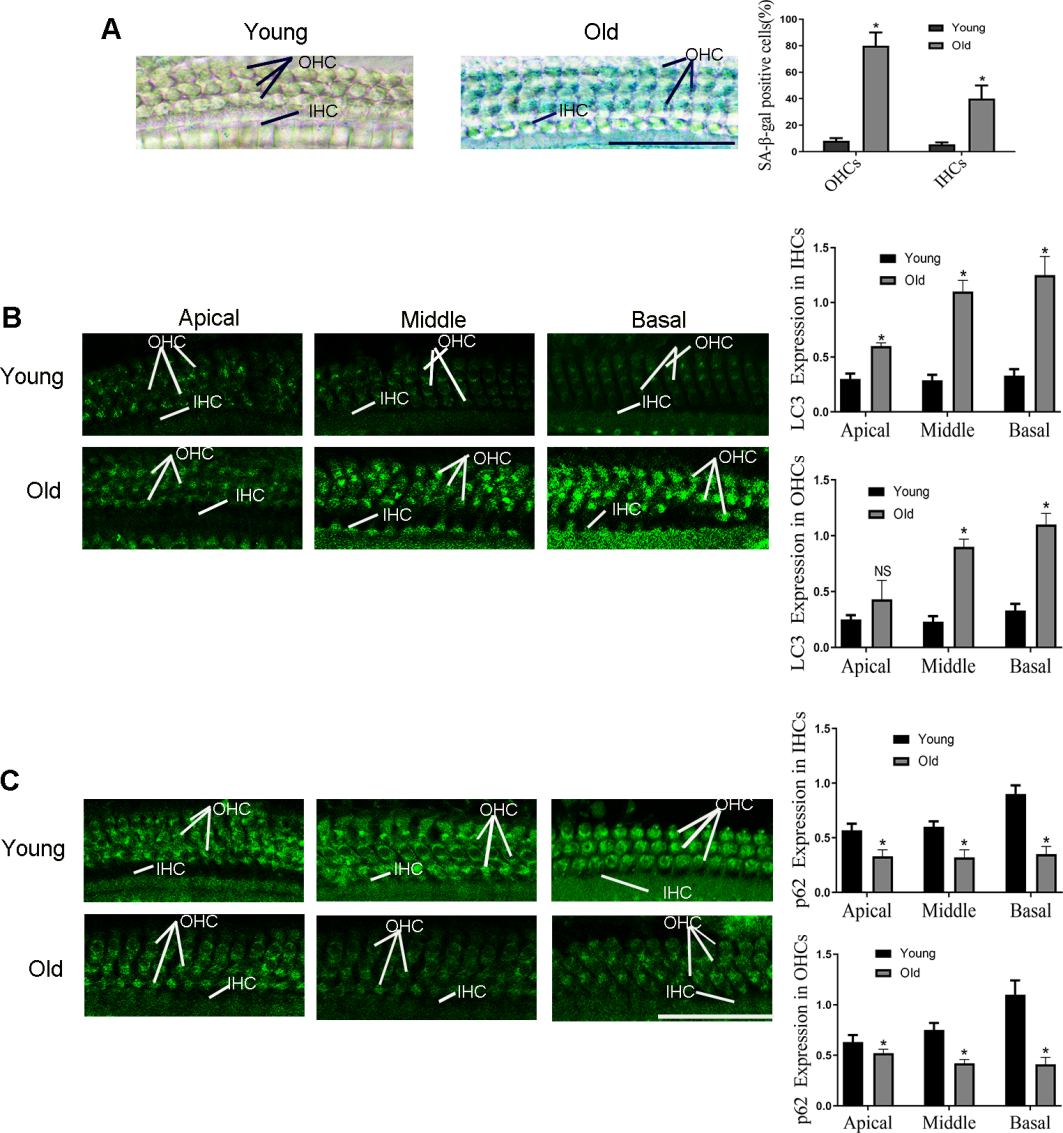
The changes of HCs autophagy levels in C57BL/6 mice at different months of age. Typical picture of senescence-associated *β*-galactosidase (SA- *β*-gal) staining for HCs. Scale bar = 50 µm. (B) Representative immunohistochemical of LC3 for HCs. Scale bar = 50 µm. (C) Representative immunohistochemical of p62 for HCs. One-way ANOVA test and homogeneity of variance test was performed to evaluate the statistical difference. Scale bar = 50 µm. (Young group: *n* = 6, Old group: *n* = 6).

As shown in our previous study, a severe hearing loss was found in the 12-month-old C57BL/6 mice (Zhang et al., 2021). In addition, the significantly up-regulated senescence level was observed in outer hair cells (OHCs) and inner hair cells (IHCs) of old-aged mice according to the Fig. 1A, and the most obvious change is in basal turn. Then the expression level of LC3 and p62 were examined in the following experiments to detect autophagy activity. As illustrated in Fig. 1B, compared with the 2-month-old mice (named as young-aged mice), LC3 protein was elevated in the OHCs and IHCs of the old-aged mice. By contrast, compared with the young mice, expression of p62 was lower in the OHCs and IHCs of the old-aged mice (Fig. 1C), especially in the middle and basal turn.

2. Inhibition of autophagy reduced H_2_O_2_-induced aging.

As proved in our previous study, H_2_O_2_ induced senescence of HEI-OC1 cell through activating the PI3K/Akt pathway (Zhang et al., 2021). To further identify the mechanistic contributions of autophagy to H_2_O_2_-induced senescence, HEI-OC1 cells was treated with H_2_O_2_ (1 mM for 2 h, 4 h, 8 h) and the expression of LC3-II, p62 (which were named as autophagy associated protein), and p21, p16 (which named as senescent associated protein), was detected. Compared with control group, p16, p21 and LC3-II was significantly elevated, whereas the expression of p62 was significantly decreased in cells incubated with H_2_O_2_ in a time-dependent manner.

**Figure 2:**
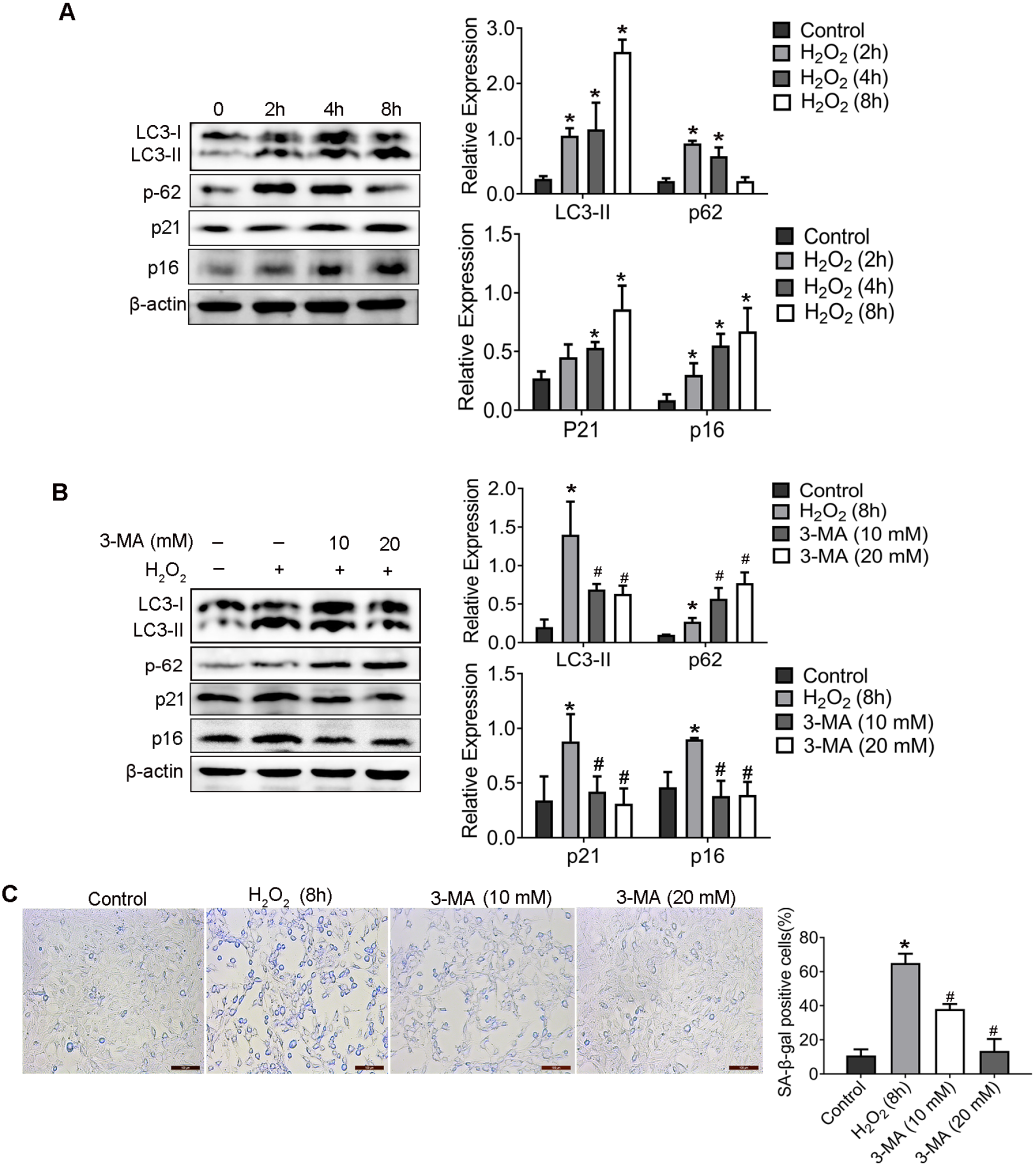
H_2_O_2_ enhanced HEI-OC1 cell autophagy and 3-MA could reduce the H_2_O_2_-induced aging. (A) The HEI-OC1 cells were treated with H_2_O_2_ (1mM for 2 h, 4 h, 8h) and examined LC3-II, p62, p21 and p16 level (^*^P < 0.05 *vs.* control group). B. Western blot for expression of LC3-II, p62, p21 and p16 level when we pretreat HEI-OC1 cells used 3-MA (^*^P < 0.05 *vs.* control group, ^#^*P* < 0.05 *vs.* H_2_O_2_ group). C. SA- *β*-gal staining for HEI-OC1 cells. Scale bar = 100 µm. One-way ANOVA test and homogeneity of variance test was performed to evaluate the statistical difference. (^*^P < 0.05 *vs.* control group, ^#^*P* < 0.05 *vs.* H_2_O_2_ group).

Therefore, in order to explore the effect of autophagy on HEI-OC1 cells senescence, 3-Methyladenine (3-MA), an effective autophagy inhibitor, was used to inhibit the autophagy. WB result of LC3-II and p62 confirmed the inhibition effect of autophagy of 3-MA (Fig. 2B). Importantly, compared with the H_2_O_2_ group, the levels of p16 and p21 protein decreased in 3-MA pretreatment group. Additionally, as shown in Fig. 2C, 3-MA pretreatment also significantly reduced the percentage of senescence-associated *β*-galactosidase (SA- *β*-gal) positive cells induced by H_2_O_2_.

3. PIN1 was involved in the autophagy activation of aged HEI-OC1 cells

**Figure 3:**
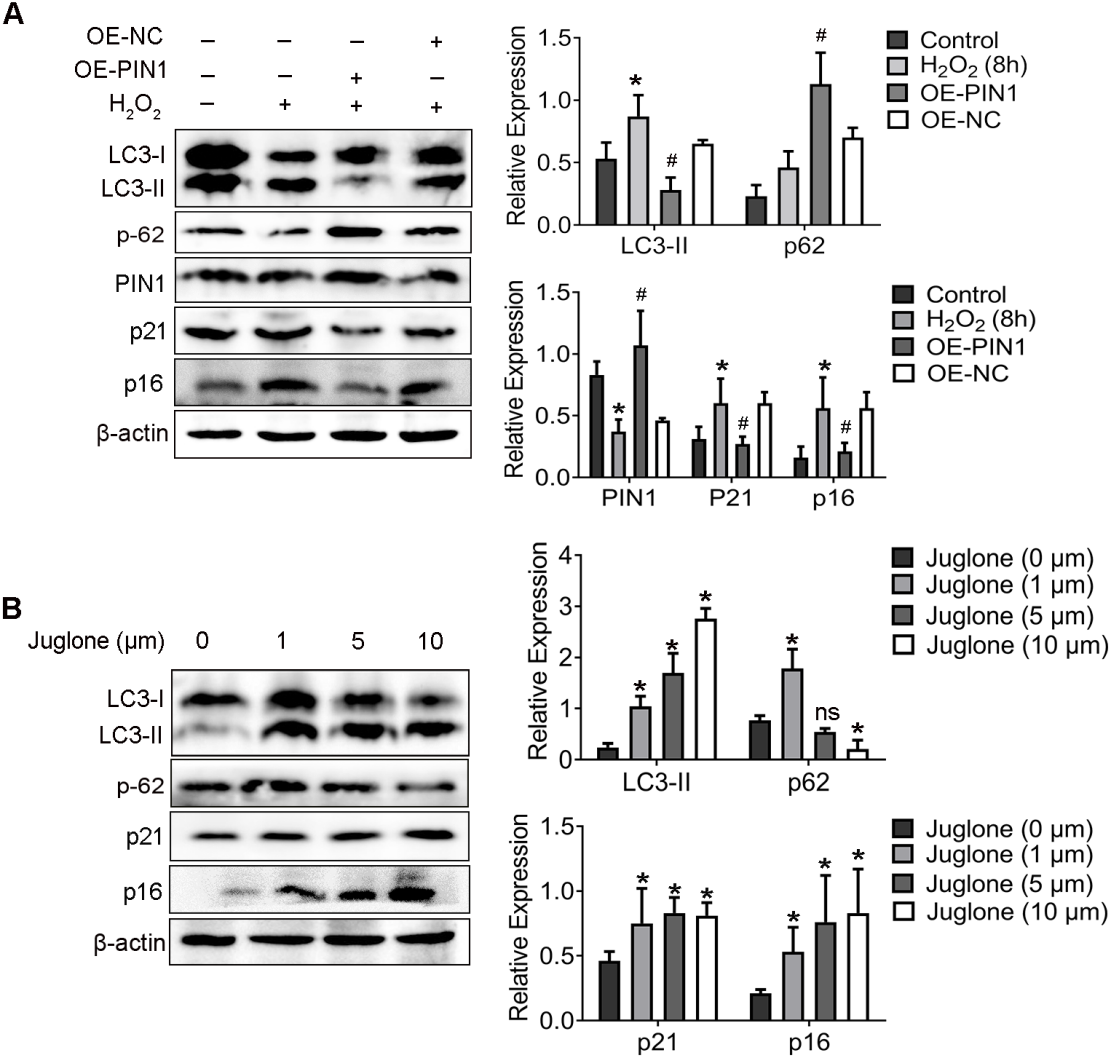
PIN1 mediates HEI-OC1 cells senescence by affecting autophagic activity of cells. (A) Western blot for expression of LC3-II, p62, PIN1, p21 and p16 in HEI-OC1 cells were pretreated with the HA-PIN1 plasmid. (^*^P < 0.05 *vs.* control group, ^#^*P* < 0.05 *vs.* NC group, OE: overexpression-PIN1). (B) Western blot for expression of LC3-II, p62, p21 and p16 in HEI-OC1 cells after pretreatment with juglone. One-way ANOVA test and homogeneity of variance test was performed to evaluate the statistical difference. (^*^P < 0.05 *vs.* control group).

As shown in our previous study, down-regulation of PIN1 is associated with senescence of HEI-OC1 cells induced by H_2_O_2_ (Zhang et al., 2021). Corroborating the results reported in that publication, we found here that H_2_O_2_ also attenuated the expression of PIN1 protein (Fig. 3A). To further explore whether the downregulation of PIN1 was contributed to senescence of HEI-OC1 cells by regulating the autophagy of HEI-OC1 cells, we employed juglone (1 mM for 8 h) and expression vector (OE-PIN1) to inhibit or upregulate the expression of PIN1. Interestingly, compared with NC group, the expression of LC3-II, p21 and p16 was significantly decreased, which the p62 expression increased in the OE-PIN1 group (Fig. 3A). On the contrary, treatment of juglone obviously increased the expression of LC3-II, p21 and p16, decreased the p62 protein expression of HEI-OC1 cells (Fig. 3B). Together, these results suggested that PIN1 might be involve in the senescence of HEI-OC1 cells by mediating autophagy activity.

4. PIN1 inhibition induced autophagy of hair cells and exacerbated hearing loss in C57BL/6 mice.

**Figure 4:**
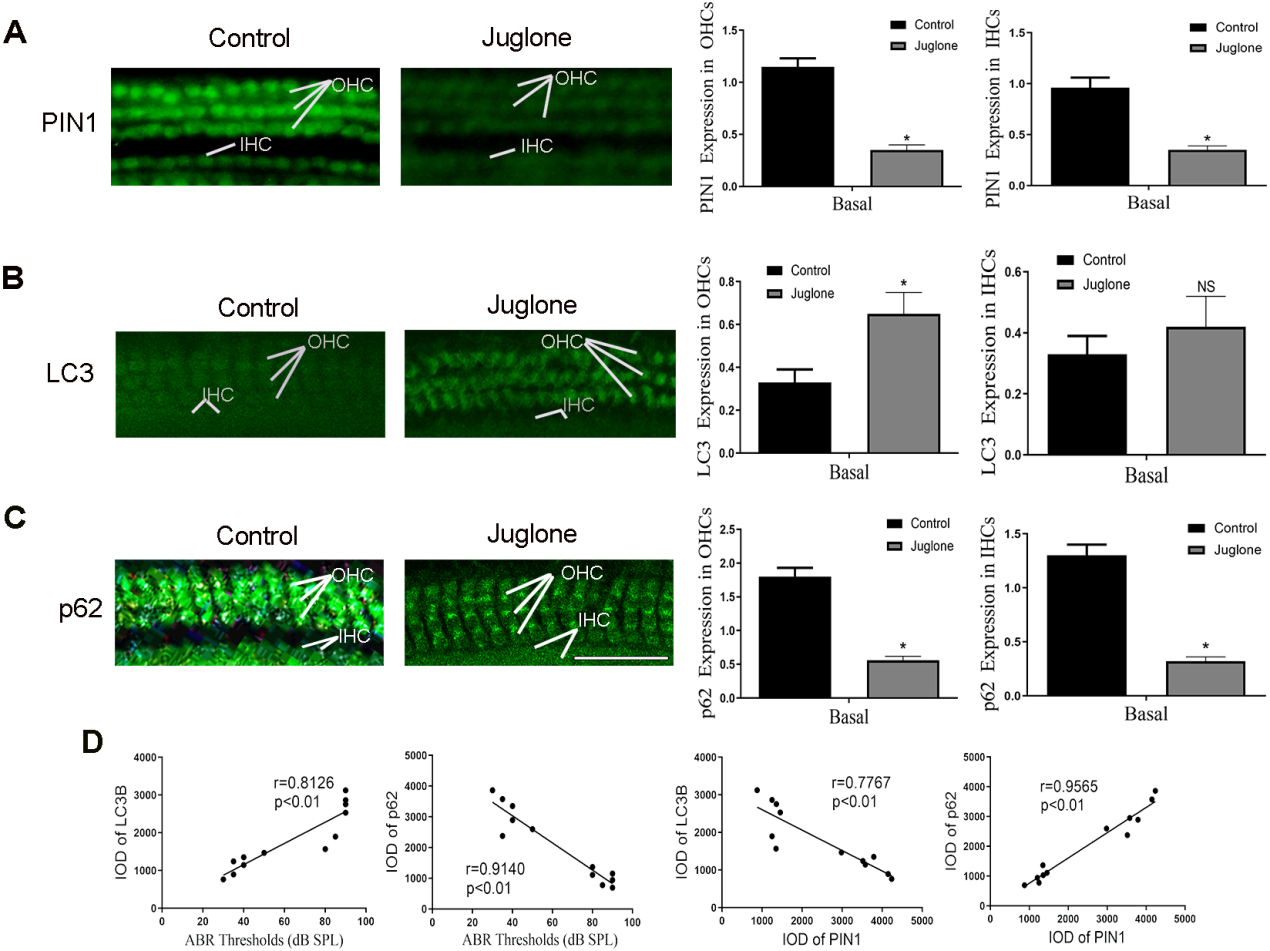
Juglone treatment induce autophagy in C57BL/6 mouse hair cells. (A) PIN1 levels in the basal turn hair cells. (B) Immunohistochemical of LC3 for HCs when treated C57BL/6 mice by juglone. Scale bar = 50 µm. (C) Immunohistochemical of p62 for HCs when treated C57BL/6 mice by juglone. Scale bar = 50 µm. (control group: *n* = 6; juglone group: *n* = 6). (D) Linear analysis. LC3 level was positively correlated with hearing threshold, the expression of p62 was negatively correlated with hearing threshold. After treated with juglone, PIN1 expression in cochlea was correlated with LC3 negatively, positively correlated with p62. One-way ANOVA test, homogeneity of variance test and Pearson’s method were performed.

Next, C57BL/6 mice treated with juglone was used to investigate the potential role of PIN1 in the development of hair cell autophagy in mice. The effectiveness of juglone was confirmed by immunofluorescent staining of PIN1 expression, the basal turn is the most obvious (Fig. 4A). More notably, immunofluorescent staining also indicated that LC3 was increased in OHCs of juglone-treated mice in the basal turn, in contrast, p62 was decreased, which indicating increased autophagic activity (Figs. 4B and 4C). Linear analysis indicated that the protein level of LC3 was positively correlated with hearing threshold, and p62 was negatively correlated with it (the results of hearing threshold were shown in Zhang et al., 2021). Simultaneously, PIN1 expression in cochlea of juglone-treated mice was negatively correlated with LC3-II and positively correlated with p62 (Fig. 4D).

## Discussion

ARHL is a risk factor for cognitive decline and dementia, and it is also a common sensory impairment in the elderly adults. The risk of dementia may be reduced by 9% if midlife hearing loss is eliminated (Thomson et al., 2017; Uchida et al., 2019). But so far, the understanding of the mechanisms in ARHL is limited (Bowl & Dawson, 2019). Recently, the main pathological changes of ARHL are mainly the loss of cochlear hair cells (HCs), especially in the outer hair cells (OHCs) of the basal layer of the cochlea. Therefore, finding ways to protect cochlear HCs from aging might be a very important issue (Bowl & Dawson, 2015; Bao & Ohlemiller, 2010).

In general, basal levels of autophagy are present in almost all cells, which can not only regulate cell survival and death, but also plays an important role in pathological and physiological conditions. However, autophagy is more active in the environment of hypoxia, nutrient deficiency or pathogen invasion. Aging could lead to the molecules accumulation, organelles damage, the imbalance of oxidation and antioxidant levels in the body, and the increase in the generation rate of free radicals, in which autophagy could not only remove damaged proteins and non-functional organelles to promote cell survival, but also inhibit the increase in ROS levels during cellular senescence (Korolchuk et al., 2017; Fujimoto & Yamasoba, 2014; Bellot et al., 2009; Shen & Codogno, 2012; Berk et al., 2013).

However, there were divergent opinions on the effects of autophagy in the cochleae. In a study of cisplatin-induced deafness, rapamycin (autophagy inducer) has been found to promote cochlear cell from apoptosis, while 3-MA, the autophagy inhibitor, could reverse its promoting effect on the apoptosis. Additionally, it was found that the apoptosis and autophagy were promoted by rapamycin (autophagy inducer) in the cochlea, and the promotive effect on the apoptosis of autophagy can be overturned by 3-MA (autophagy inhibitor) (Xu, Yan & Cheng, 2020). Youn et al. (2015) found that LC3-II was significantly increased in HEI-OC1 cells induced by cisplatin for 48 h. More importantly, the autophagy was up-regulated in the early stage to protect cell activity, while later autophagy was increased to induce cell apoptosis (Youn et al., 2015). On the contrary, other studies have reported that in the neomycin or gentamicin induced injury of HCs and HEI-OC1 cells, rapamycin could inhibit the apoptosis of cells (He et al., 2017). In addition, autophagosomes were increased in H_2_O_2_-induced senescent HEI-OC1 cells, but mitophagy function declined in 32-week-old C57/BL6 mice (Kim et al., 2021). The autophagy level in the cochlea decreased significantly after the successful modeling of simulated aging rats, while in the D-gal induced aging model, the expression level of LC3B first increased and then decreased with the increase of D-gal concentration (He et al., 2021). In our study, not only in H_2_O_2_-induced HEI-OC1 cells but also in HCs cell of middle- or old-aged mice, especially in H_2_O_2_-induced HEI-OC1 cells, the autophagy level all increased following with increased expression of p16 and p21, two of the senescent associated proteins. Notably, administration of 3-MA, an effective autophagy inhibitor, significantly reduced the percentage of senescence-associated *β*-galactosidase (SA- *β*-gal) positive cells induced by H_2_O_2_ and the expression of p16 and p21, while suggesting that H_2_O_2_ induced the senescence in aging partly by regulating autophagy.

PIN1 is a unique enzyme that controls the functions of its these substrates through interacting with the motif containing phospho-Ser/Thr-Pro substrates and enhancing cis-trans isomerization of peptide bonds. However, it remains undefined for its role in autophagy induction. In a study on breast cancer, the researchers found that PIN1 could induce the expression of LC-3 and there was a positive correlation between the levels of LC-3 and PIN1 in human breast cancer (Namgoong et al., 2010). Similarly, cadmium (Cd)-induced autophagy was regulated by PIN1 in oral squamous cell carcinoma (OSCC) cell line. High expression of PIN1 in human oral squamous carcinoma cells played an important role in protection of cells against cellular stress (So, Ahn & Oh, 2015). Our precious research has proved that downregulation of PIN1 significantly contributed to the senescence (Zhang et al., 2021). To further explore the effect of PIN1 on the correlation between the senescence and autophagy, we first used PIN1 plasmid to overexpress the PIN1 and observed the change of autophagy and senescence, the results showed that the level of autophagy was significantly inhibited following with the improvement of senescence when PIN1 was overexpressed in HEI-OC1 cells by transfecting PIN1 plasmid and then treated with H_2_O_2_. In contrast, autophagy was activated and the senescence level increased once HEI-OC1 cells was pretreated with juglone (PIN1 inhibitor). Above all, the downregulation of PIN1 was involved in the regulation of autophagy in H_2_O_2_-induced cellular senescence in HEI-OC1 cells. In addition, in order to deeply investigate the effect of PIN1 on age-related hearing loss and possible mechanism, we further used juglone to inhibit the expression of PIN1 in HCs of young mice for *in vivo* experiments. The results showed that compared with the control group, the expression of LC3 was increased and the level of p62 was decreased, in the meanwhile, the ABR threshold in high and low frequencies was increased in juglone treatment group, suggesting that inhibition of PIN1 *in vivo* could induce autophagy of hair cells and exacerbate hearing loss in C57BL/6 mice.

## Conclusions

In conclusion, our results showed that PIN1 might regulate autophagy activity to induce the senescent of HEI-OC1cells and HCs, which will provide a theoretical support for the prevention and treatment of age-related hearing loss.

##  Supplemental Information

10.7717/peerj.14267/supp-1 Supplemental Information 1 Author Checklist

10.7717/peerj.14267/supp-2 Supplemental Information 2 All uncropped blots of Figures 2 and 3

10.7717/peerj.14267/supp-3 Supplemental Information 3 Raw data of Figures 2 and 4 Raw data for ABR thresholds, protein expression, SA- *β*-gal positive cells and IOD

10.7717/peerj.14267/supp-4 Supplemental Information 4 Raw data of Figure 1 Raw data for ABR thresholds, protein expression, SA- *β*-gal positive cells

## Additional Information and Declarations

### Data Availability

The following information was supplied regarding data availability: The raw measurements are available in the Supplemental Files.

## References

[ref-1] Bao J, Ohlemiller KK (2010). Age-related loss of spiral ganglion neurons. Hearing Research.

[ref-2] Bellot G, Garciamedina R, Gounon P, Chiche J, Roux D, Pouysségur J, Mazure NM (2009). Hypoxia-induced autophagy is mediated through hypoxia-inducible factor induction of BNIP3 and BNIP3L via their BH3 domains. The European Journal of Cancer.

[ref-3] Berk M, Dean O, Drexhage H, McNeil JJ, Moylan S, O’Neil A, Davey CG, Sanna L, Maes M (2013). Aspirin: a review of its neurobiological properties and therapeutic potential for mental illness. BMC Medicine.

[ref-4] Bowl MR, Dawson SJ (2015). The mouse as a model for age-related hearing loss—a mini-review. Gerontology.

[ref-5] Bowl MR, Dawson SJ (2019). Age-related hearing loss. Cold Spring Harbor Perspectives in Medicine.

[ref-6] Fang B, Xiao H (2014). Rapamycin alleviates cisplatin-induced ototoxicity *in vivo*. Biochemical and Biophysical Research Communications.

[ref-7] Fujimoto C, Iwasaki S, Urata S, Morishita H, Sakamaki Y, Fujioka M, Kondo K, Mizushima N, Yamasoba T (2017). Autophagy is essential for hearing in mice. Cell Death and Disease.

[ref-8] Fujimoto C, Yamasoba T (2014). Oxidative stresses and mitochondrial dysfunction in age-related hearing loss. Oxidative Medicine and Cellular Longevity.

[ref-9] He Z, Guo L, Shu Y, Fang Q, Zhou H, Liu Y, Liu D, Lu L, Zhang X, Ding X, Liu D, Tang M, Kong W, Sha S, Li H, Gao X, Chai R (2017). Autophagy protects auditory hair cells against neomycin-induced damage. Autophagy.

[ref-10] He ZH, Li M, Fang QJ, Liao FL, Zou SY, Wu X, Sun HY, Zhao XY, Hu YJ, Xu XX, Chen S, Sun Y, Chai RJ, Kong WJ (2021). FOXG1 promotes aging inner ear hair cell survival through activation of the autophagy pathway. Autophagy.

[ref-11] Kim YJ, Choo OS, Lee JS, Jang JH, Woo HG, Choung YH (2021). BCL2 Interacting protein 3-like/NIX-mediated mitophagy plays an important role in the process of age-related hearing loss. Neuroscience.

[ref-12] Korolchuk VI, Miwa S, Carroll B, Von Zglinicki T (2017). Mitochondria in cell senescence: is mitophagy the weakest link?. EBioMedicine.

[ref-13] Leidal AM, Levine B, Debnath J (2018). Autophagy and the cell biology of age-related disease. Nature Cell Biology.

[ref-14] Mizushima N, Yamamoto A, Hatano M, Kobayashi Y, Kabeya Y, Suzuki K, Tokuhisa T, Ohsumi Y, Yoshimori T (2001). Dissection of autophagosome formation using apg5-deficient mouse embryonic stem cells. Journal of Cell Biology.

[ref-15] Namgoong GM, Khanal P, Cho HG, Lim SC, Oh YK, Kang BS, Shim JH, Yoo JC, Choi HS (2010). The prolyl isomerase Pin1 induces LC-3 expression and mediates tamoxifen resistance in breast cancer. The Journal of Biological Chemistry.

[ref-16] Peineau T, Belleudy S, Pietropaolo S, Bouleau Y, Dulon D (2021). Synaptic release potentiation at aging auditory ribbon synapses. Frontiers in Aging Neuroscience.

[ref-17] Shen HM, Codogno P (2012). Autophagy is a survival force via suppression of necrotic cell death. Experimental Cell Research.

[ref-18] So KY, Ahn SG, Oh SH (2015). Autophagy regulated by prolyl isomerase Pin1 and phospho-Ser-GSK3 *αβ* involved in protection of oral squamous cell carcinoma against cadmium toxicity. Biochemical and Biophysical Research Communications.

[ref-19] So KY, Oh SH (2015). Prolyl isomerase Pin1 regulates cadmium-induced autophagy via ubiquitin-mediated post-translational stabilization of phospho-Ser GSK3 *αβ* in human hepatocellular carcinoma cells. Biochemical Pharmacology.

[ref-20] Thomson RS, Auduong P, Miller AT, Gurgel RK (2017). Hearing loss as a risk factor for dementia: a systematic review. Laryngoscope Investigative Otolaryngology.

[ref-21] Uchida Y, Sugiura S, Nishita Y, Saji N, Sone M, Ueda H (2019). Age-related hearing loss and cognitive decline—the potential mechanisms linking the two. Auris Nasus Larynx.

[ref-22] Xu F, Yan W, Cheng Y (2020). Pou4f3 gene mutation promotes autophagy and apoptosis of cochlear hair cells in cisplatin-induced deafness mice. Archives of Biochemistry and Biophysics.

[ref-23] Yang Q, Sun G, Yin H, Li H, Cao Z, Wang J, Zhou M, Wang H, Li J (2018). PINK1 protects auditory hair cells and spiral ganglion neurons from cisplatin-induced ototoxicity via inducing autophagy and inhibiting JNK signaling pathway. Free Radical Biology and Medicine.

[ref-24] Youn CK, Kim J, Park JH, Do NY, Cho SI (2015). Role of autophagy in cisplatin-induced ototoxicity. International Journal of Pediatric Otorhinolaryngology.

[ref-25] Yuan H, Wang X, Hill K, Chen J, Lemasters J, Yang SM, Sha SH (2015). Autophagy attenuates noise-induced hearing loss by reducing oxidative stress. Antioxid Redox Signal.

[ref-26] Zhang Y, Lv Z, Liu Y, Cao H, Yang J, Wang B (2021). PIN1 protects hair cells and auditory HEI-OC1 cells against senescence by inhibiting the PI3K/Akt/mTOR pathway. Oxidative Medicine and Cellular Longevity.

