# Supplementary figures and images for: PIN1 protects auditory hair cells from senescence via autophagy (part 1 of 2)
Source: PeerJ. 2022 Nov 1;10:e14267. doi: 10.7717/peerj.14267 (PMC9635358; doi:10.7717/peerj.14267)

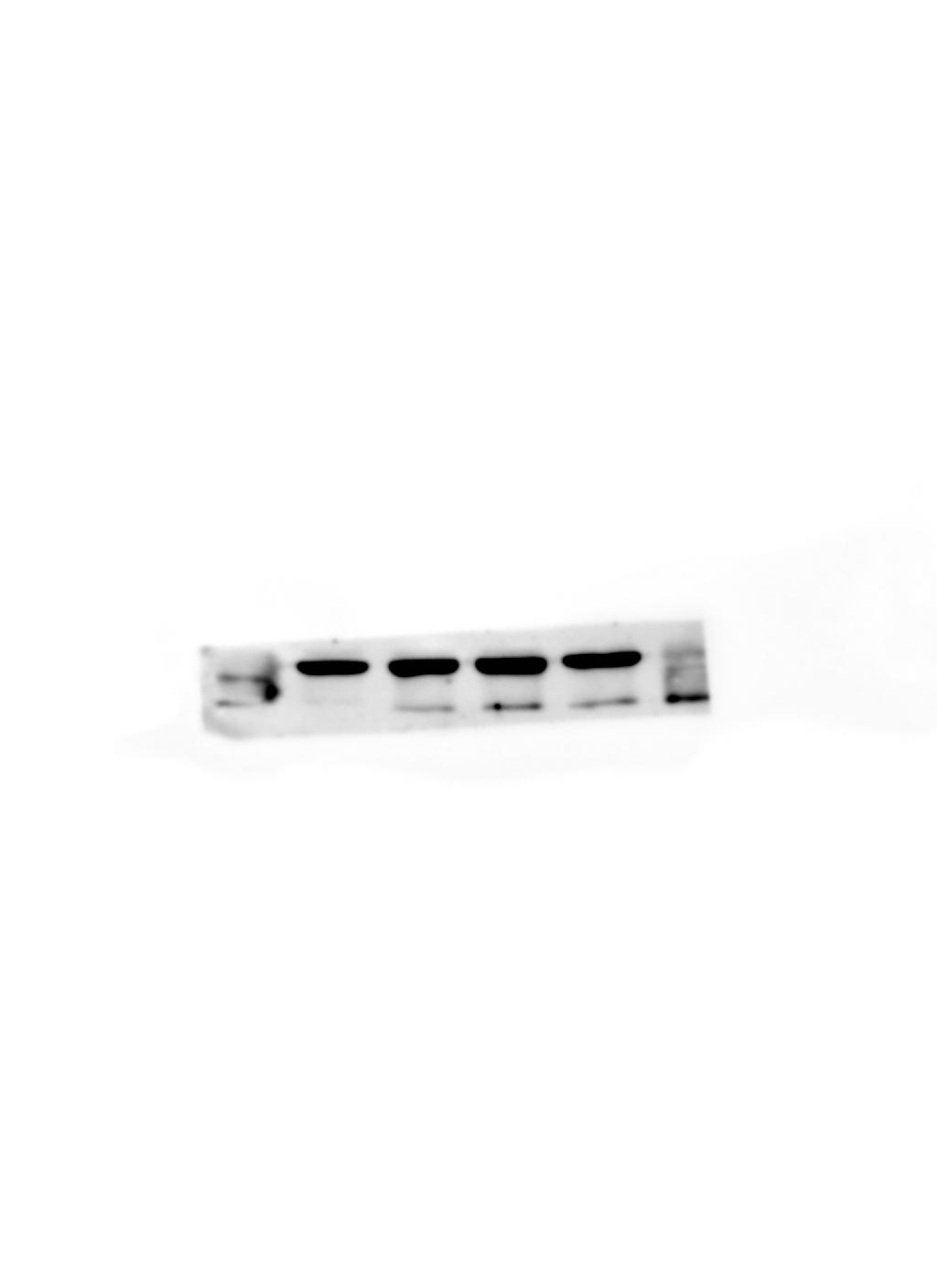

Supplement: Supplemental Information 2 [file peerj-10-14267-s002.zip › uncropped blots/figure2-uncropped blots/A-uncropped blots/actin-1.jpg]

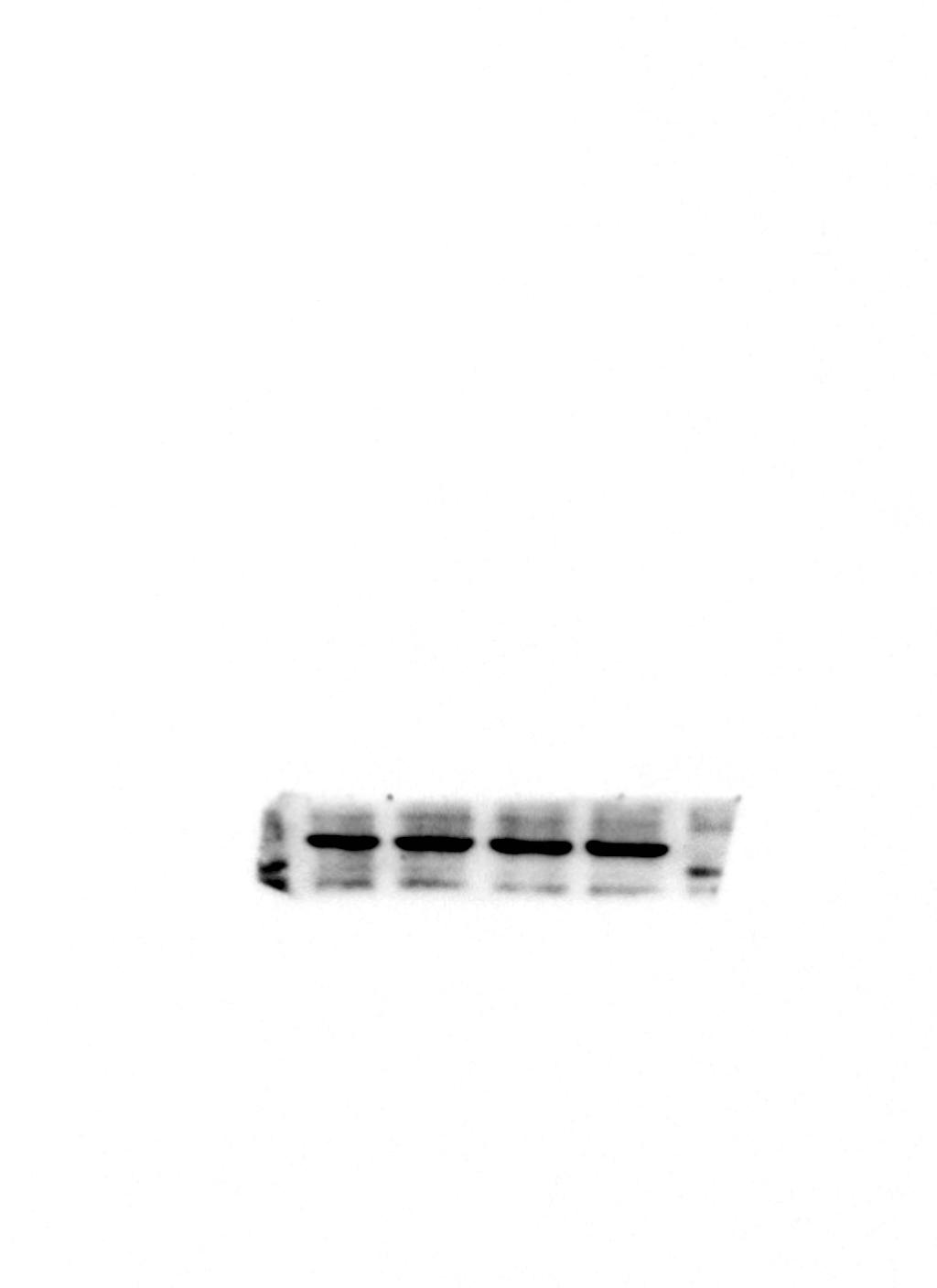

Supplement: Supplemental Information 2 [file peerj-10-14267-s002.zip › uncropped blots/figure2-uncropped blots/A-uncropped blots/actin-2.jpg]

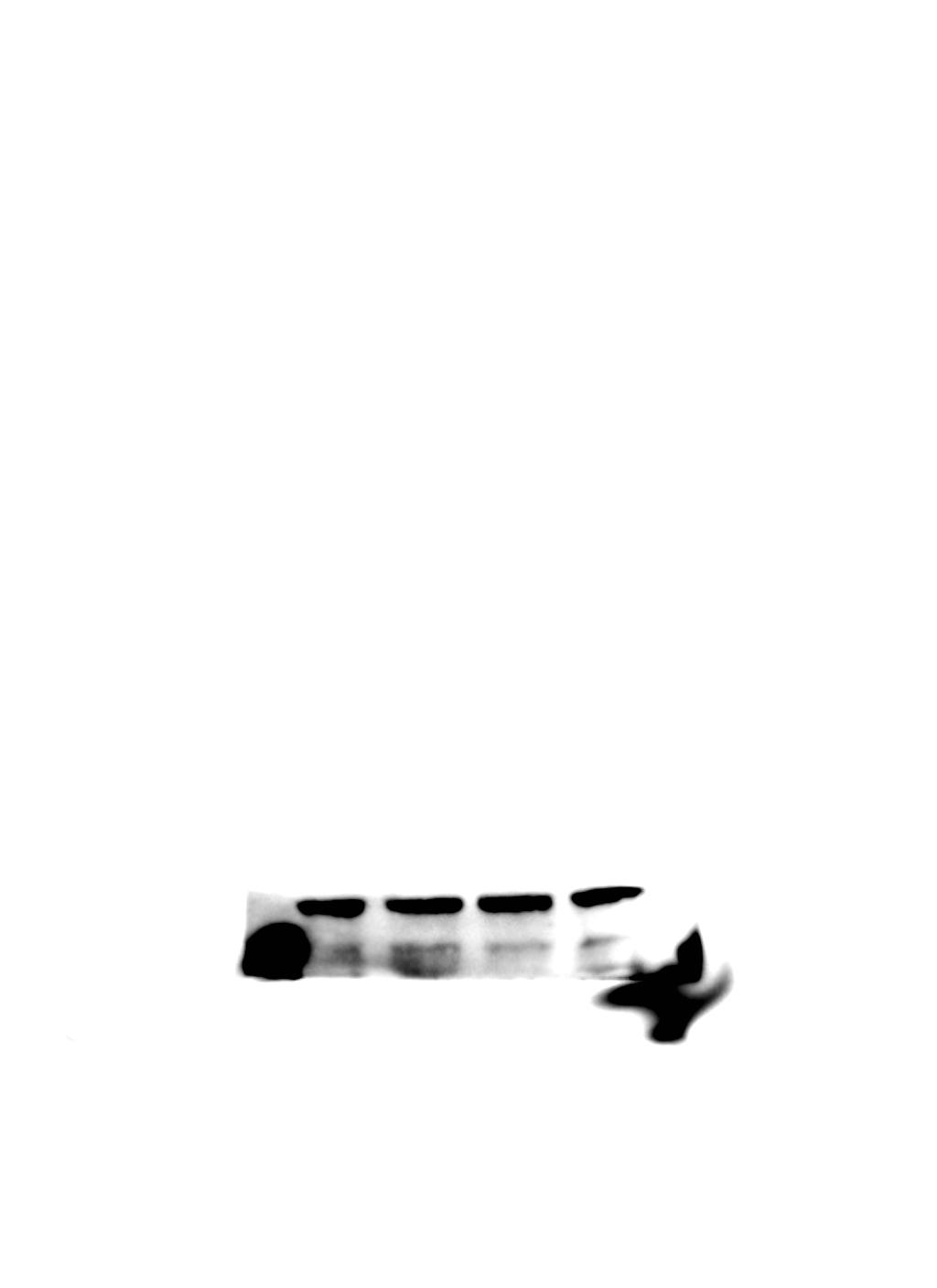

Supplement: Supplemental Information 2 [file peerj-10-14267-s002.zip › uncropped blots/figure2-uncropped blots/A-uncropped blots/actin-3.jpg]

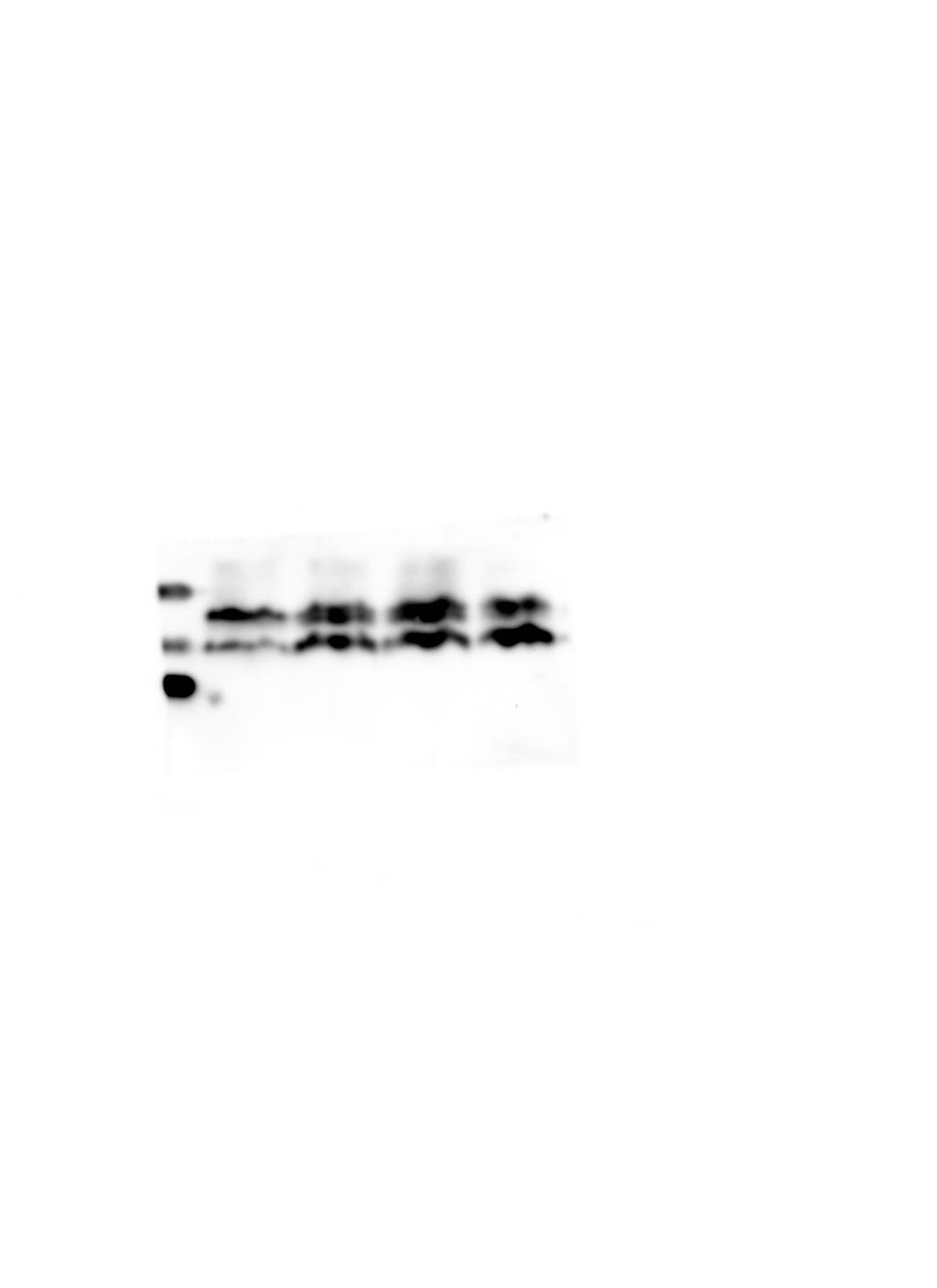

Supplement: Supplemental Information 2 [file peerj-10-14267-s002.zip › uncropped blots/figure2-uncropped blots/A-uncropped blots/LC3-1.jpg]

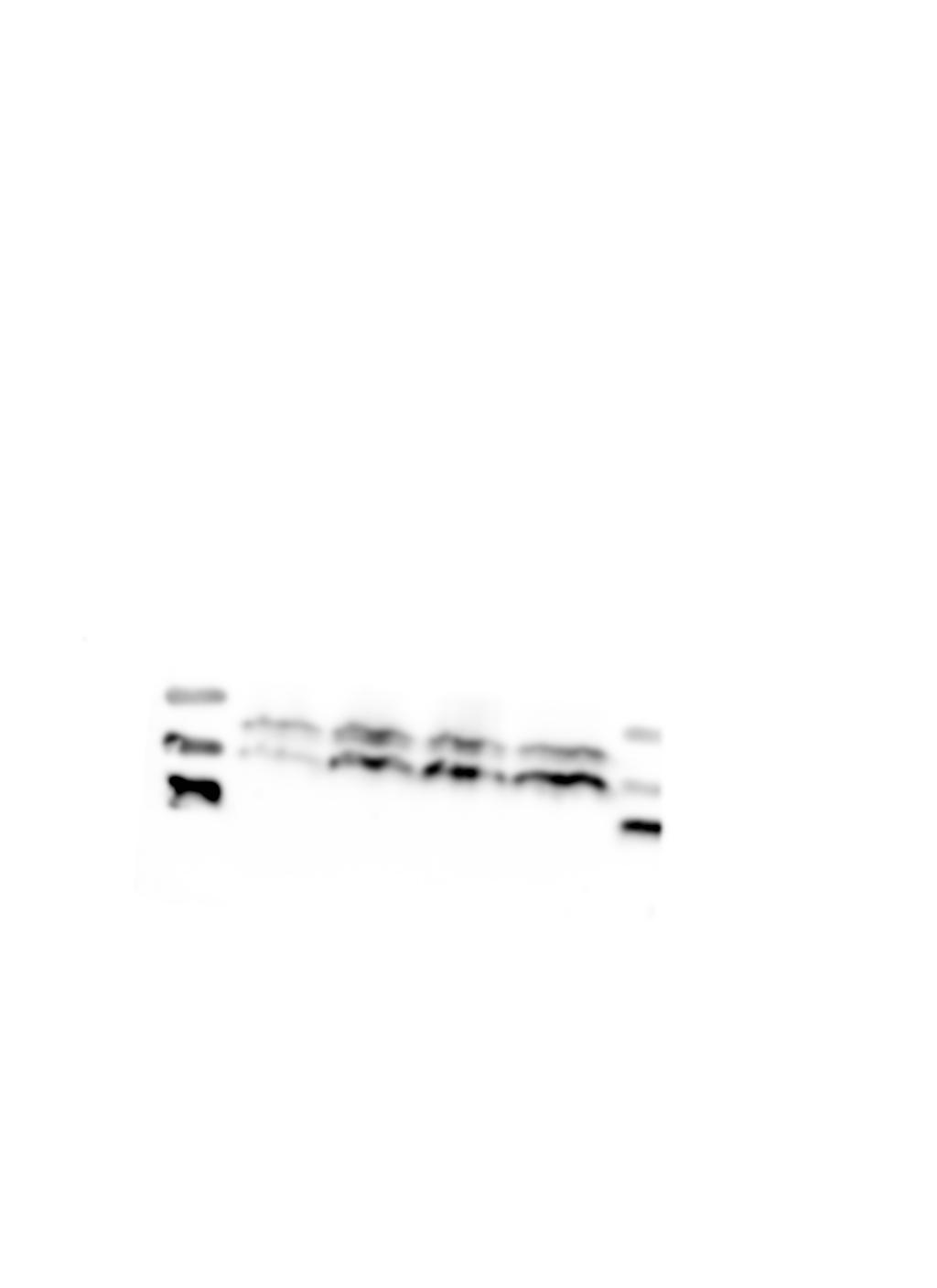

Supplement: Supplemental Information 2 [file peerj-10-14267-s002.zip › uncropped blots/figure2-uncropped blots/A-uncropped blots/LC3-2.jpg]

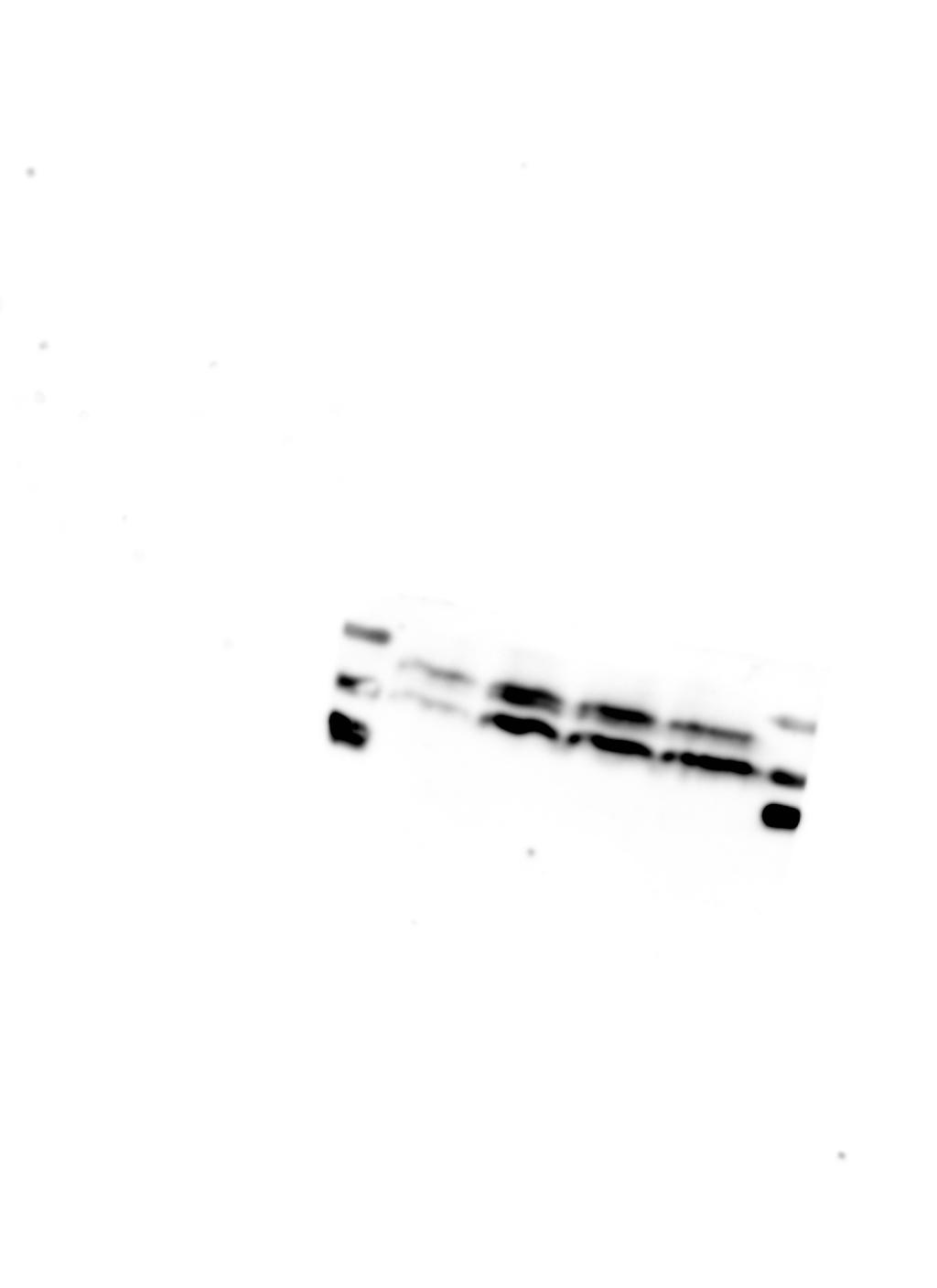

Supplement: Supplemental Information 2 [file peerj-10-14267-s002.zip › uncropped blots/figure2-uncropped blots/A-uncropped blots/LC3-3.jpg]

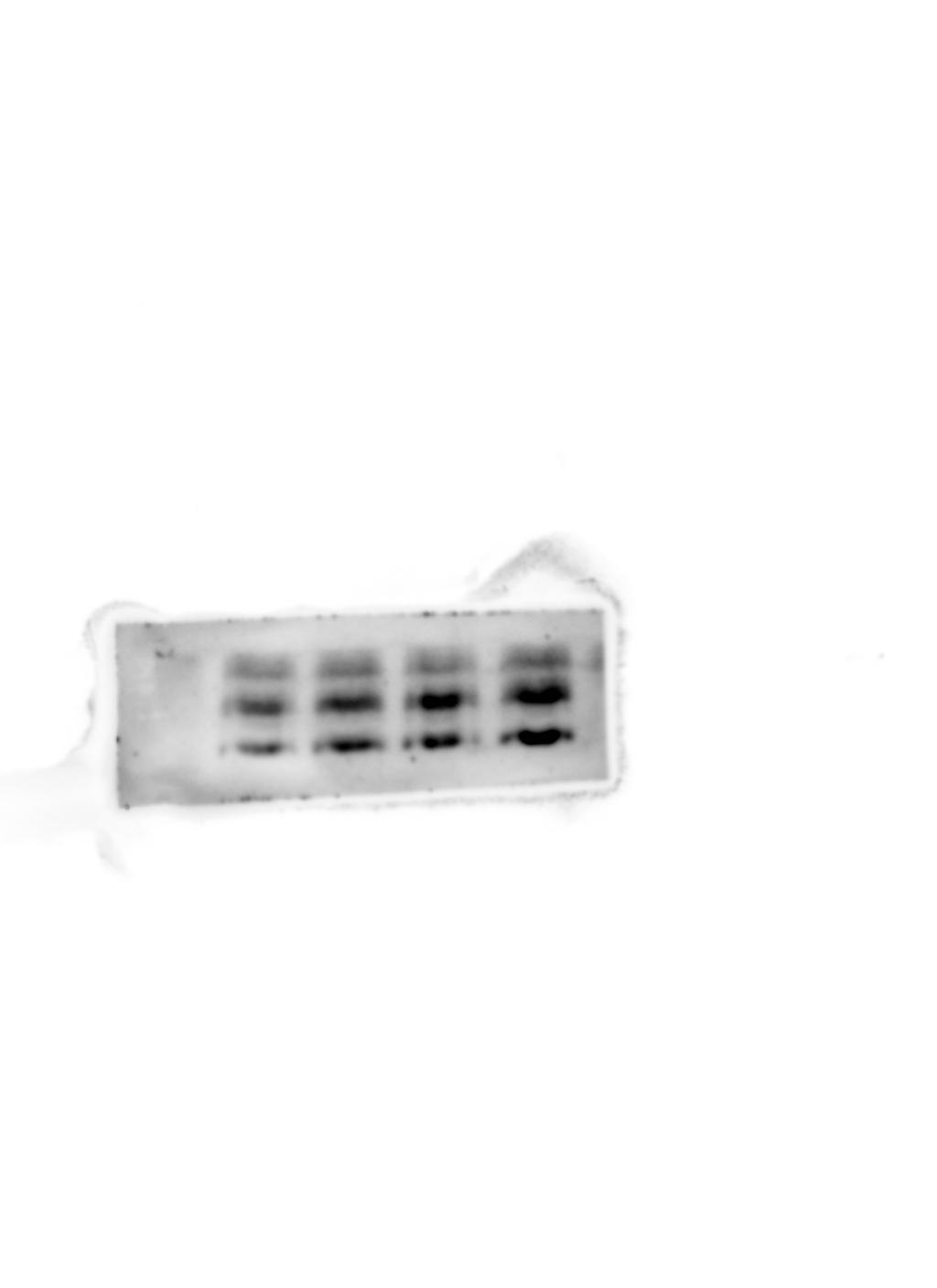

Supplement: Supplemental Information 2 [file peerj-10-14267-s002.zip › uncropped blots/figure2-uncropped blots/A-uncropped blots/p16-1.jpg]

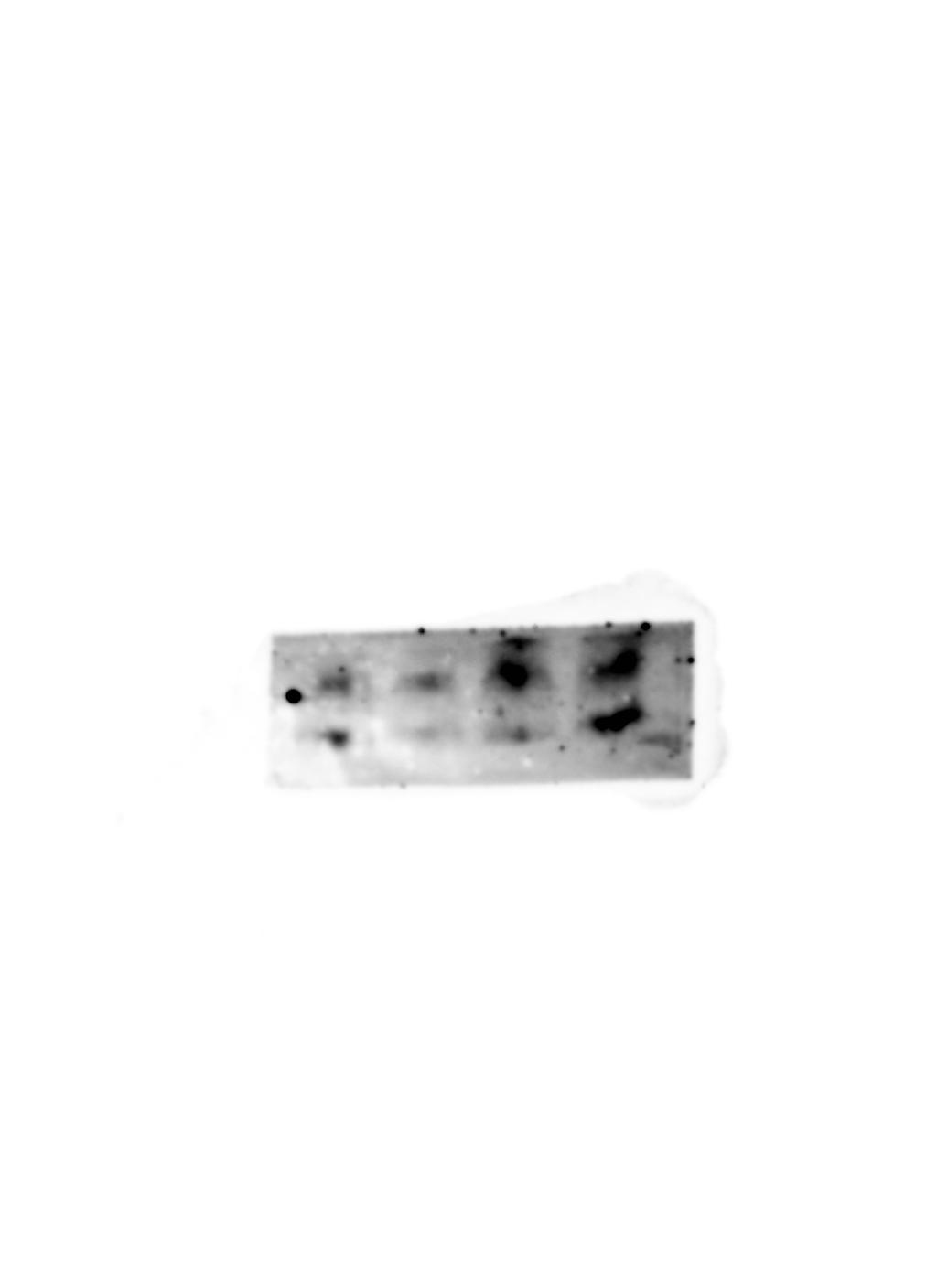

Supplement: Supplemental Information 2 [file peerj-10-14267-s002.zip › uncropped blots/figure2-uncropped blots/A-uncropped blots/p16-2.jpg]

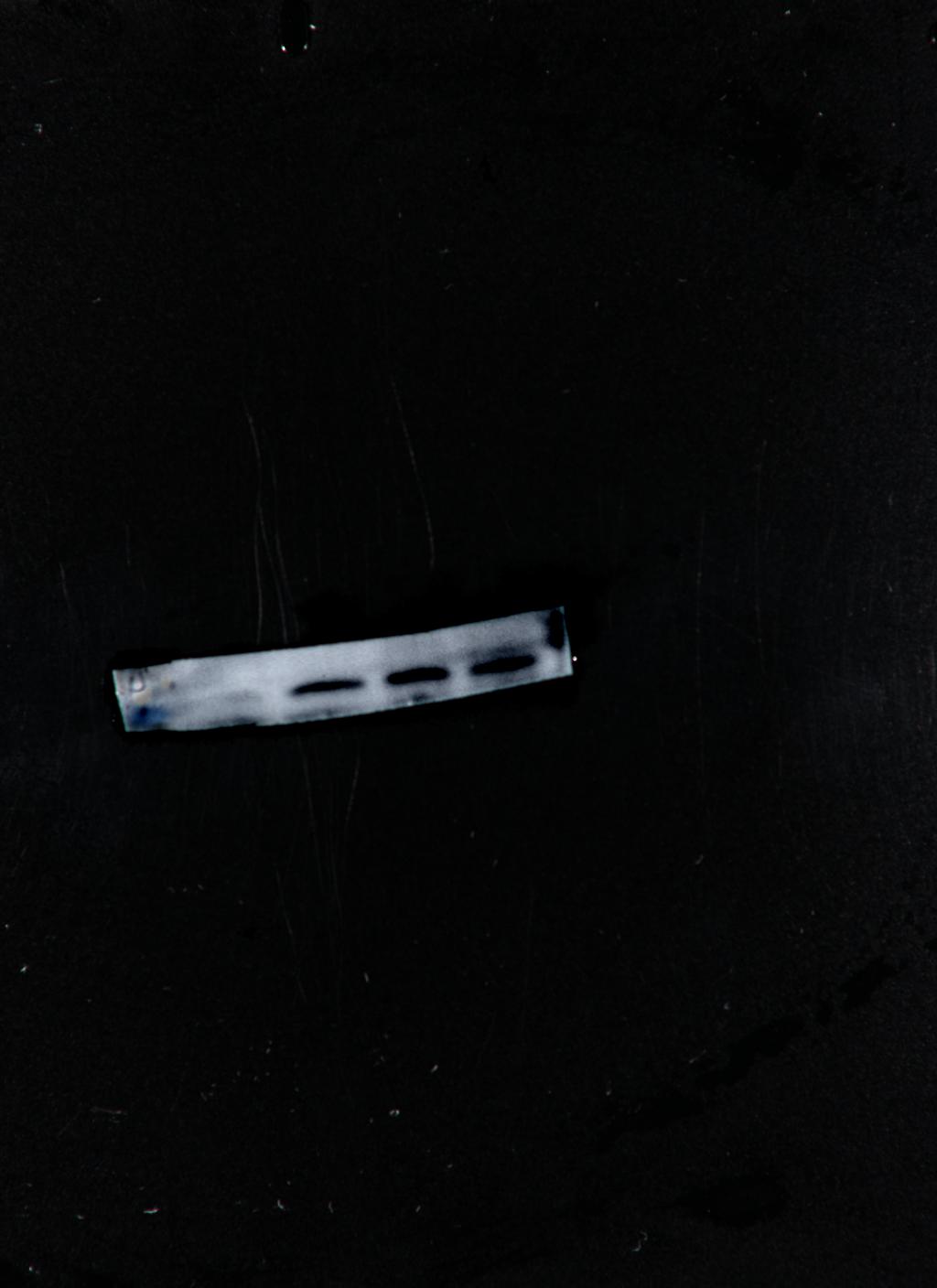

Supplement: Supplemental Information 2 [file peerj-10-14267-s002.zip › uncropped blots/figure2-uncropped blots/A-uncropped blots/p16-3.jpg]

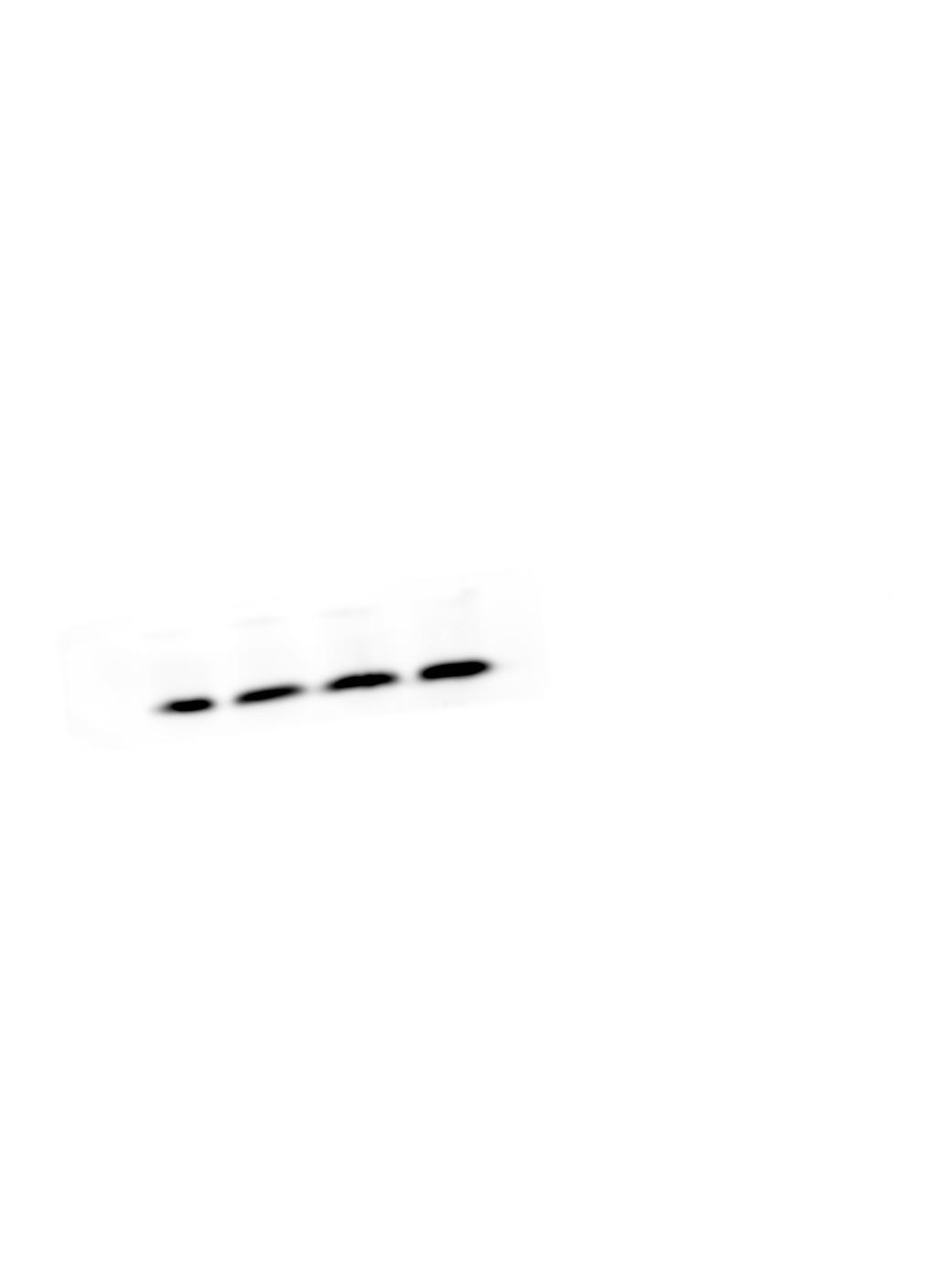

Supplement: Supplemental Information 2 [file peerj-10-14267-s002.zip › uncropped blots/figure2-uncropped blots/A-uncropped blots/p21-1.jpg]

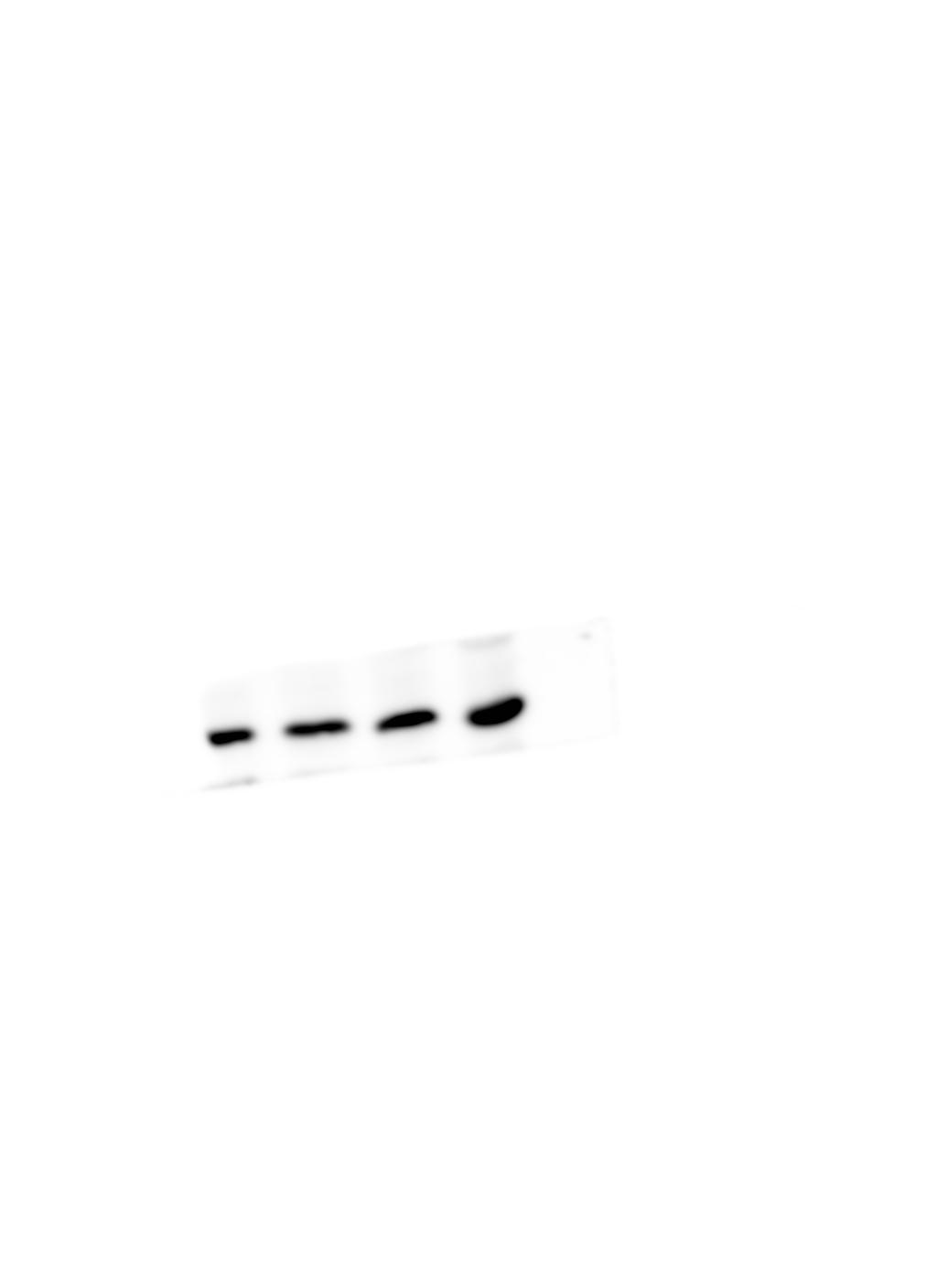

Supplement: Supplemental Information 2 [file peerj-10-14267-s002.zip › uncropped blots/figure2-uncropped blots/A-uncropped blots/p21-2.jpg]

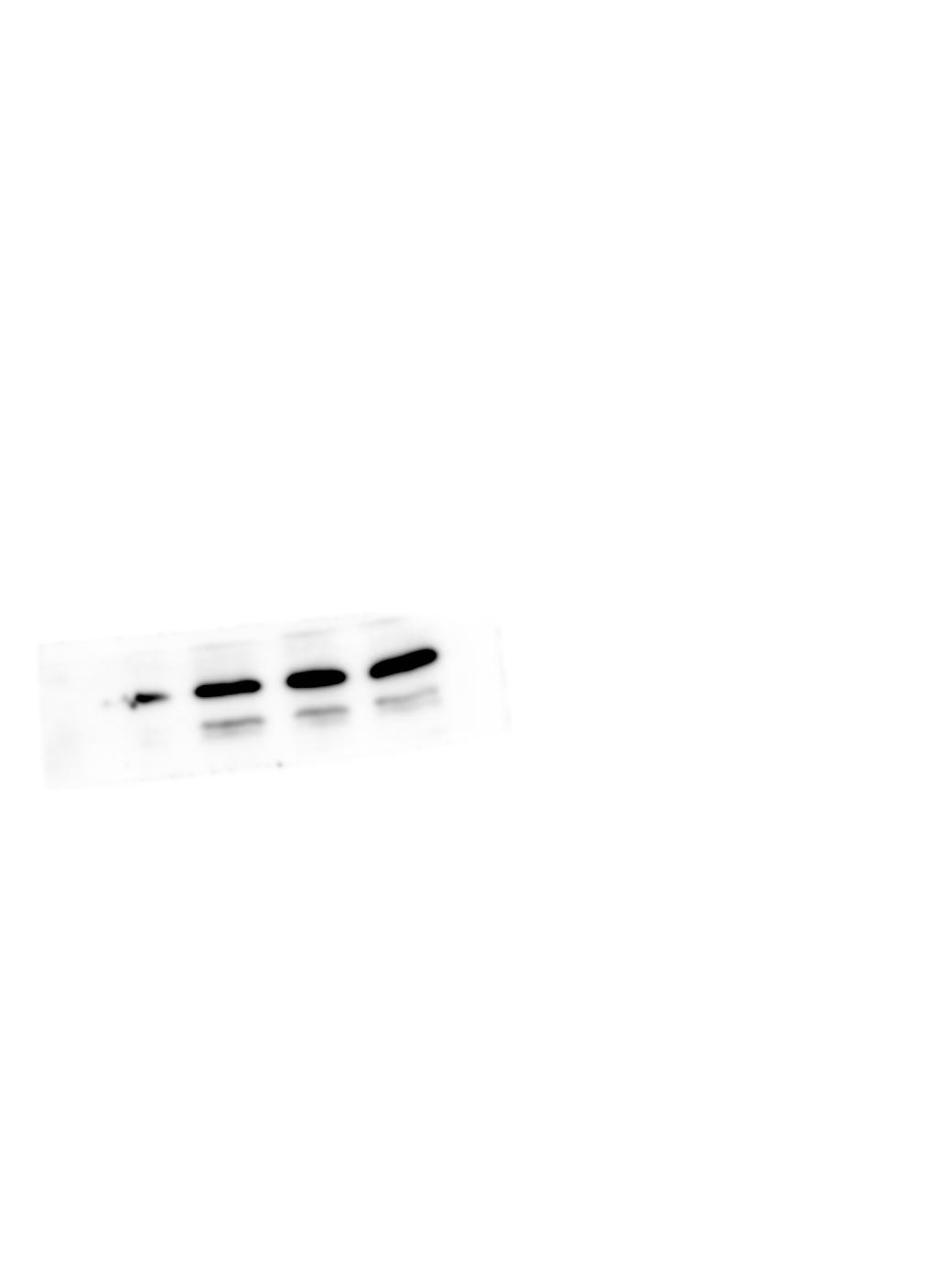

Supplement: Supplemental Information 2 [file peerj-10-14267-s002.zip › uncropped blots/figure2-uncropped blots/A-uncropped blots/p21-3.jpg]

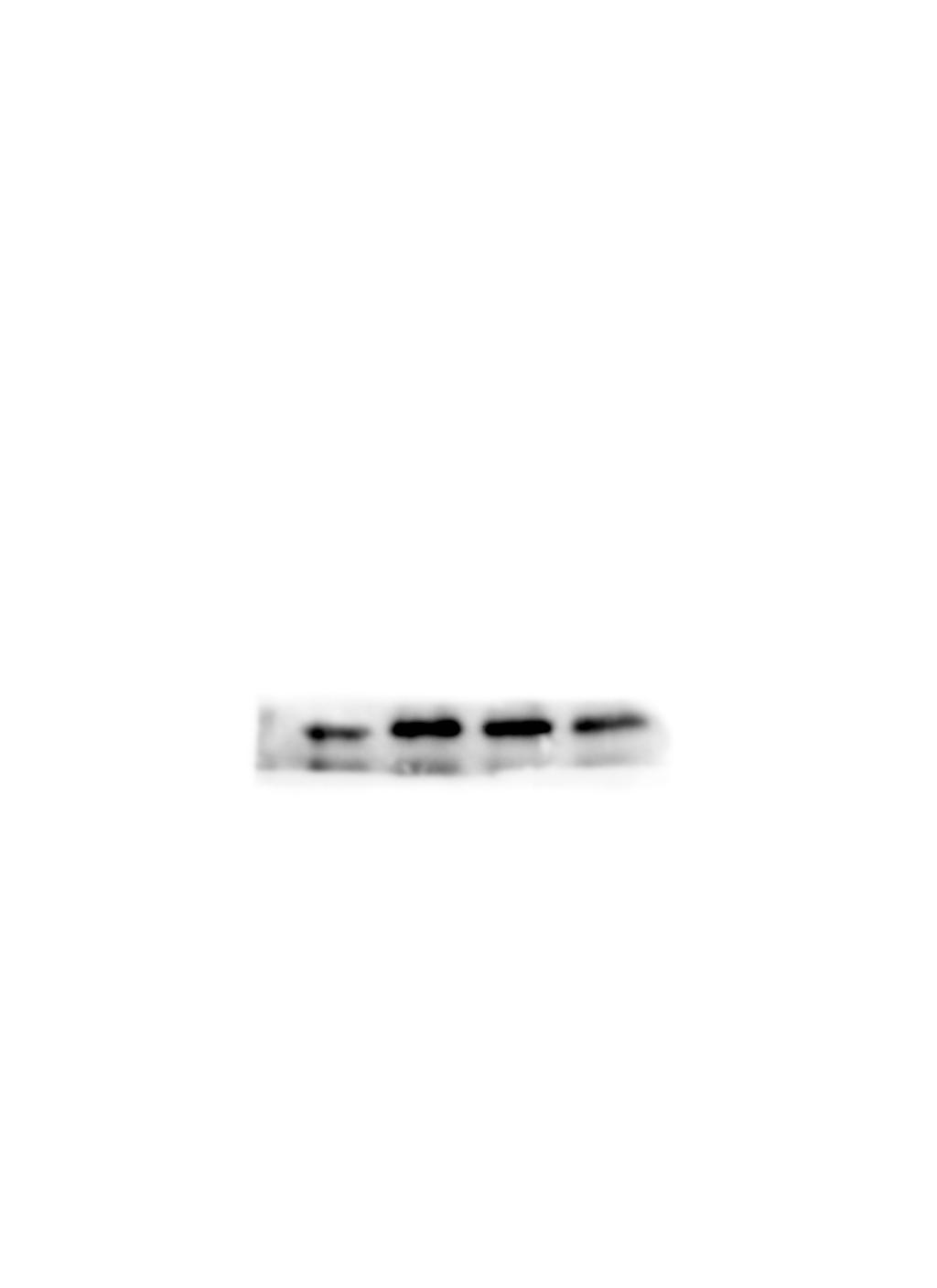

Supplement: Supplemental Information 2 [file peerj-10-14267-s002.zip › uncropped blots/figure2-uncropped blots/A-uncropped blots/p62-1.jpg]

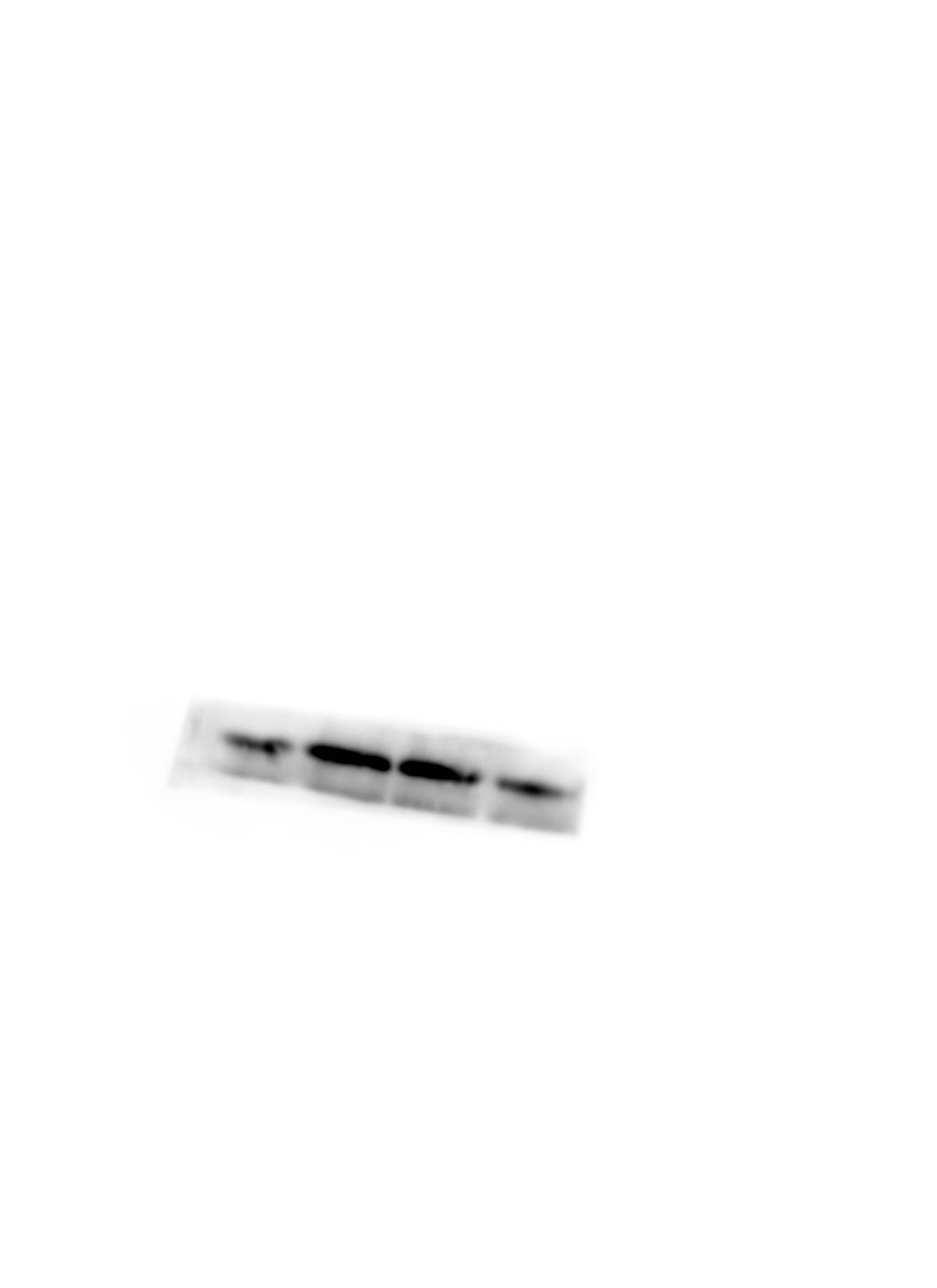

Supplement: Supplemental Information 2 [file peerj-10-14267-s002.zip › uncropped blots/figure2-uncropped blots/A-uncropped blots/p62-2.jpg]

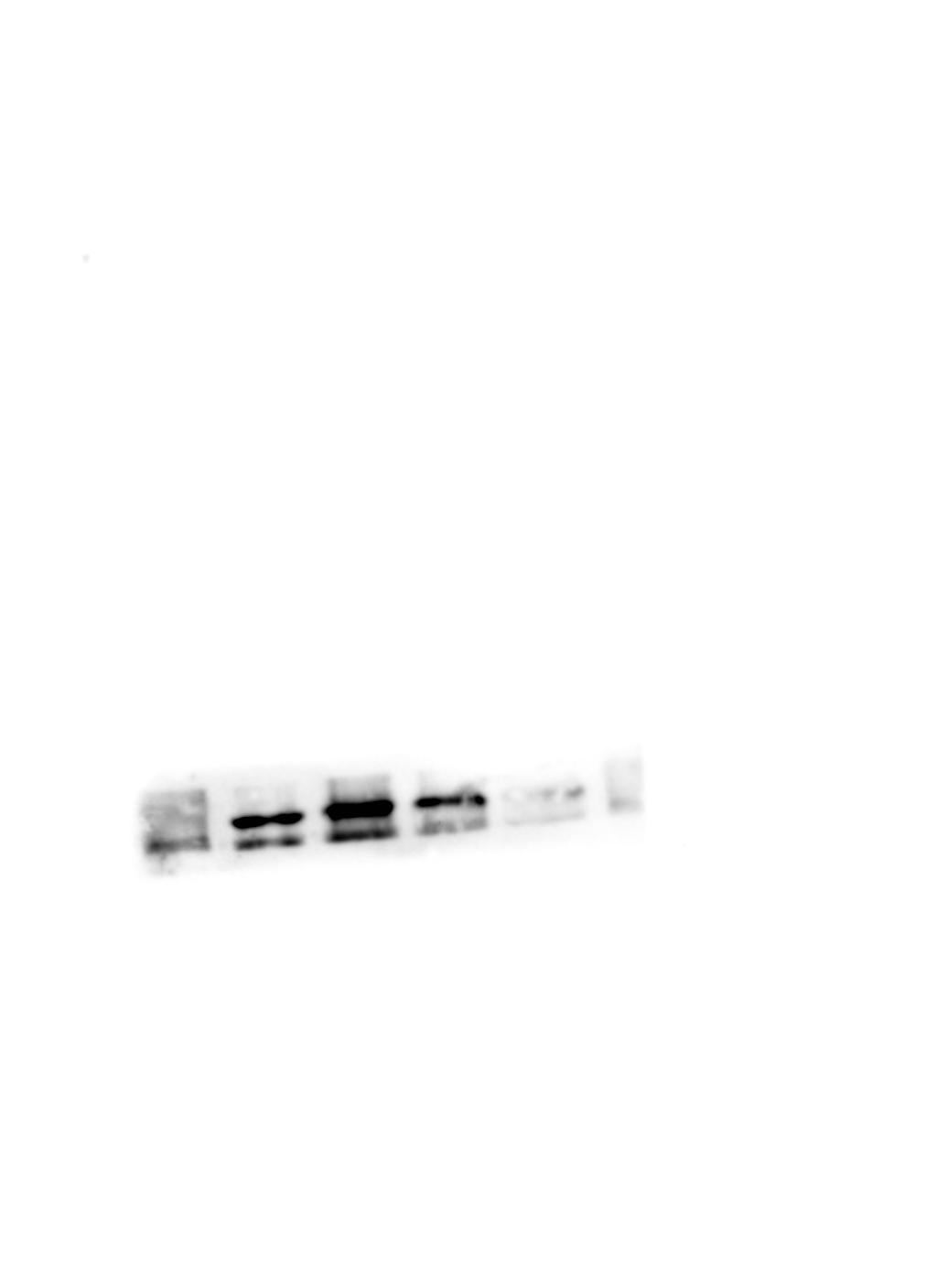

Supplement: Supplemental Information 2 [file peerj-10-14267-s002.zip › uncropped blots/figure2-uncropped blots/A-uncropped blots/p62-3.jpg]

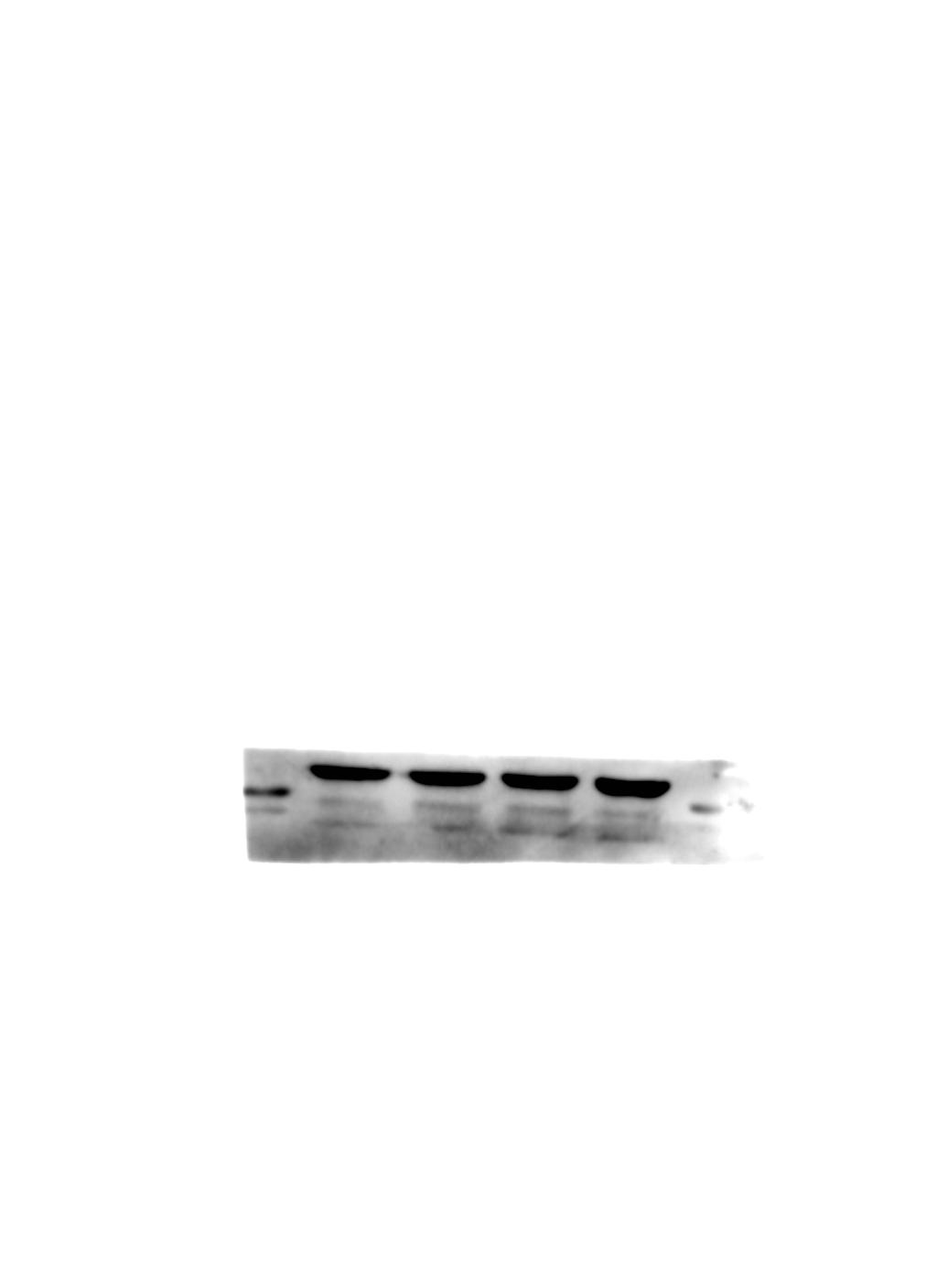

Supplement: Supplemental Information 2 [file peerj-10-14267-s002.zip › uncropped blots/figure2-uncropped blots/B-uncropped blots/actin-1.jpg]

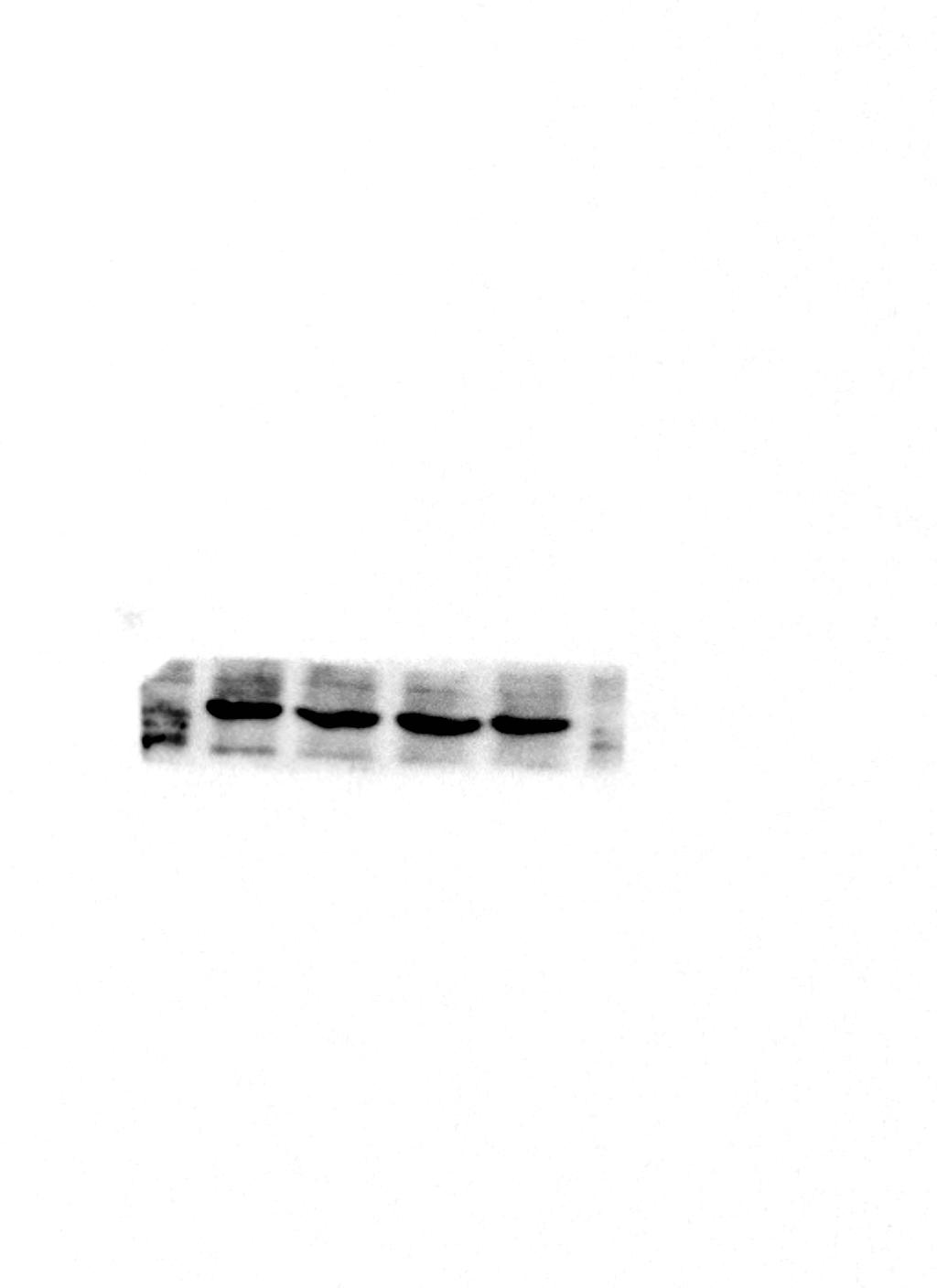

Supplement: Supplemental Information 2 [file peerj-10-14267-s002.zip › uncropped blots/figure2-uncropped blots/B-uncropped blots/actin-2.jpg]

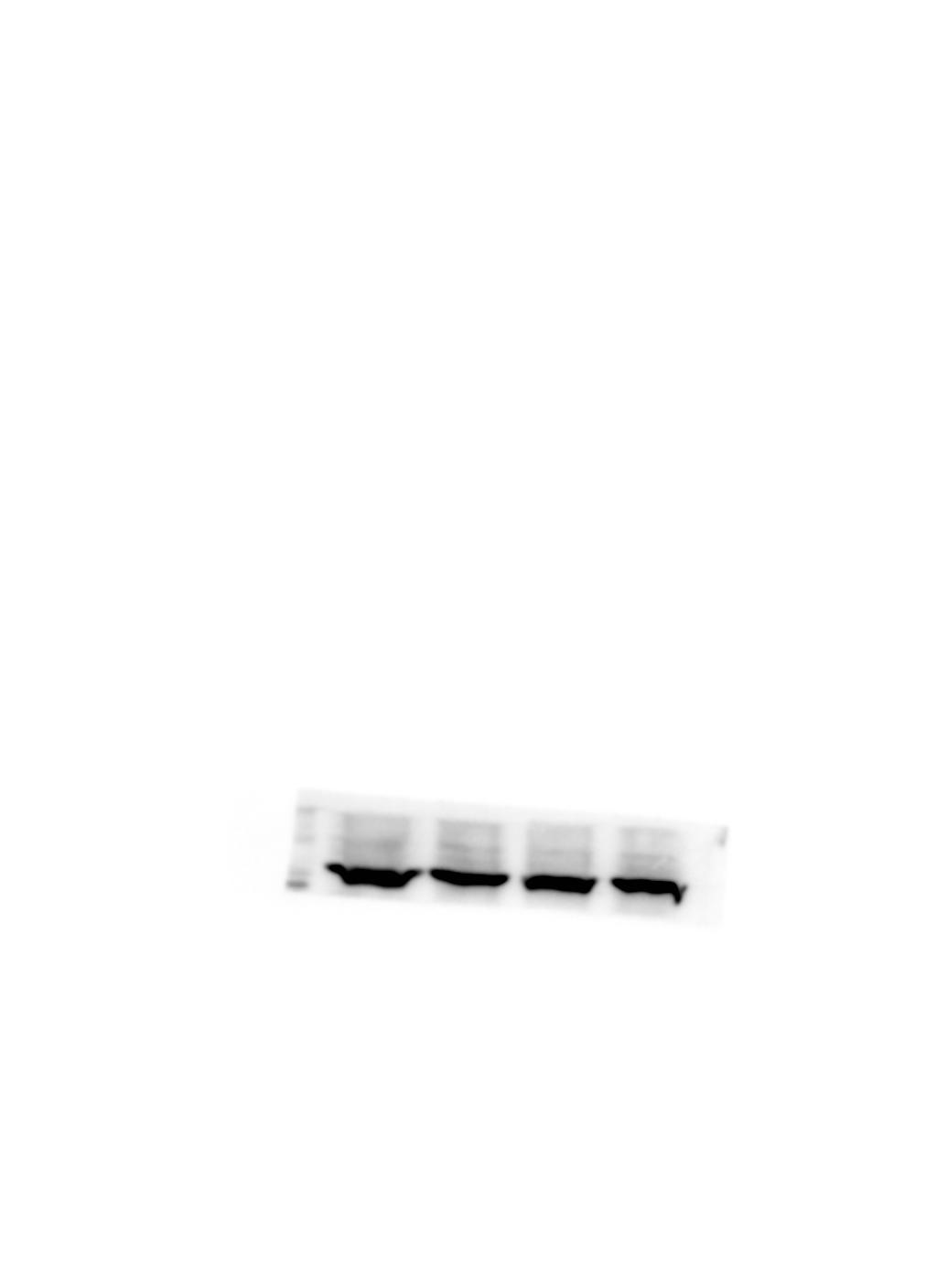

Supplement: Supplemental Information 2 [file peerj-10-14267-s002.zip › uncropped blots/figure2-uncropped blots/B-uncropped blots/actin-3.jpg]

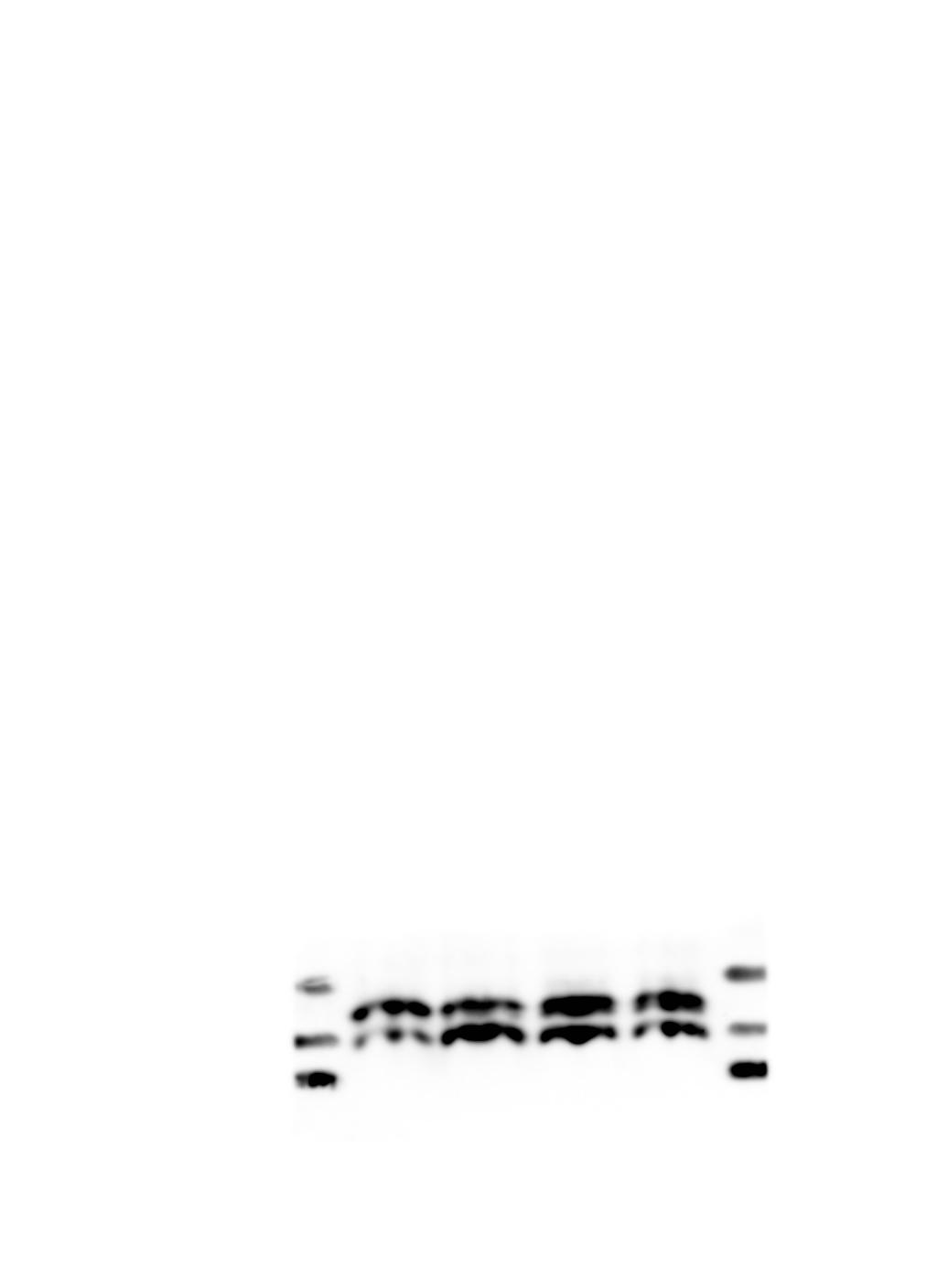

Supplement: Supplemental Information 2 [file peerj-10-14267-s002.zip › uncropped blots/figure2-uncropped blots/B-uncropped blots/LC3-1.jpg]

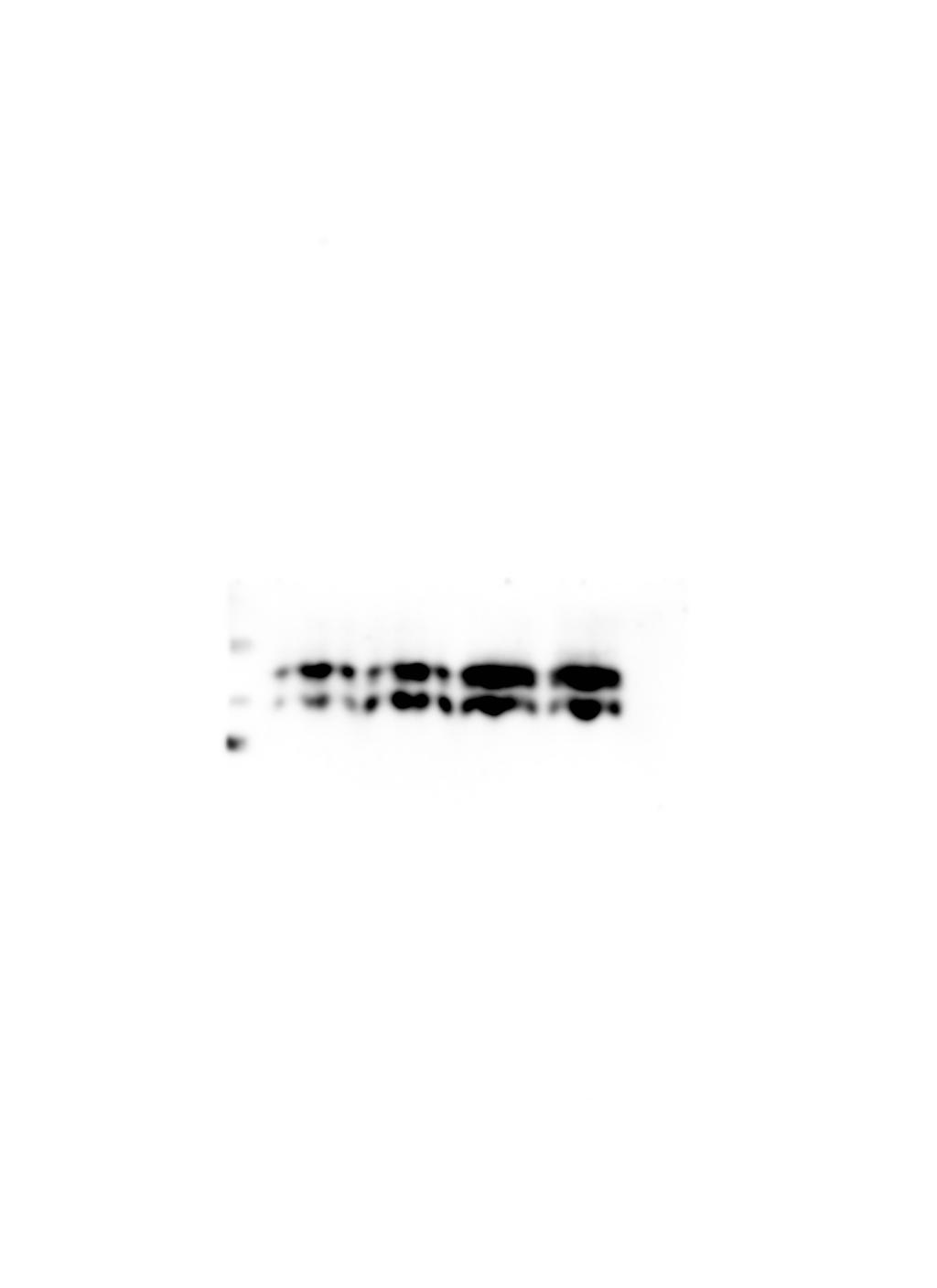

Supplement: Supplemental Information 2 [file peerj-10-14267-s002.zip › uncropped blots/figure2-uncropped blots/B-uncropped blots/LC3-2.jpg]

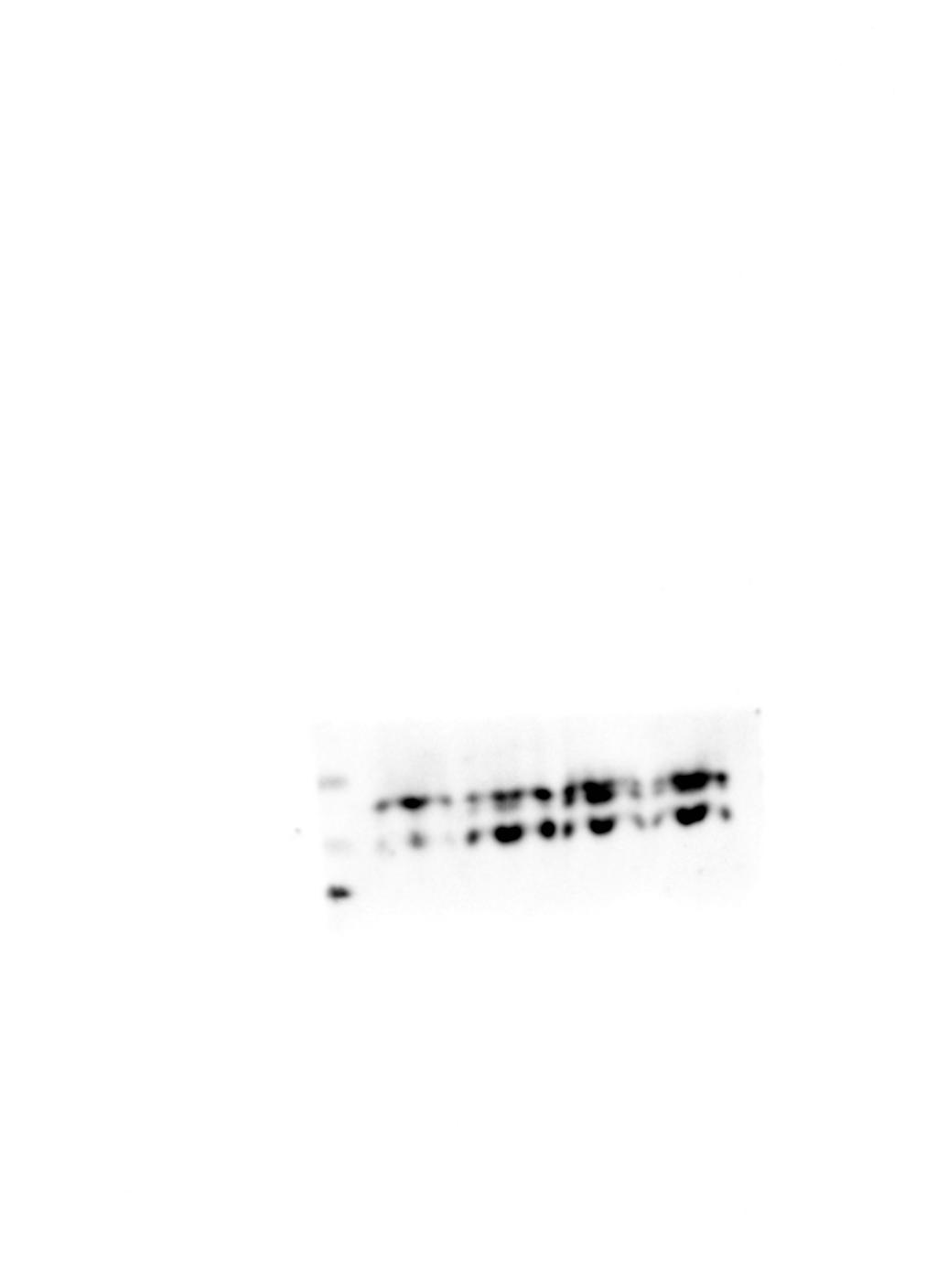

Supplement: Supplemental Information 2 [file peerj-10-14267-s002.zip › uncropped blots/figure2-uncropped blots/B-uncropped blots/LC3-3.jpg]

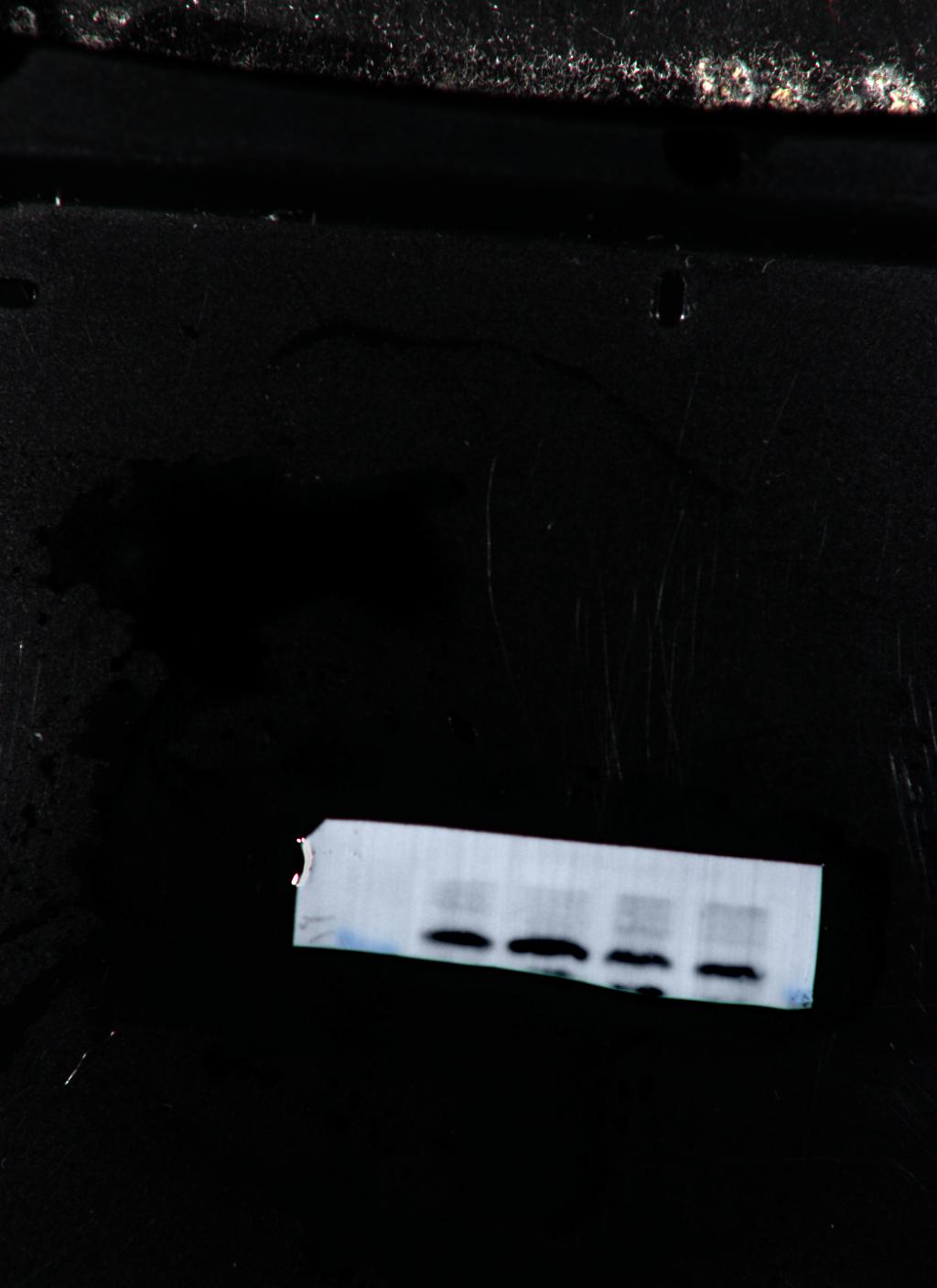

Supplement: Supplemental Information 2 [file peerj-10-14267-s002.zip › uncropped blots/figure2-uncropped blots/B-uncropped blots/p16-1.jpg]

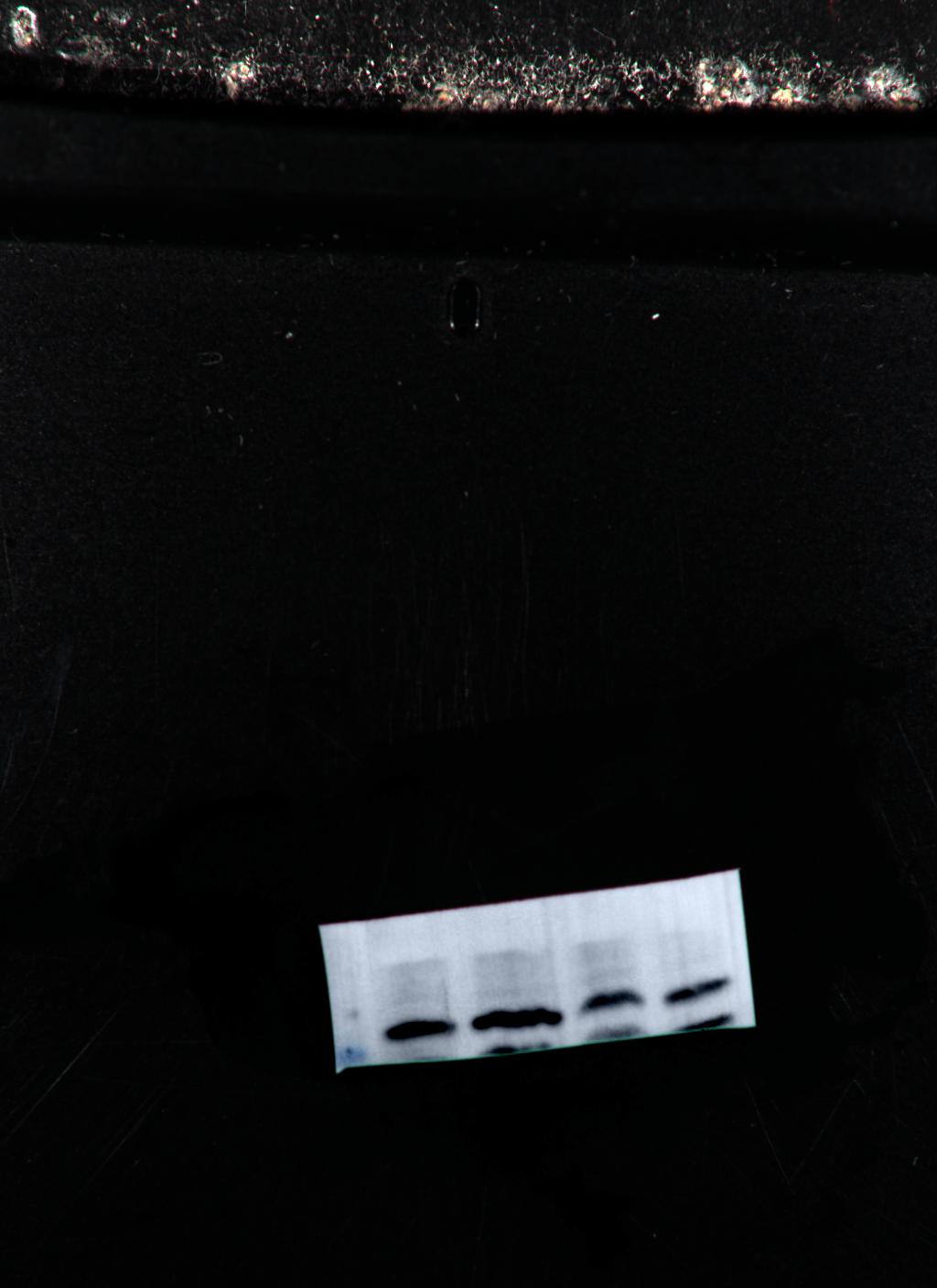

Supplement: Supplemental Information 2 [file peerj-10-14267-s002.zip › uncropped blots/figure2-uncropped blots/B-uncropped blots/p16-2.jpg]

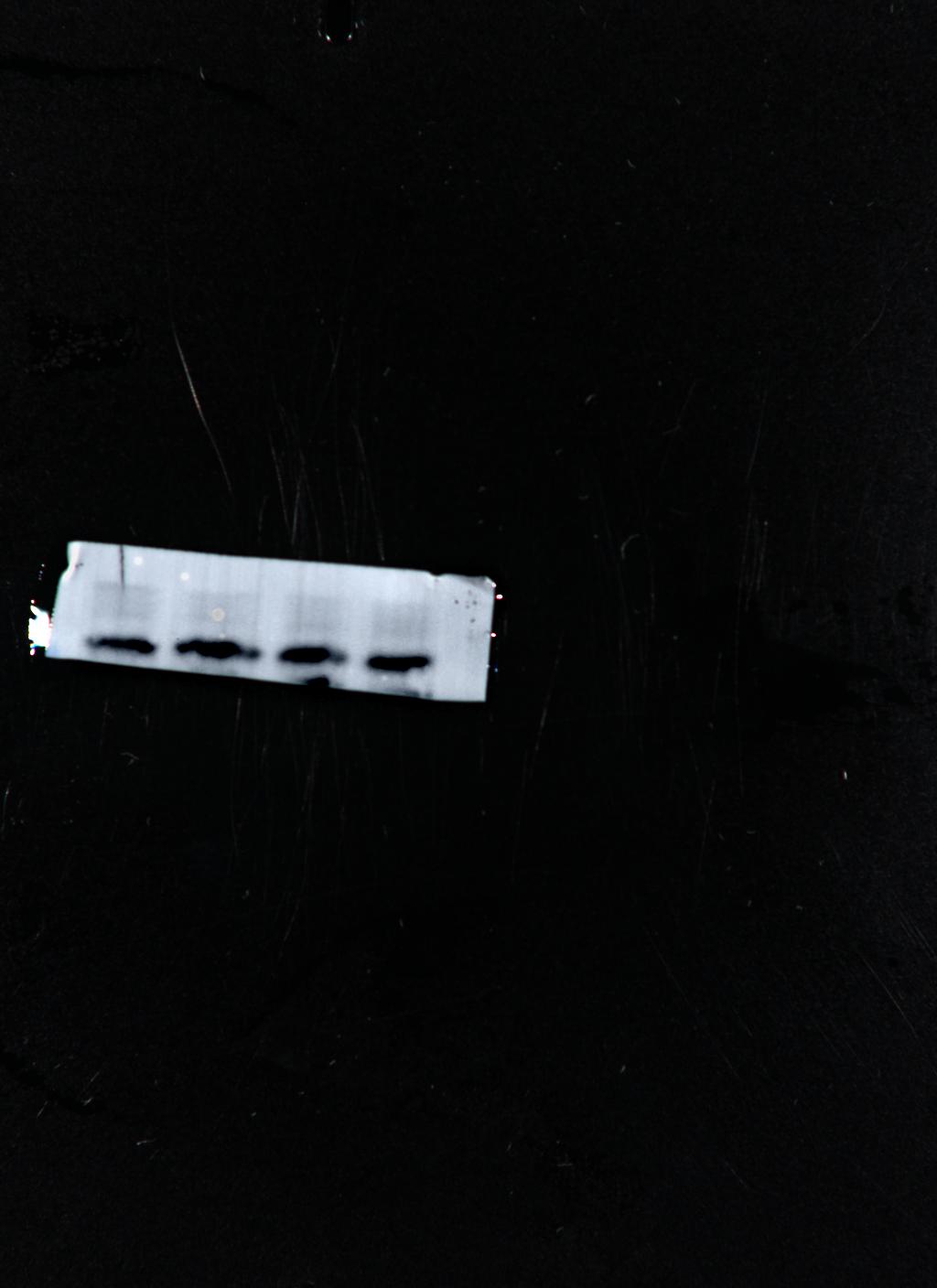

Supplement: Supplemental Information 2 [file peerj-10-14267-s002.zip › uncropped blots/figure2-uncropped blots/B-uncropped blots/p16-3.jpg]

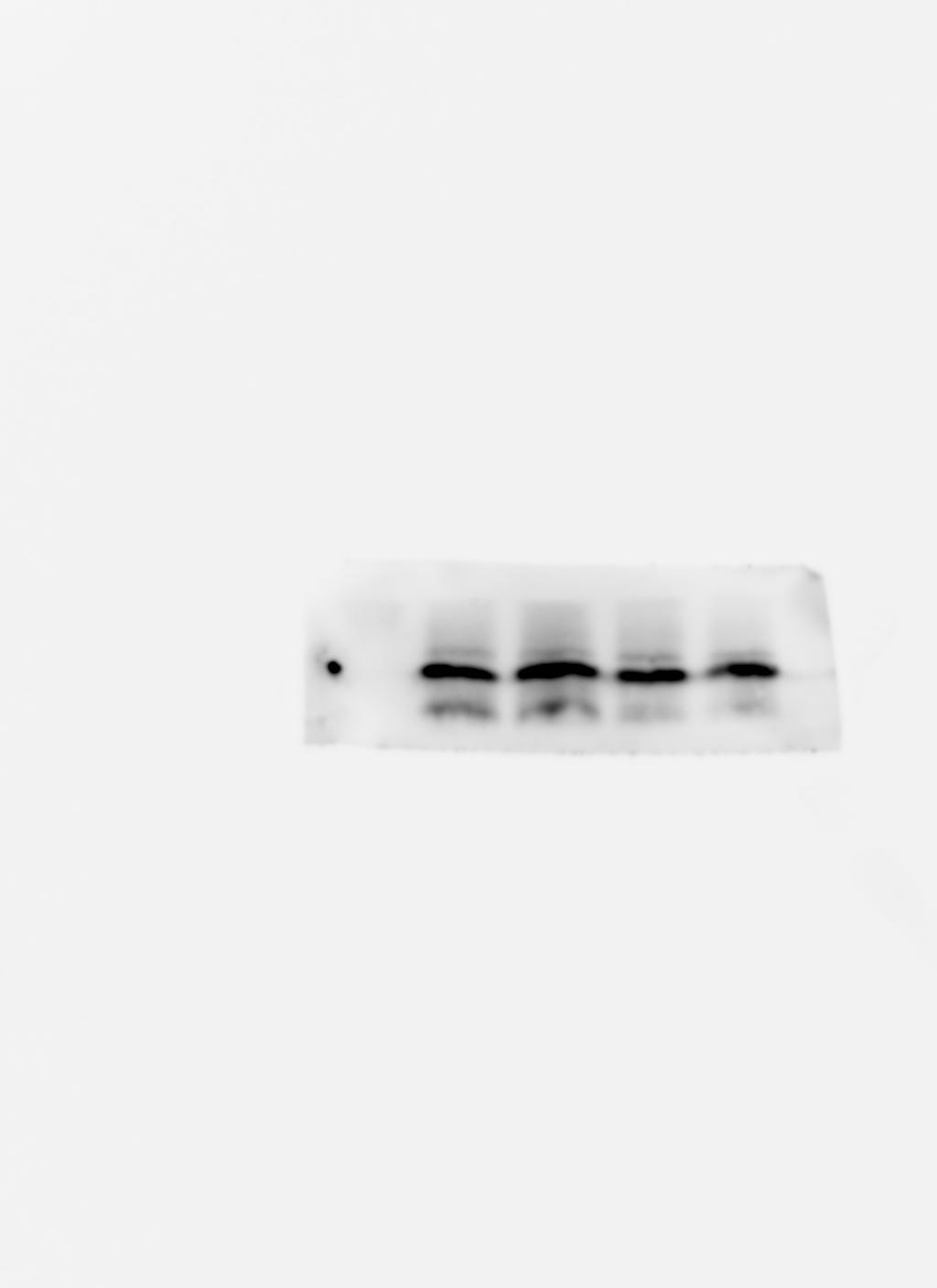

Supplement: Supplemental Information 2 [file peerj-10-14267-s002.zip › uncropped blots/figure2-uncropped blots/B-uncropped blots/p21-1.jpg]

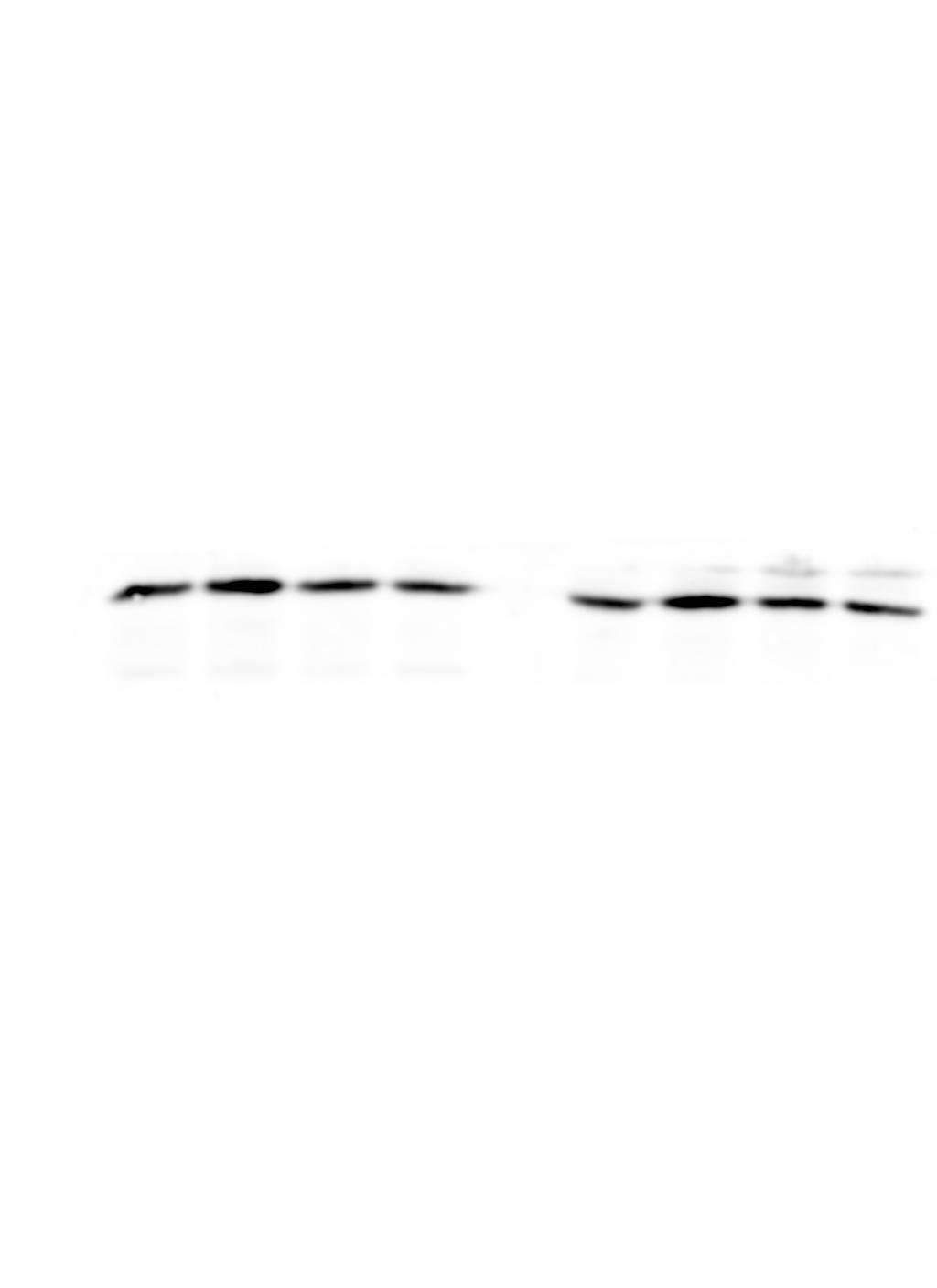

Supplement: Supplemental Information 2 [file peerj-10-14267-s002.zip › uncropped blots/figure2-uncropped blots/B-uncropped blots/p21-2.3.jpg]

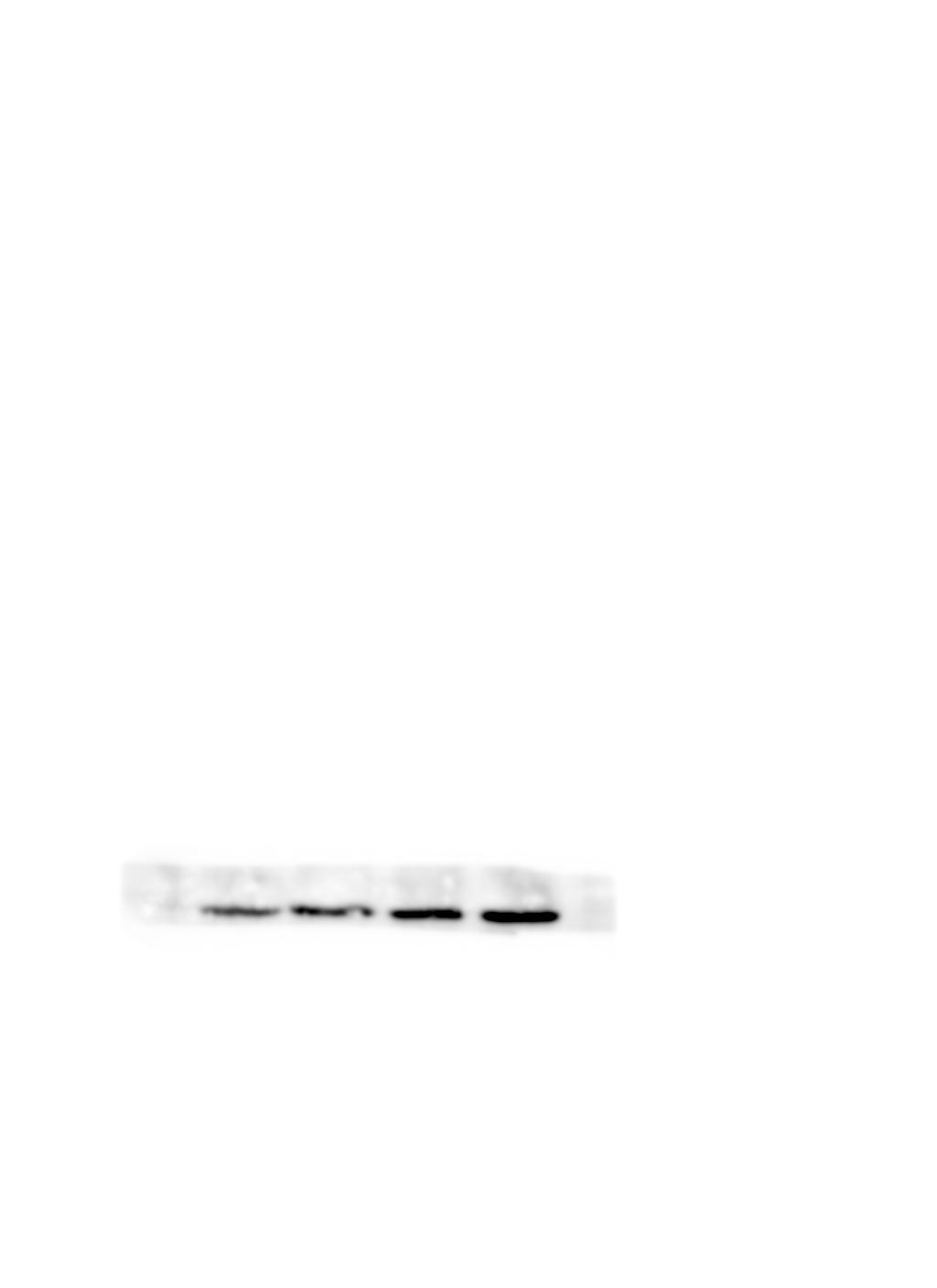

Supplement: Supplemental Information 2 [file peerj-10-14267-s002.zip › uncropped blots/figure2-uncropped blots/B-uncropped blots/p62-1.jpg]

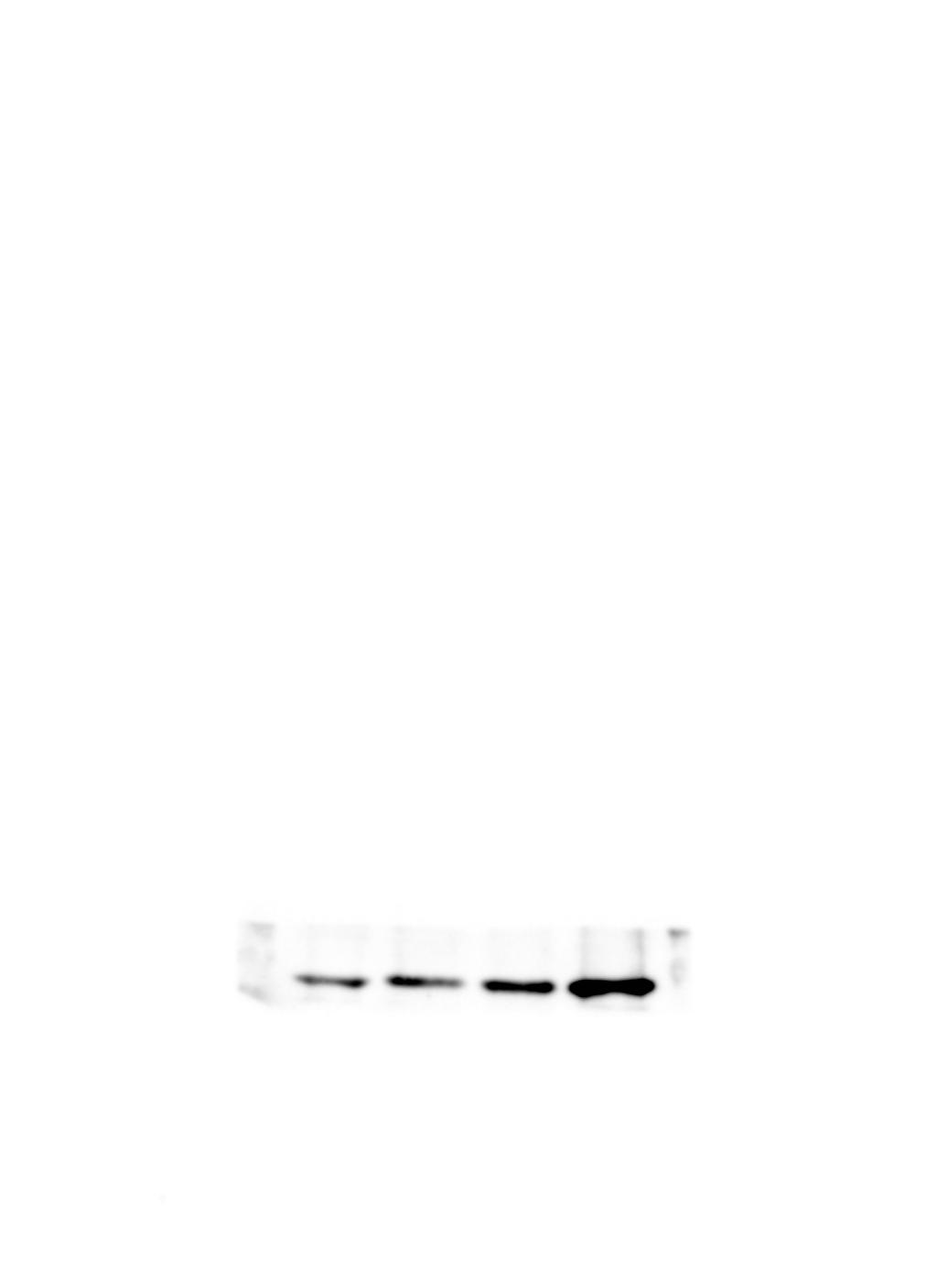

Supplement: Supplemental Information 2 [file peerj-10-14267-s002.zip › uncropped blots/figure2-uncropped blots/B-uncropped blots/p62-2.jpg]

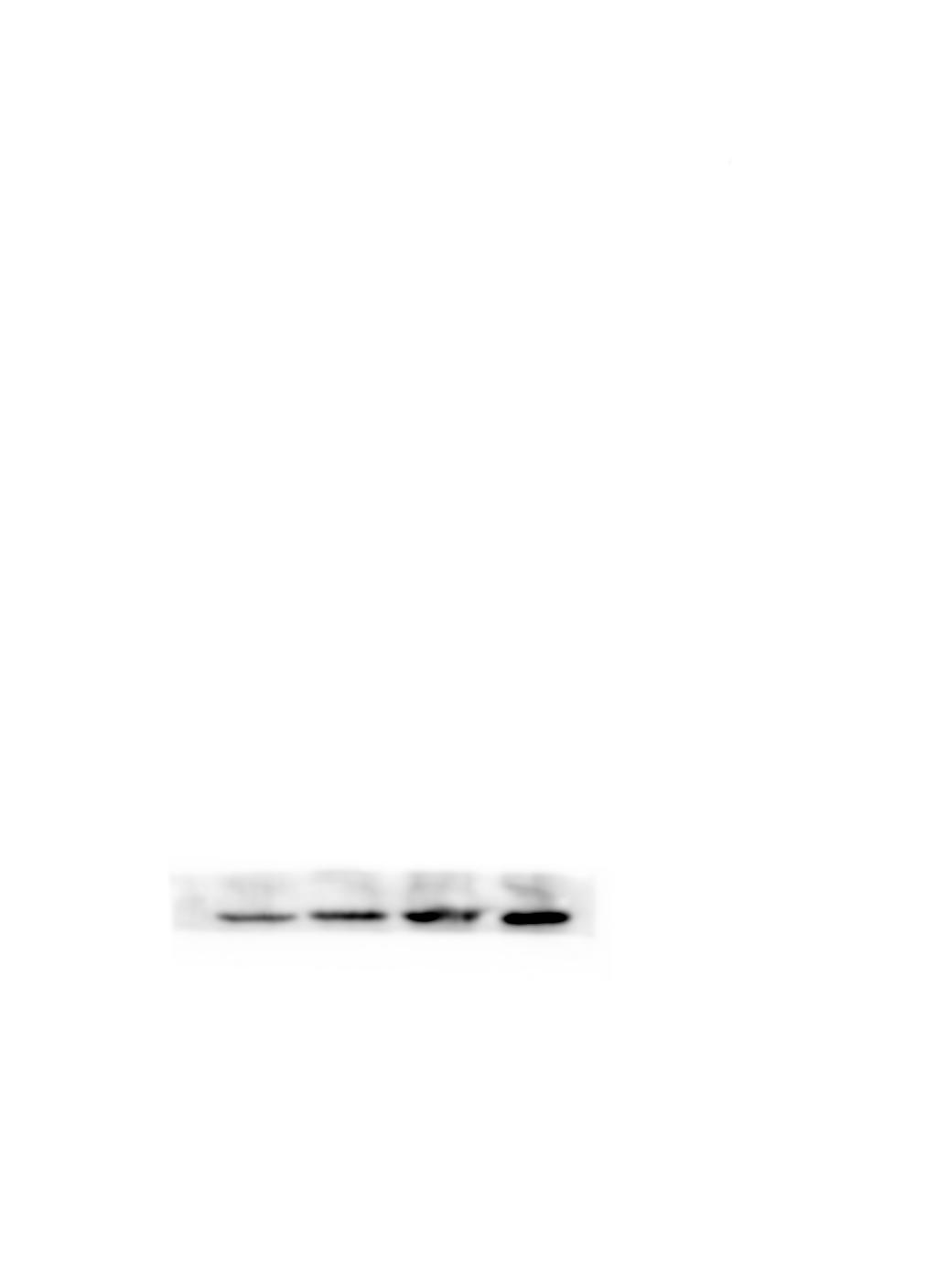

Supplement: Supplemental Information 2 [file peerj-10-14267-s002.zip › uncropped blots/figure2-uncropped blots/B-uncropped blots/p62-3.jpg]

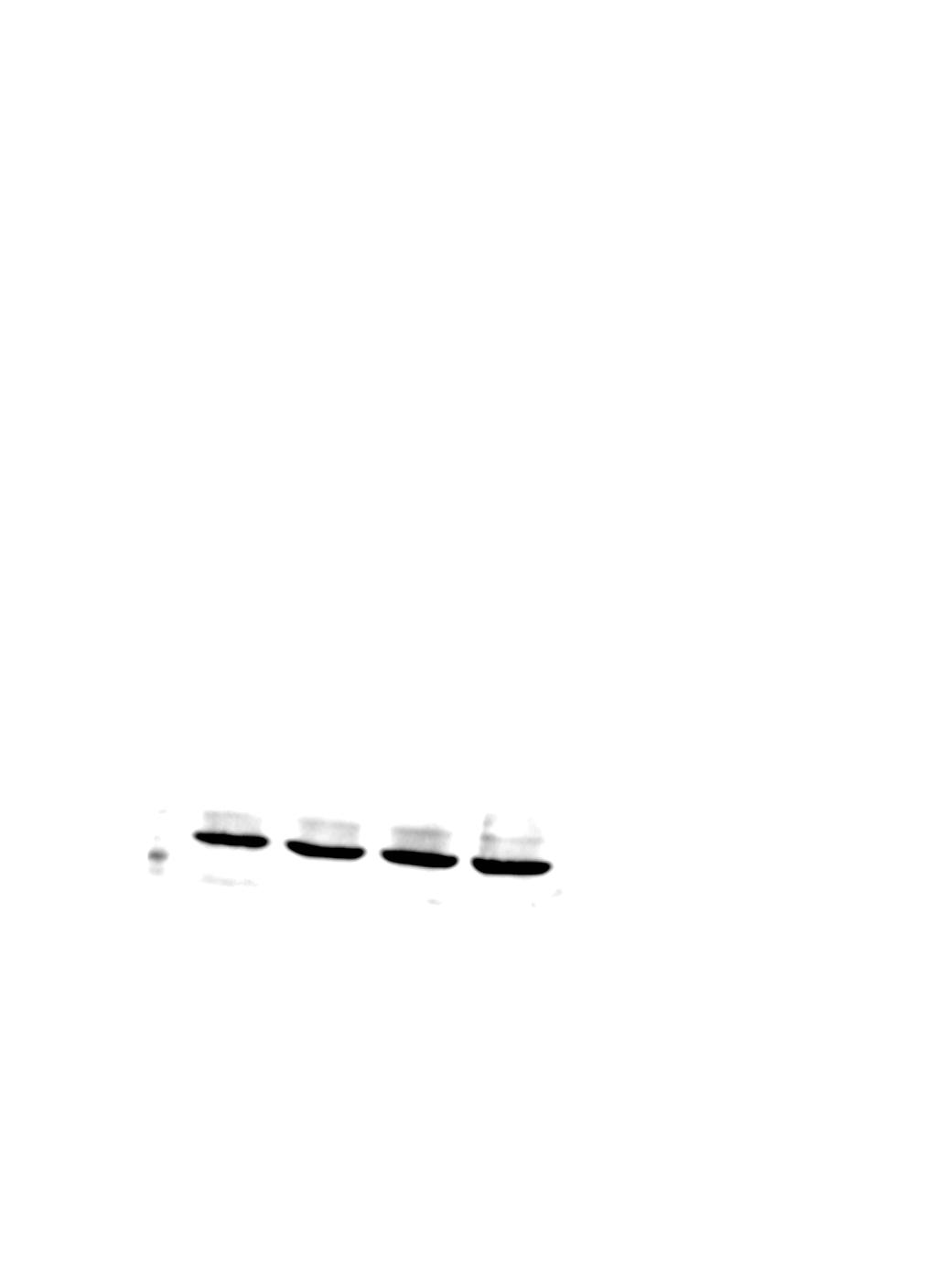

Supplement: Supplemental Information 2 [file peerj-10-14267-s002.zip › uncropped blots/figure3-uncropped blots/A-uncropped blots/actin-1.jpg]

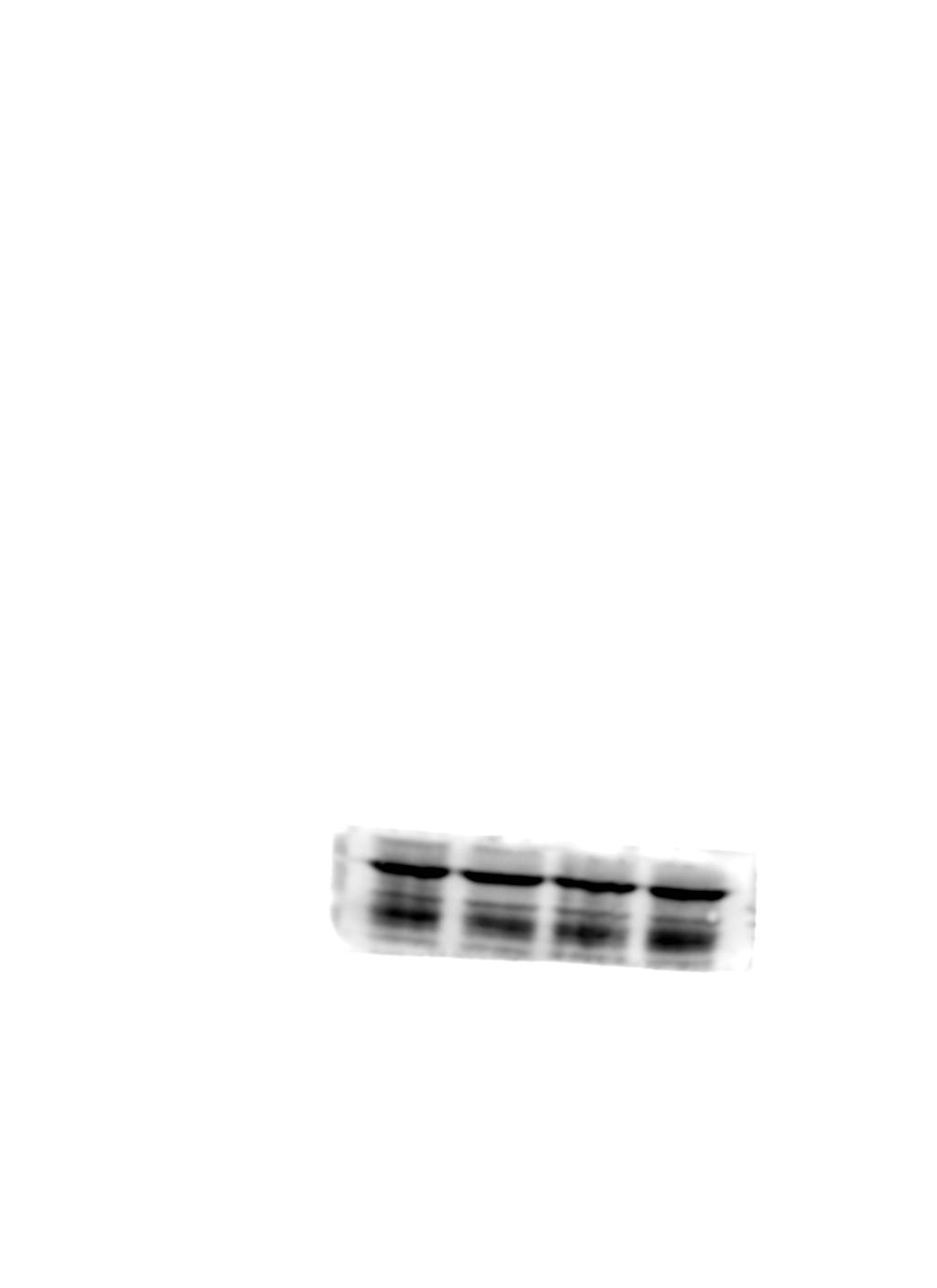

Supplement: Supplemental Information 2 [file peerj-10-14267-s002.zip › uncropped blots/figure3-uncropped blots/A-uncropped blots/actin-2.jpg]

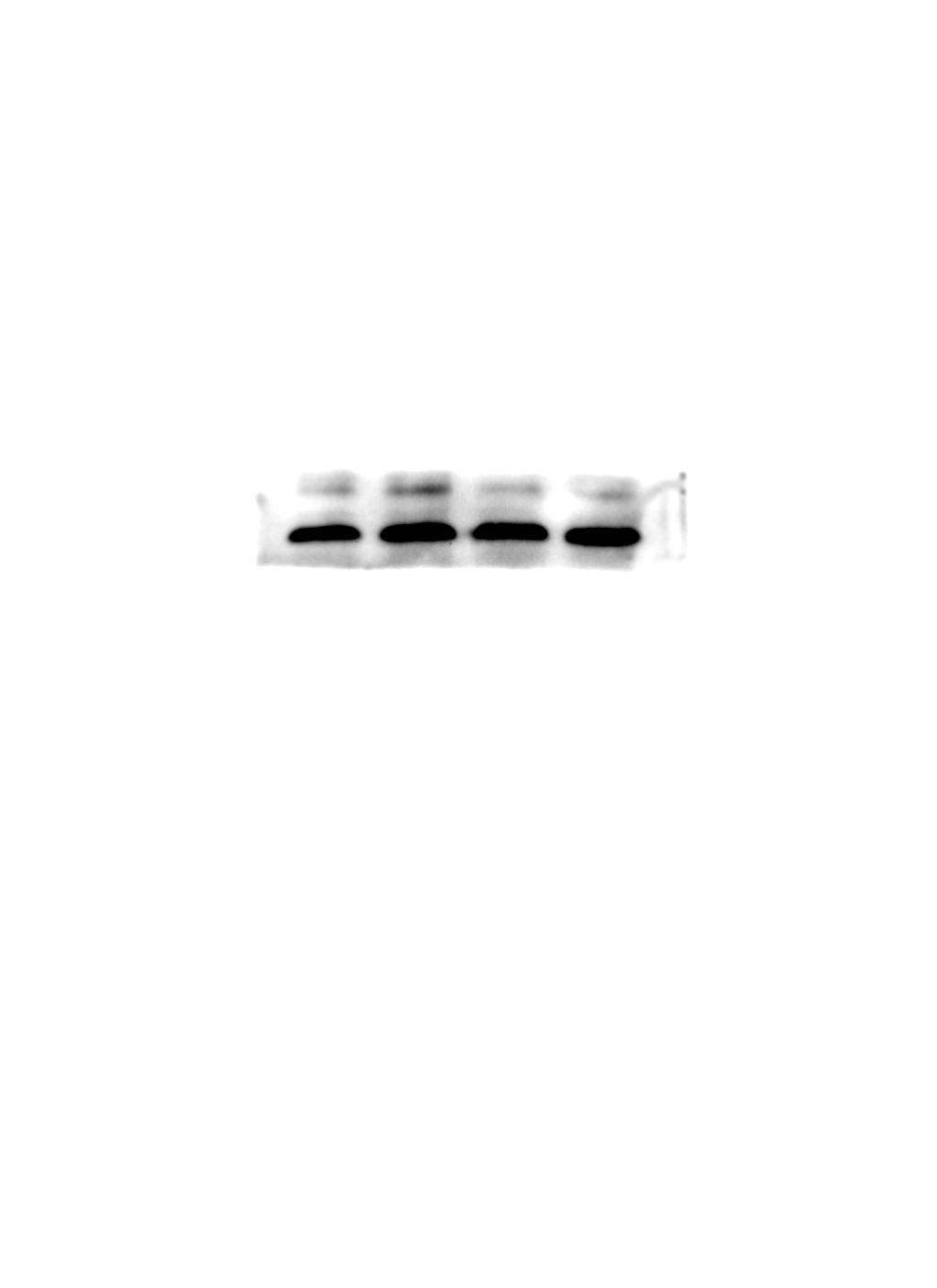

Supplement: Supplemental Information 2 [file peerj-10-14267-s002.zip › uncropped blots/figure3-uncropped blots/A-uncropped blots/actin-3.jpg]

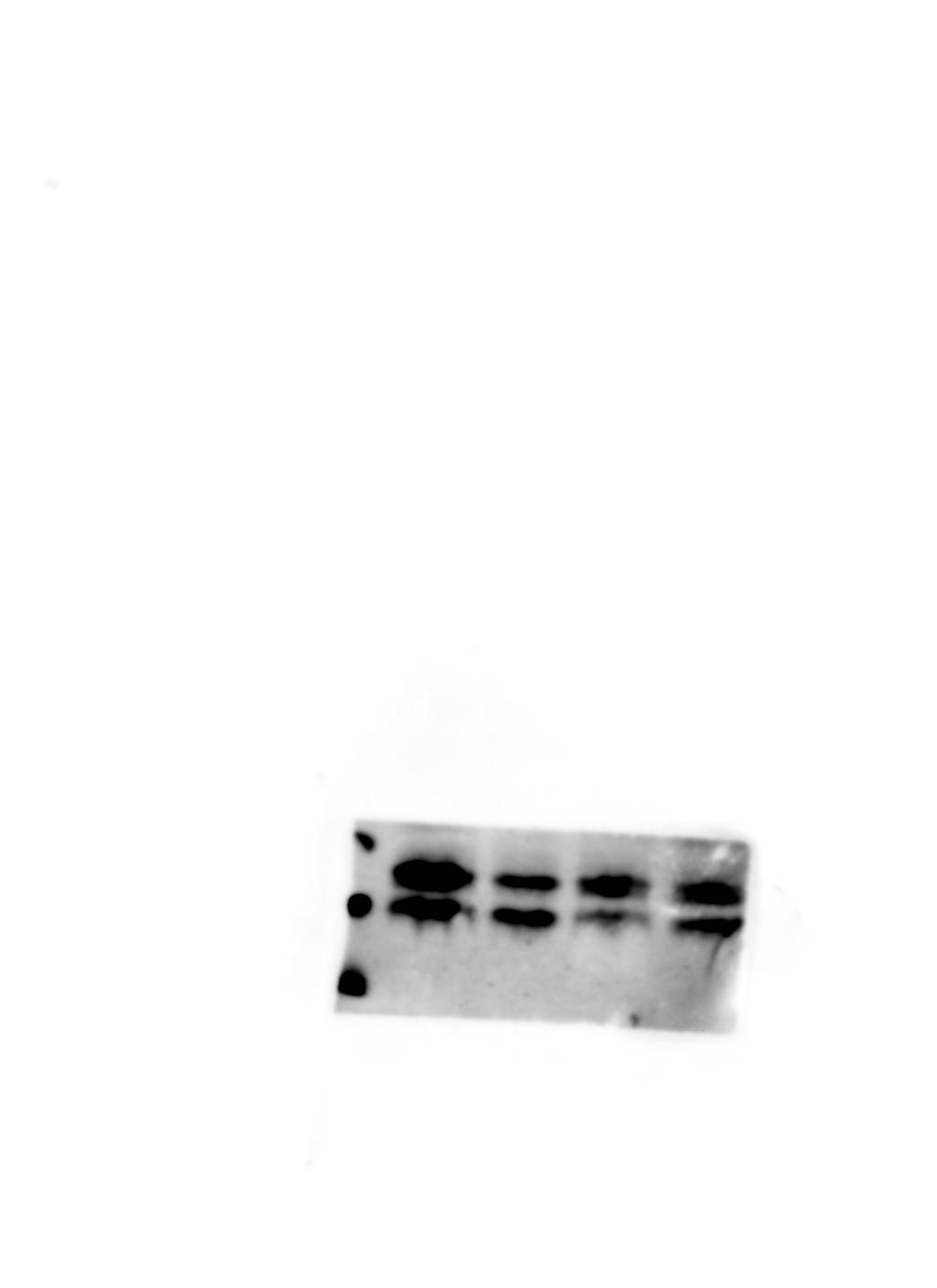

Supplement: Supplemental Information 2 [file peerj-10-14267-s002.zip › uncropped blots/figure3-uncropped blots/A-uncropped blots/LC3-1.jpg]

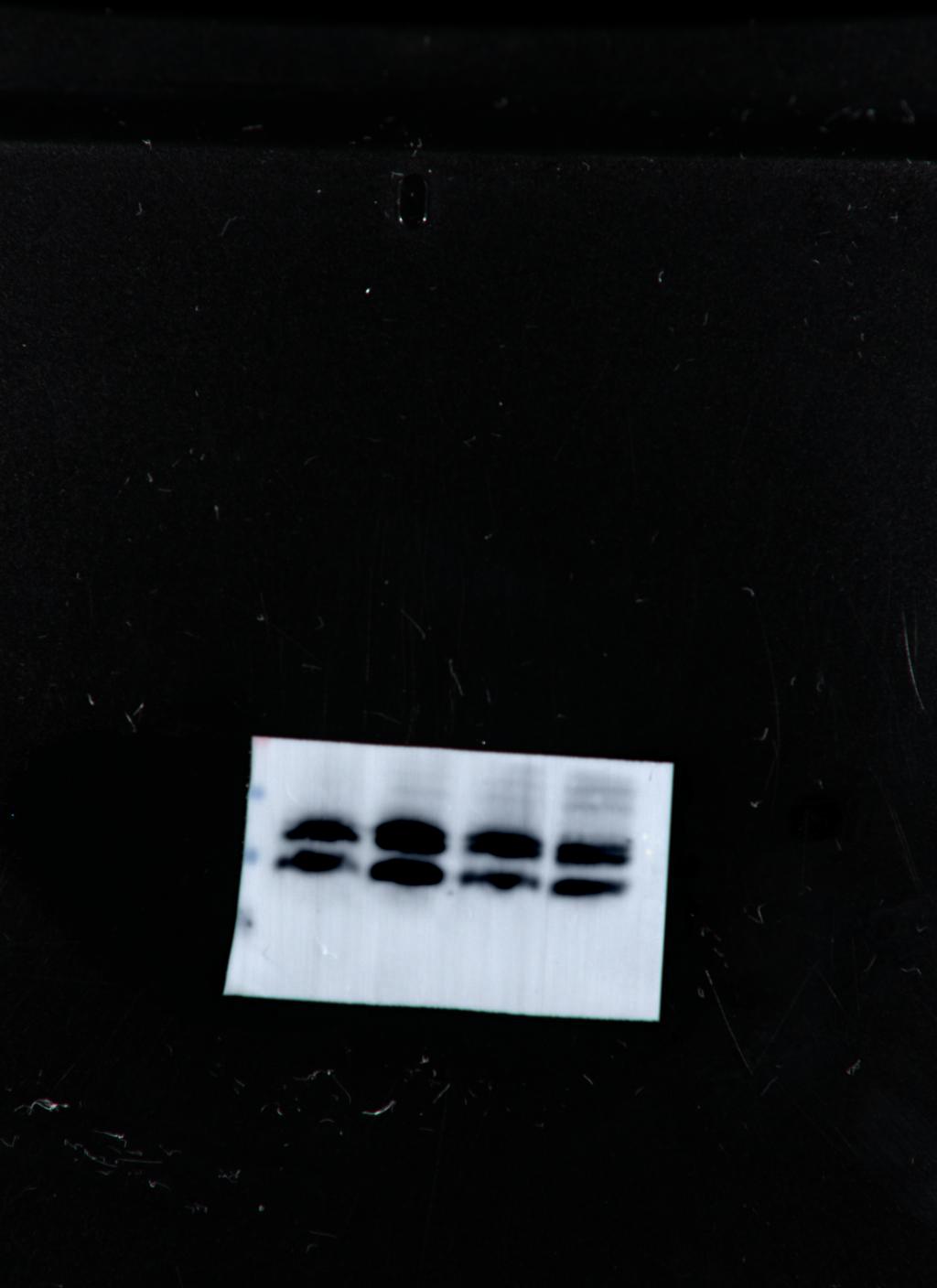

Supplement: Supplemental Information 2 [file peerj-10-14267-s002.zip › uncropped blots/figure3-uncropped blots/A-uncropped blots/LC3-2.jpg]

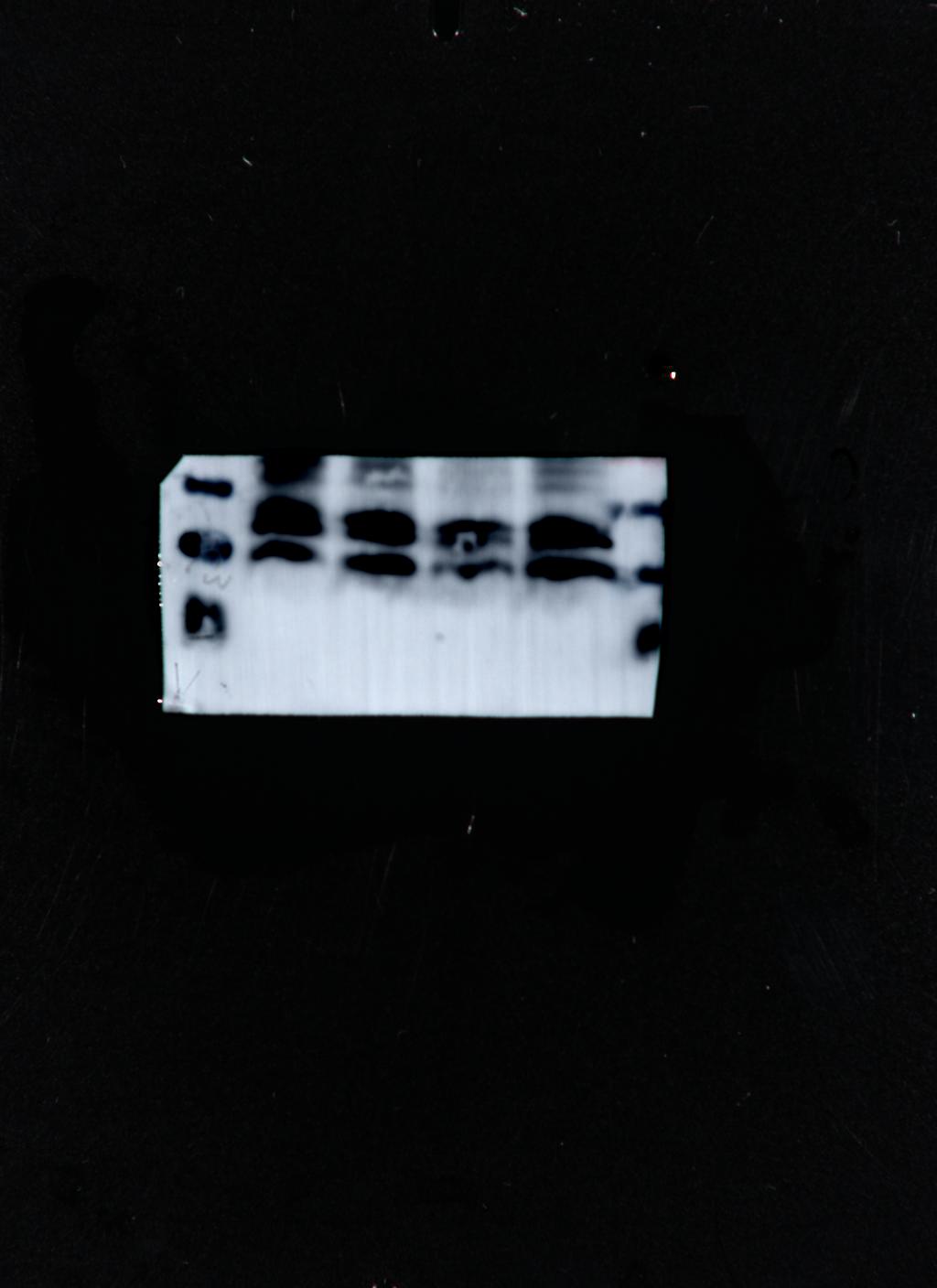

Supplement: Supplemental Information 2 [file peerj-10-14267-s002.zip › uncropped blots/figure3-uncropped blots/A-uncropped blots/LC3-3.jpg]

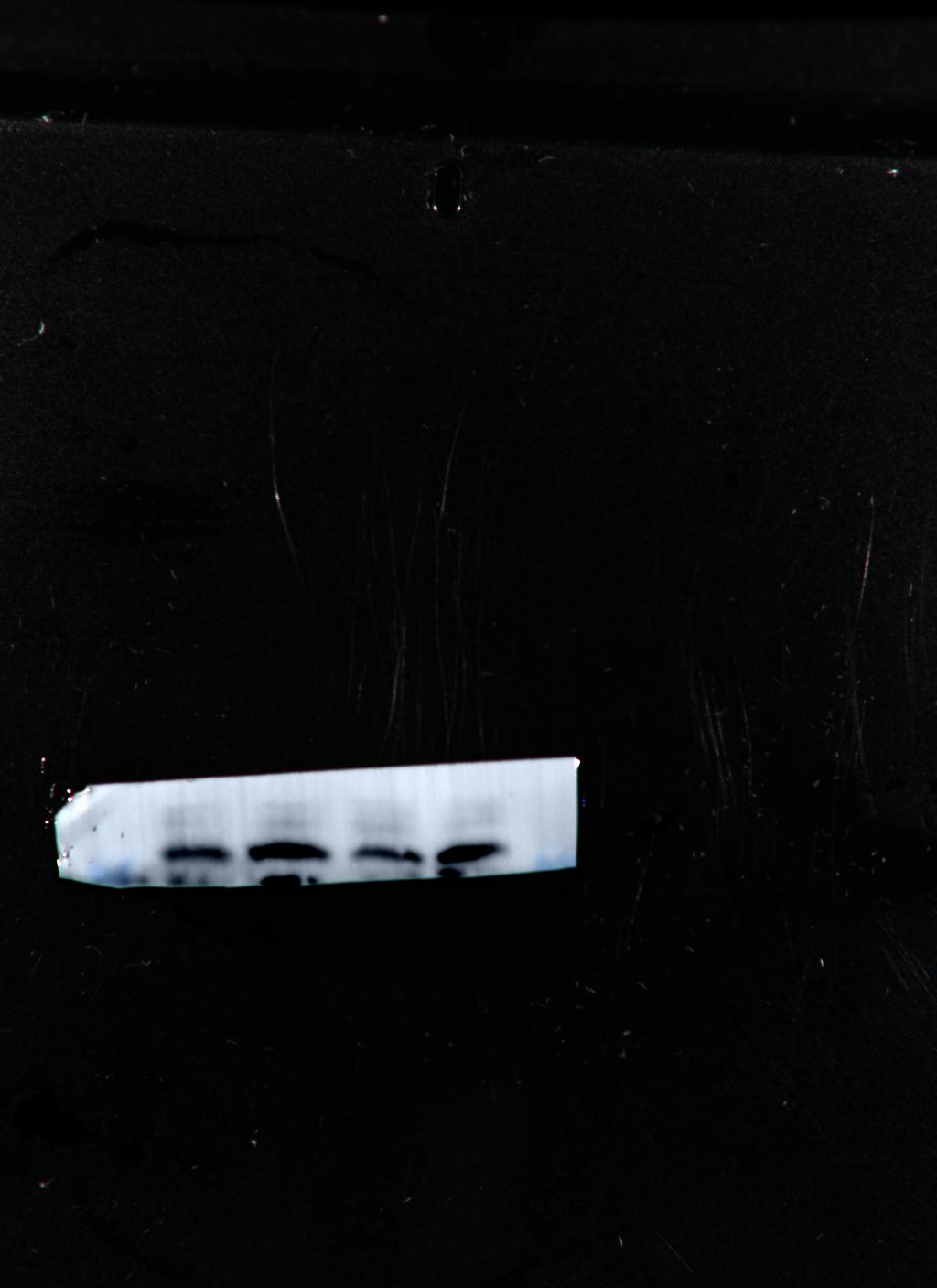

Supplement: Supplemental Information 2 [file peerj-10-14267-s002.zip › uncropped blots/figure3-uncropped blots/A-uncropped blots/p16-1.jpg]

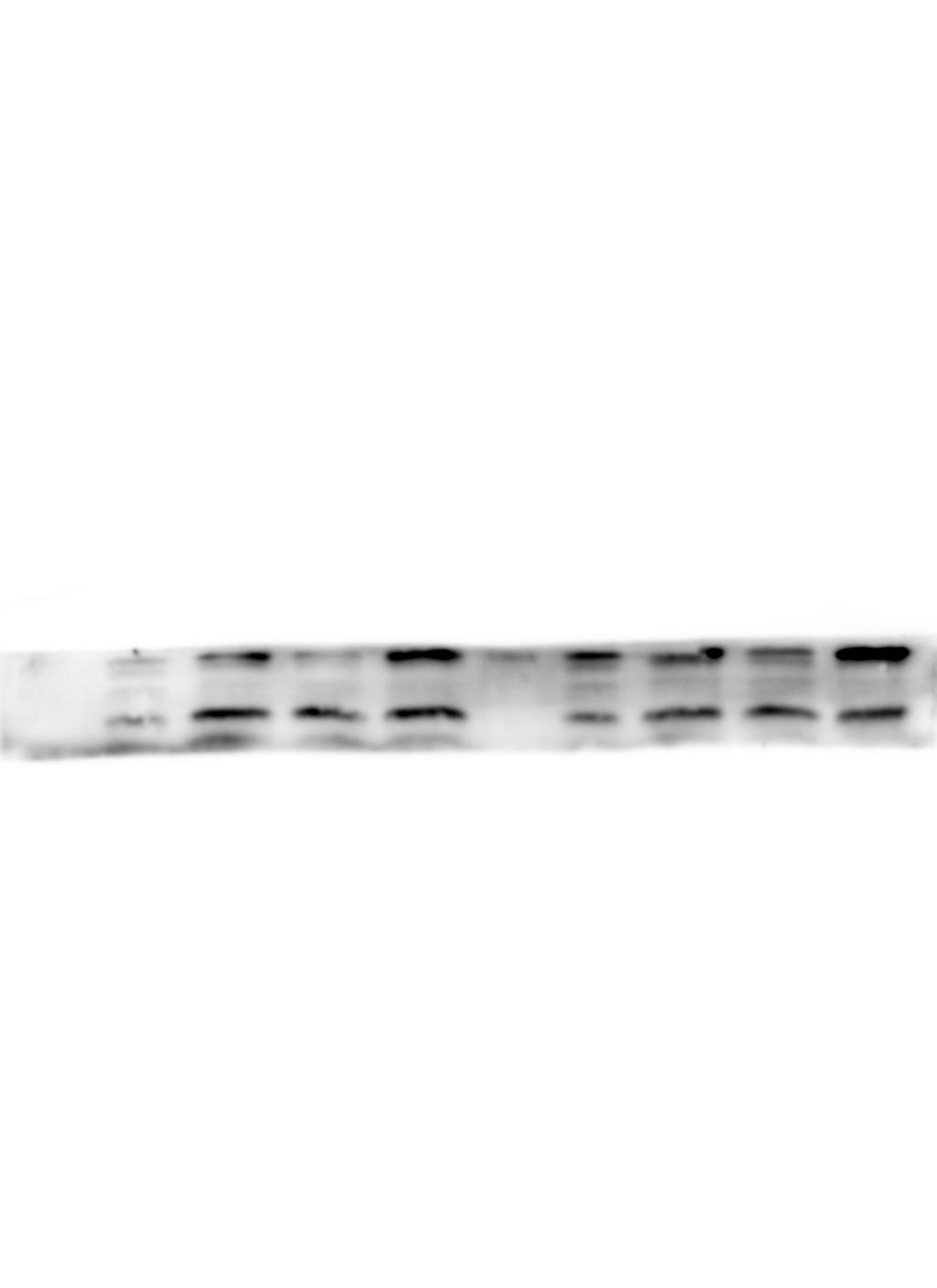

Supplement: Supplemental Information 2 [file peerj-10-14267-s002.zip › uncropped blots/figure3-uncropped blots/A-uncropped blots/p16-2.3.jpg]

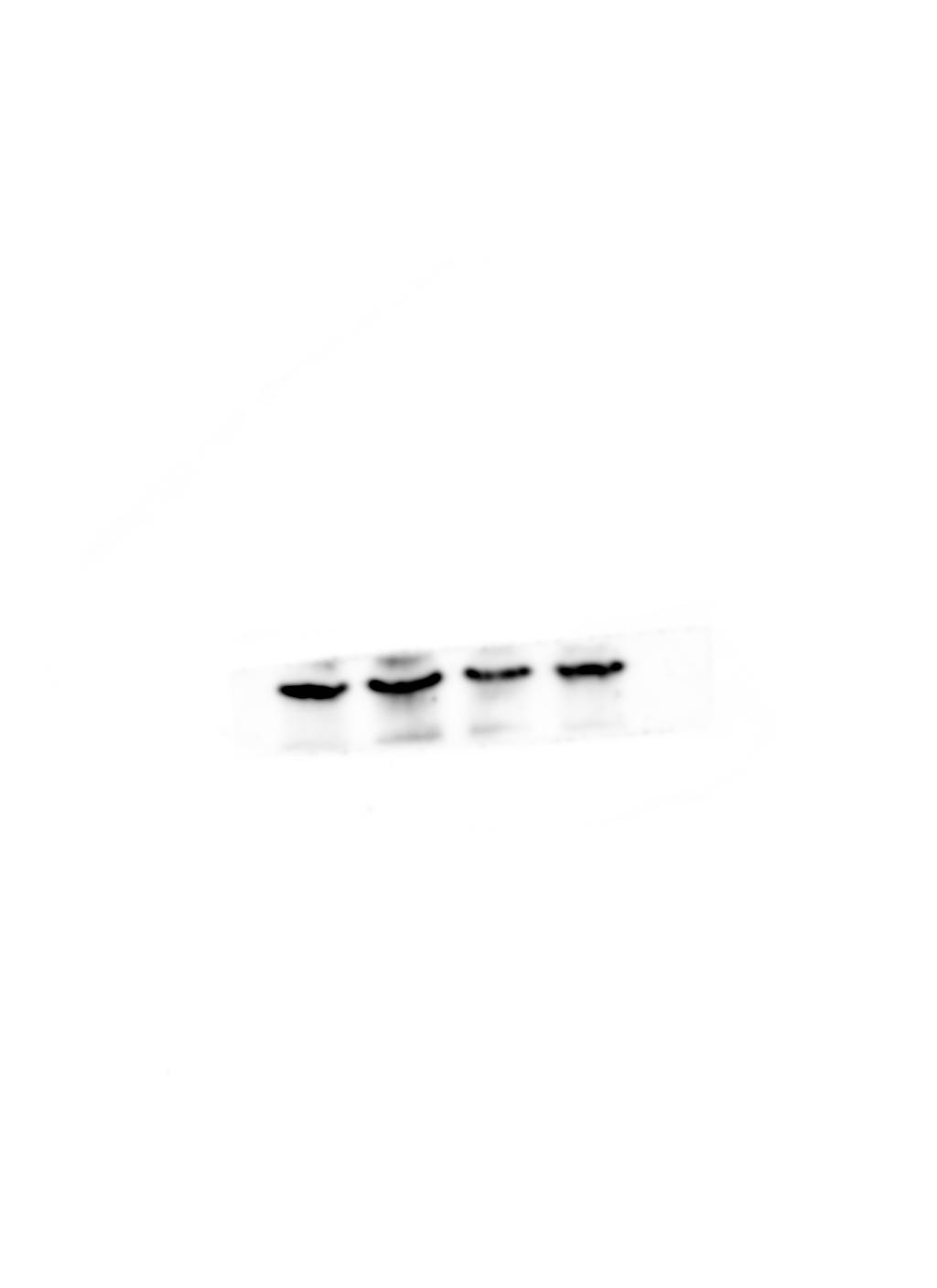

Supplement: Supplemental Information 2 [file peerj-10-14267-s002.zip › uncropped blots/figure3-uncropped blots/A-uncropped blots/p21-1.jpg]

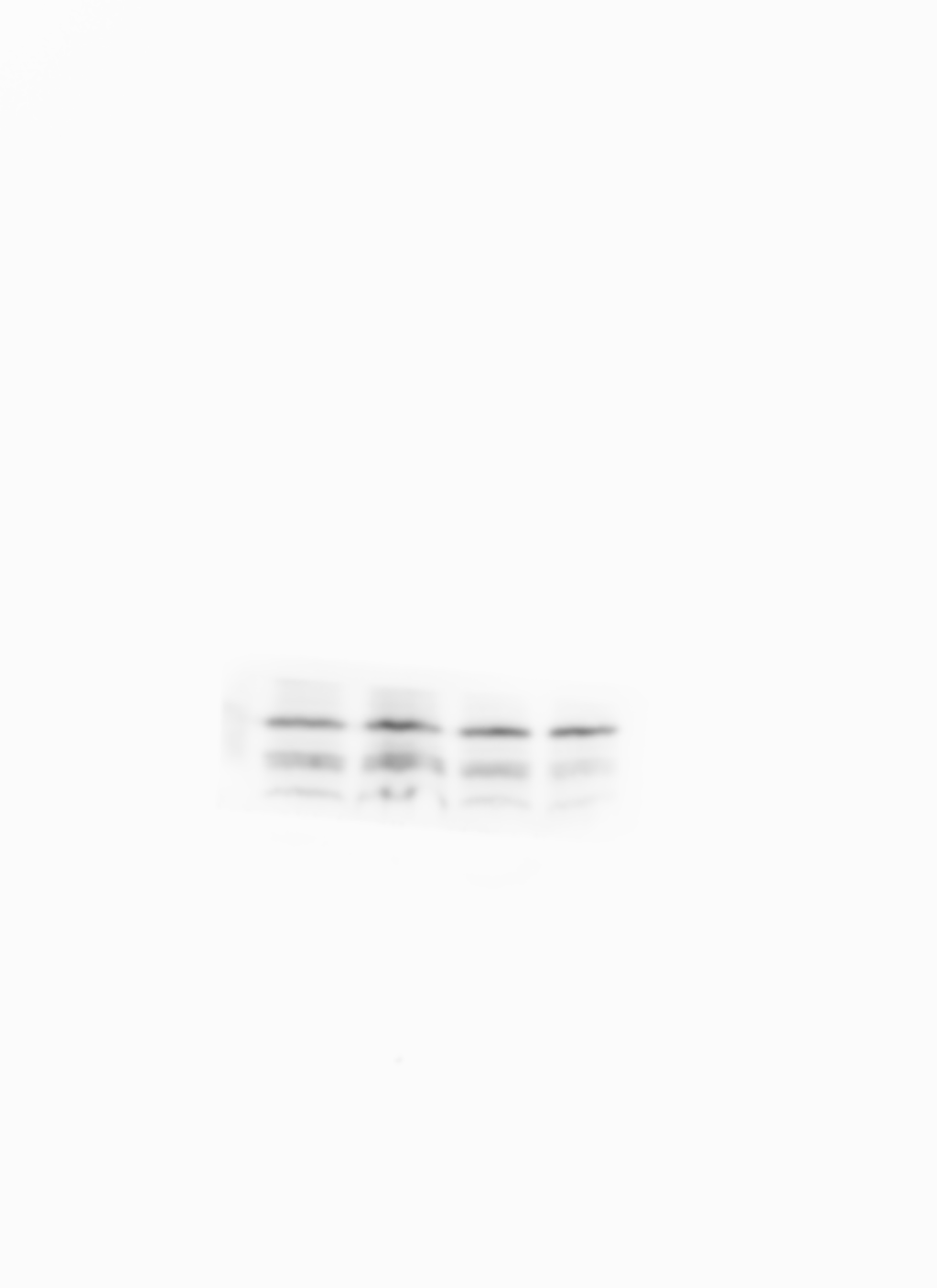

Supplement: Supplemental Information 2 [file peerj-10-14267-s002.zip › uncropped blots/figure3-uncropped blots/A-uncropped blots/p21-2.tif]

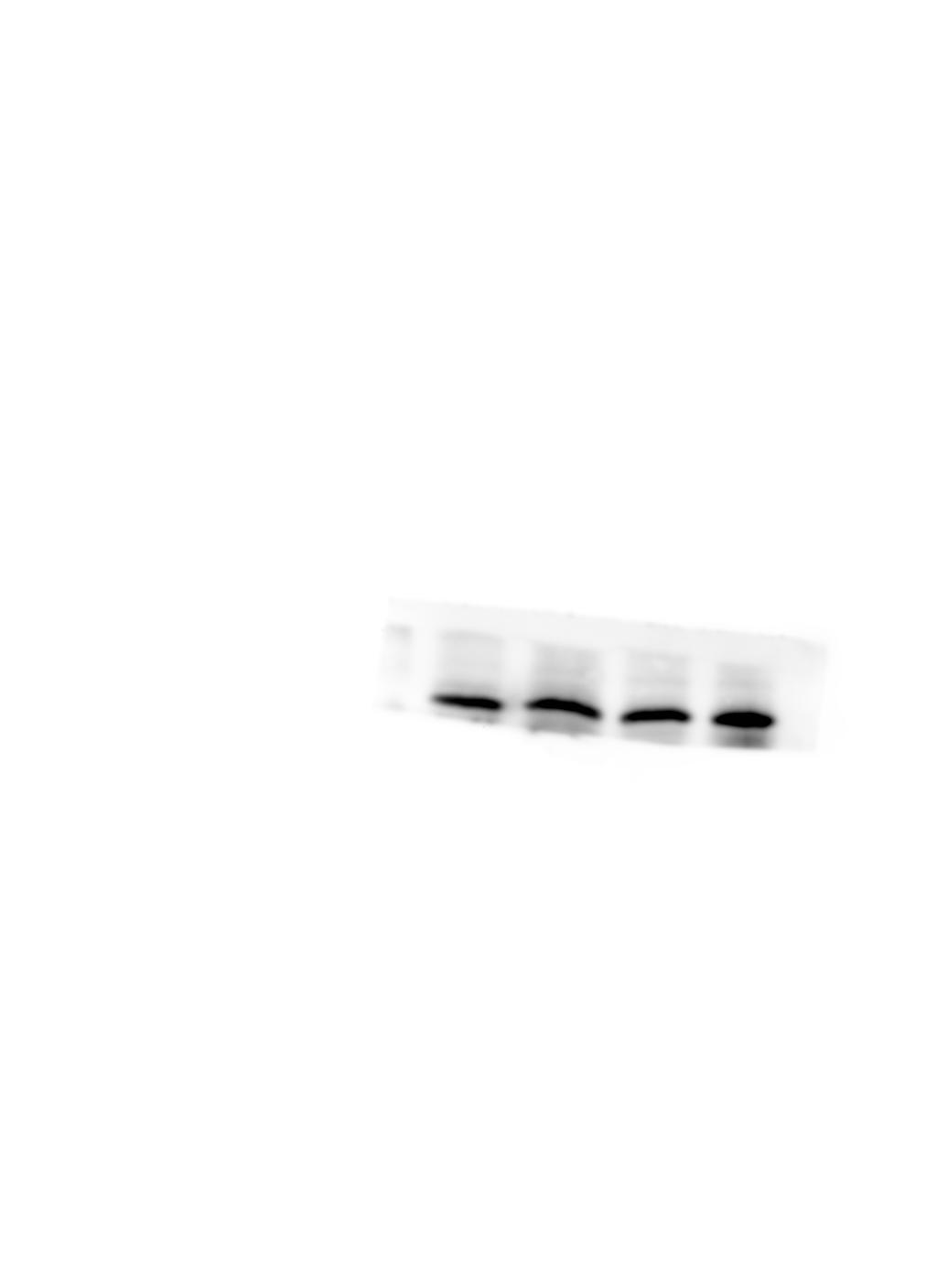

Supplement: Supplemental Information 2 [file peerj-10-14267-s002.zip › uncropped blots/figure3-uncropped blots/A-uncropped blots/p21-3.jpg]

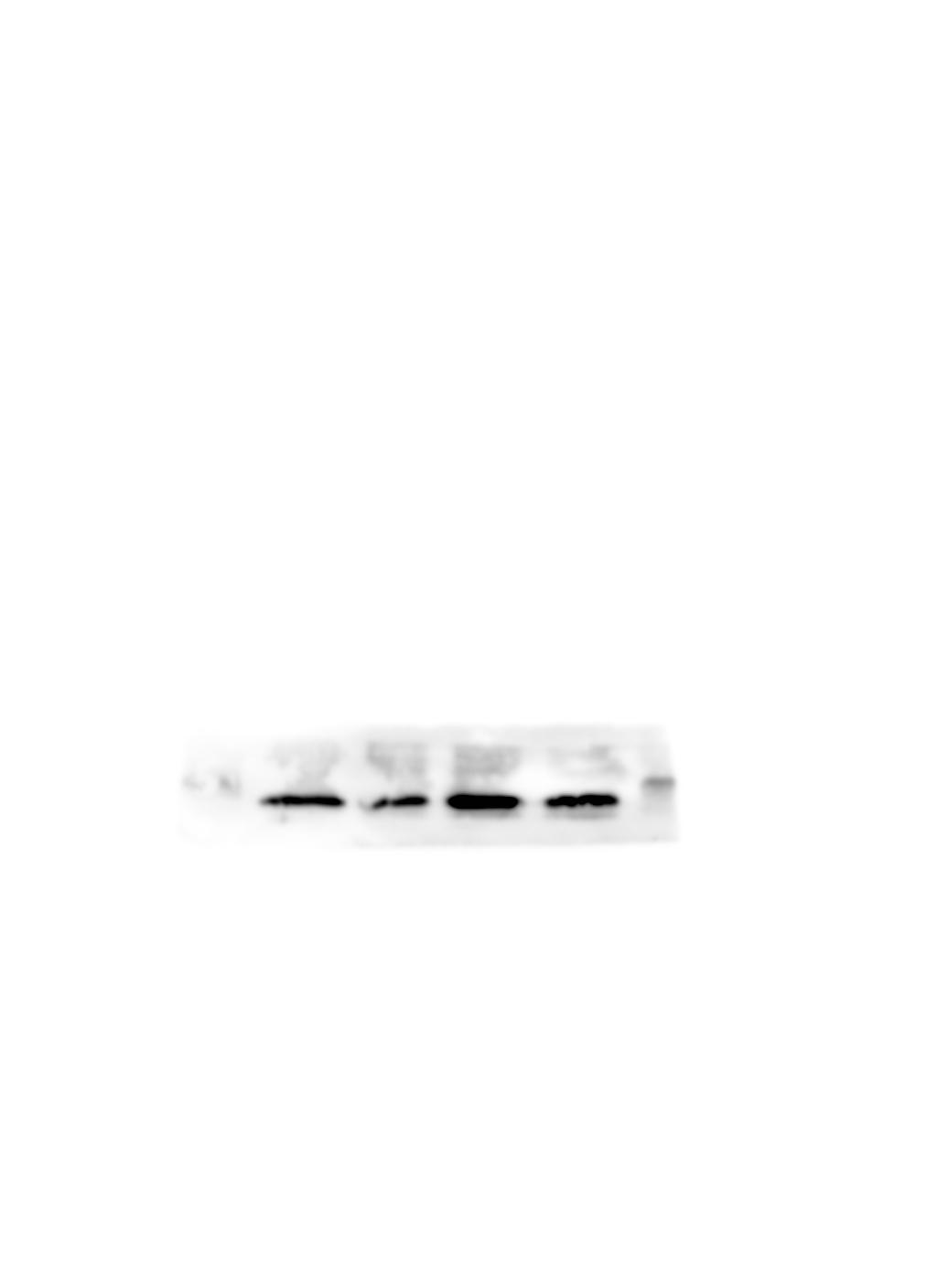

Supplement: Supplemental Information 2 [file peerj-10-14267-s002.zip › uncropped blots/figure3-uncropped blots/A-uncropped blots/p62-1.jpg]

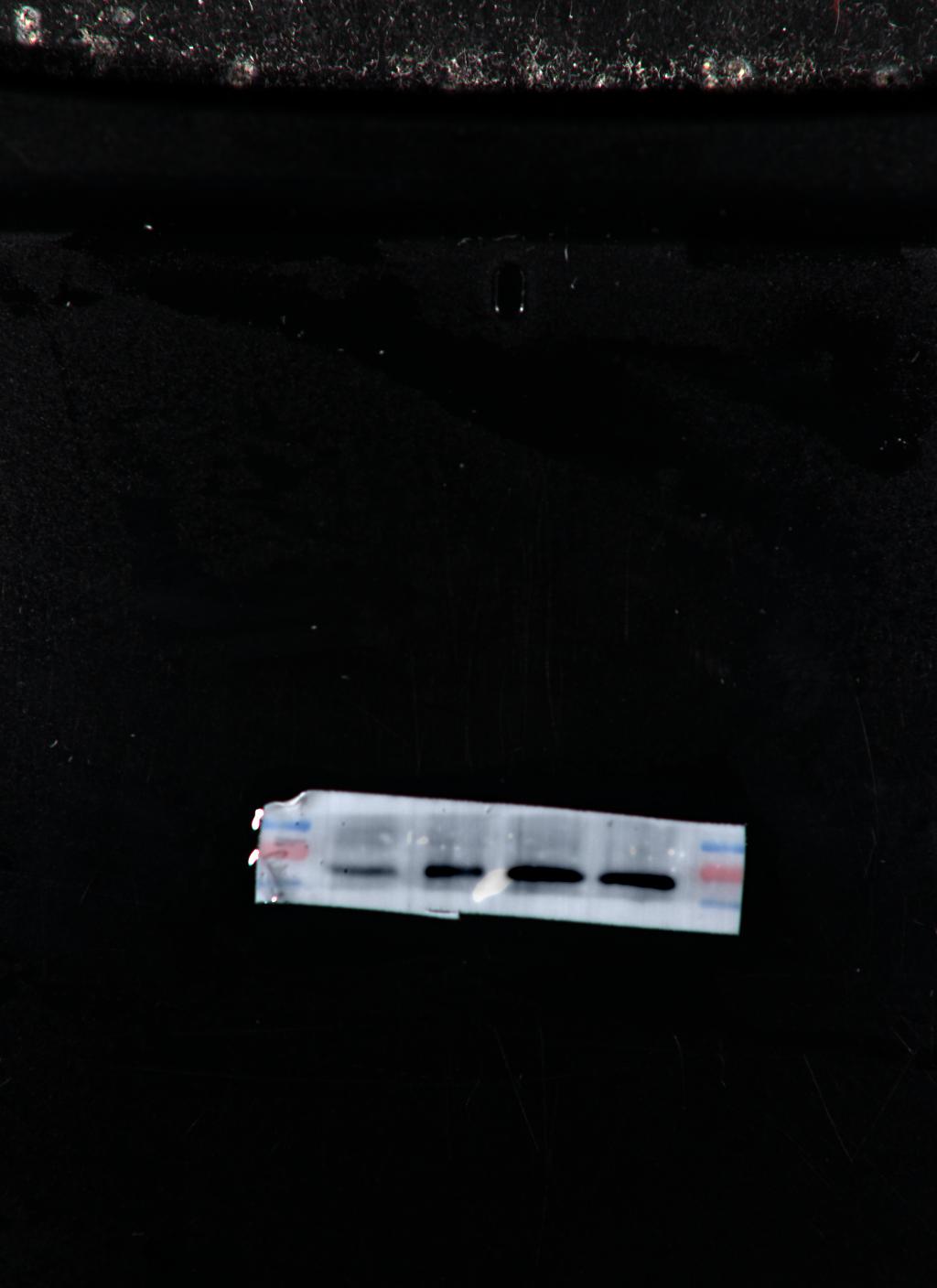

Supplement: Supplemental Information 2 [file peerj-10-14267-s002.zip › uncropped blots/figure3-uncropped blots/A-uncropped blots/p62-2.jpg]

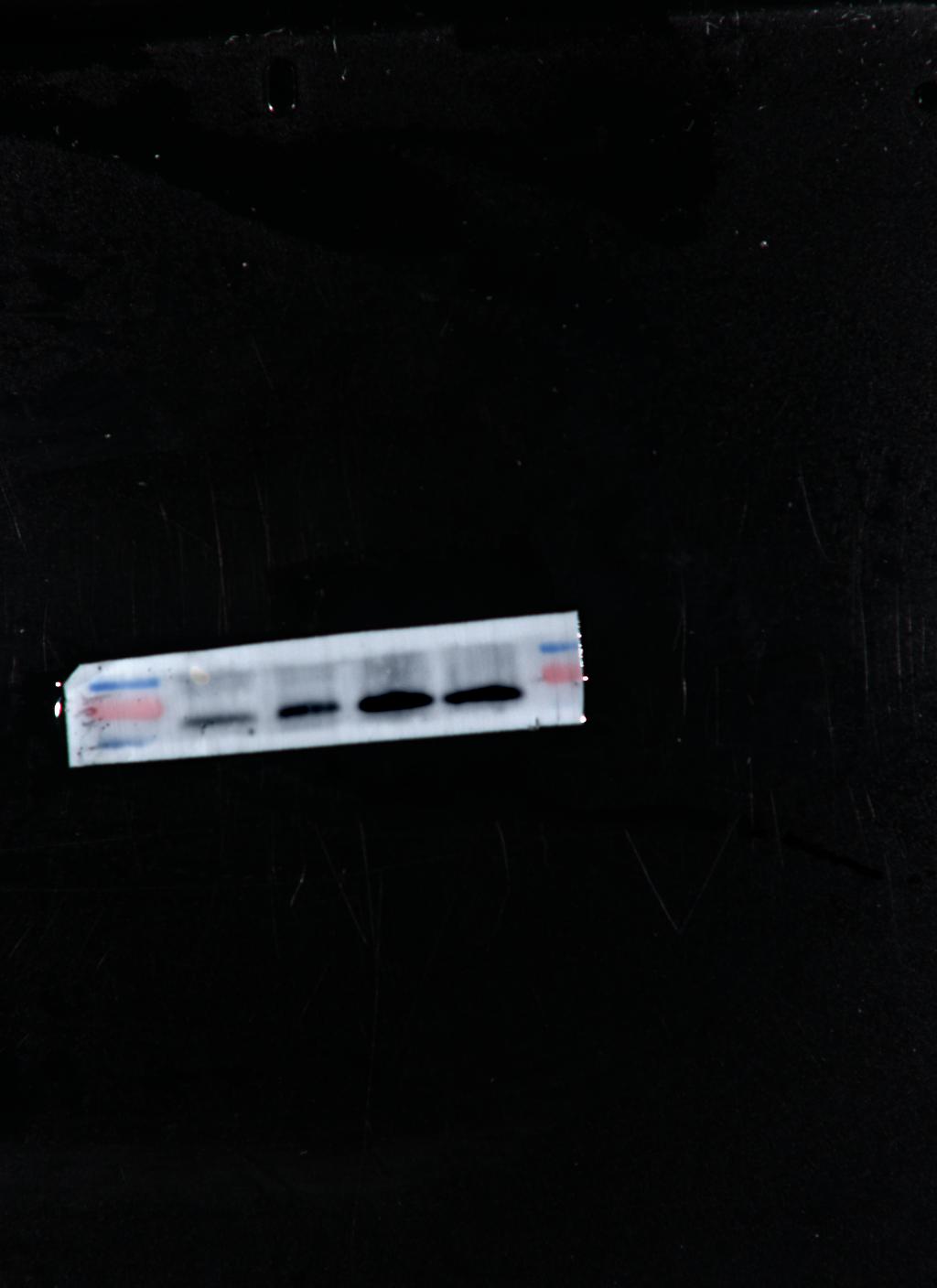

Supplement: Supplemental Information 2 [file peerj-10-14267-s002.zip › uncropped blots/figure3-uncropped blots/A-uncropped blots/p62-3.jpg]

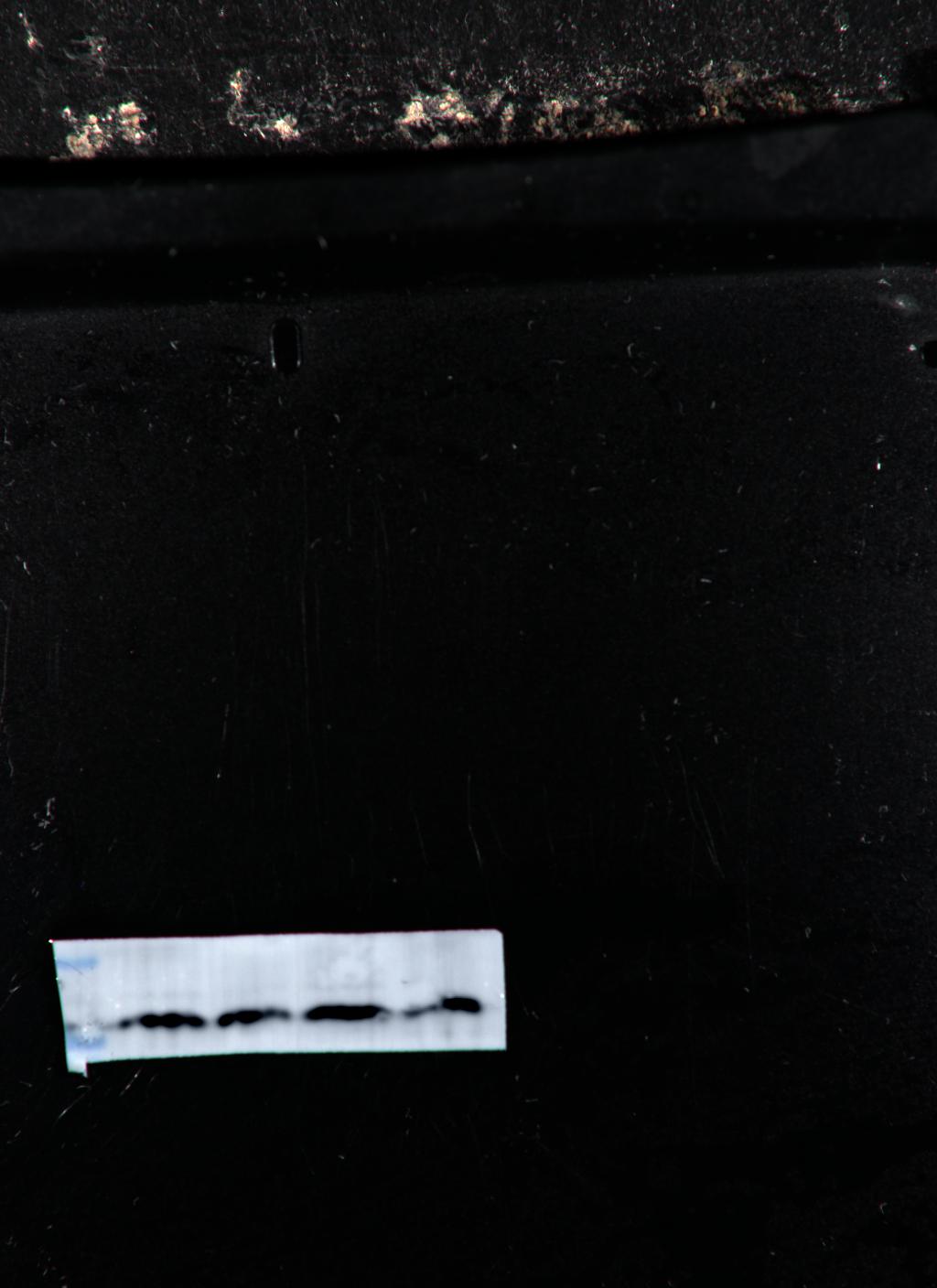

Supplement: Supplemental Information 2 [file peerj-10-14267-s002.zip › uncropped blots/figure3-uncropped blots/A-uncropped blots/PIN1-1.jpg]

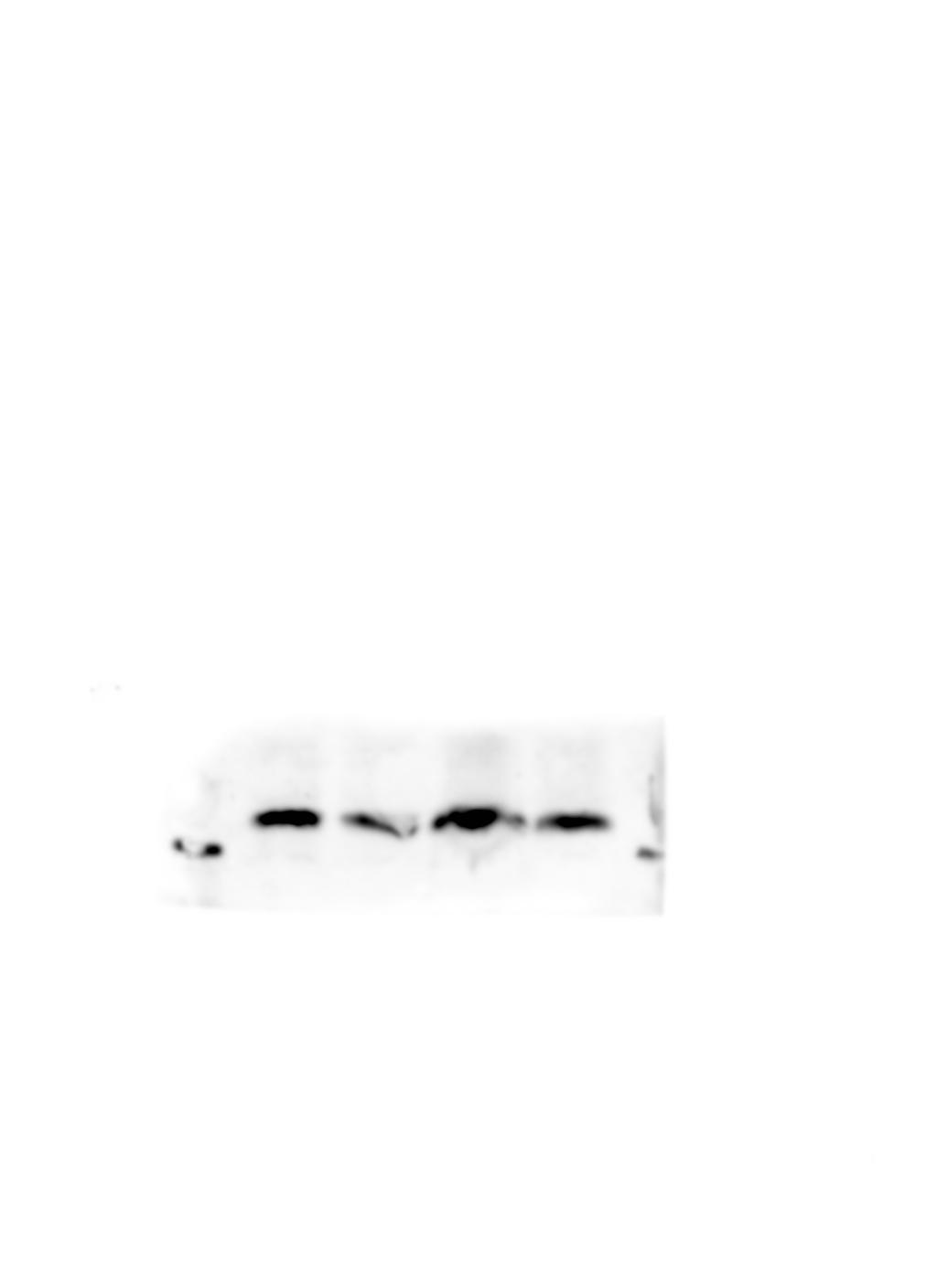

Supplement: Supplemental Information 2 [file peerj-10-14267-s002.zip › uncropped blots/figure3-uncropped blots/A-uncropped blots/PIN1-2.jpg]

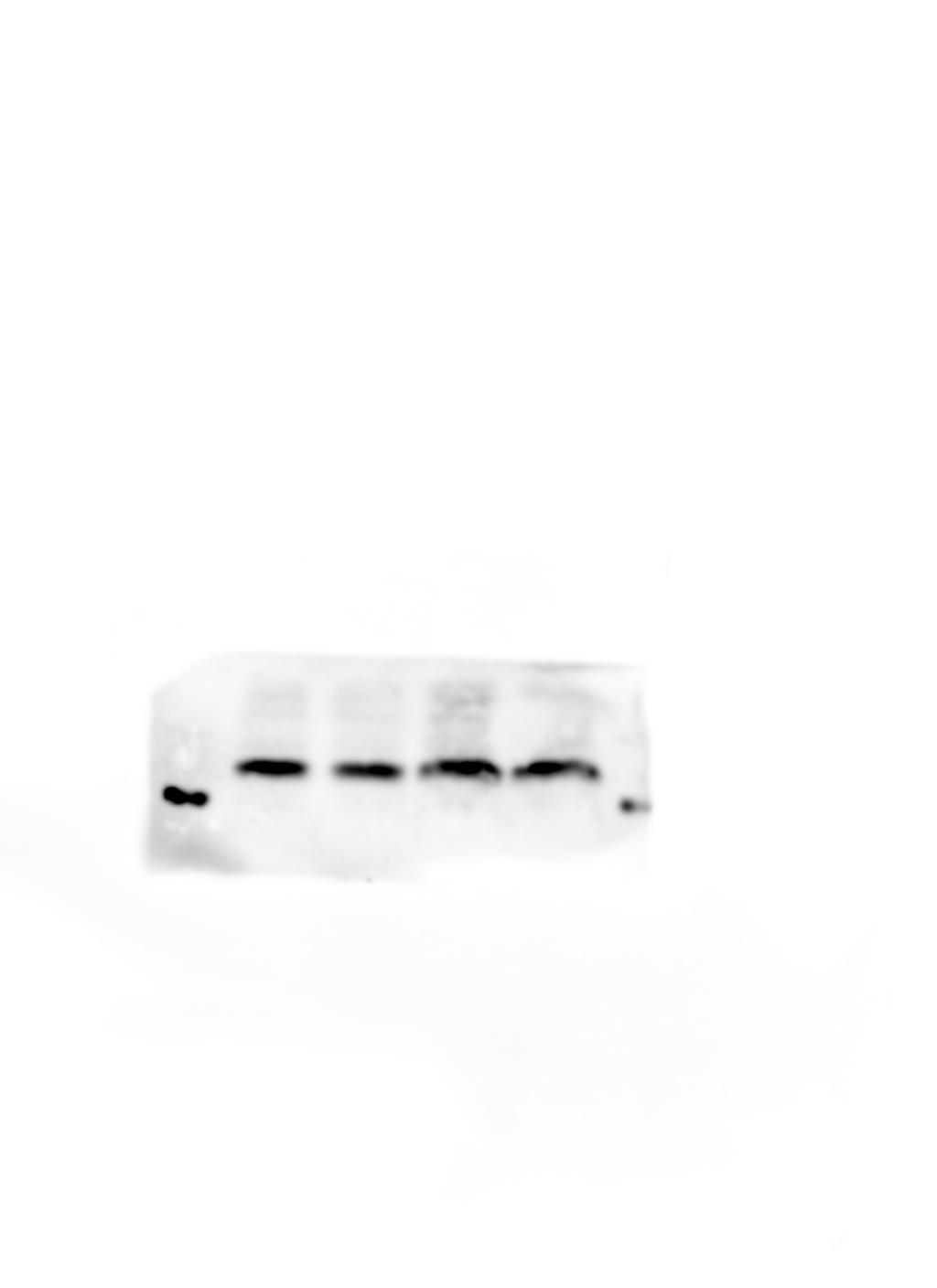

Supplement: Supplemental Information 2 [file peerj-10-14267-s002.zip › uncropped blots/figure3-uncropped blots/A-uncropped blots/PIN1-3.jpg]

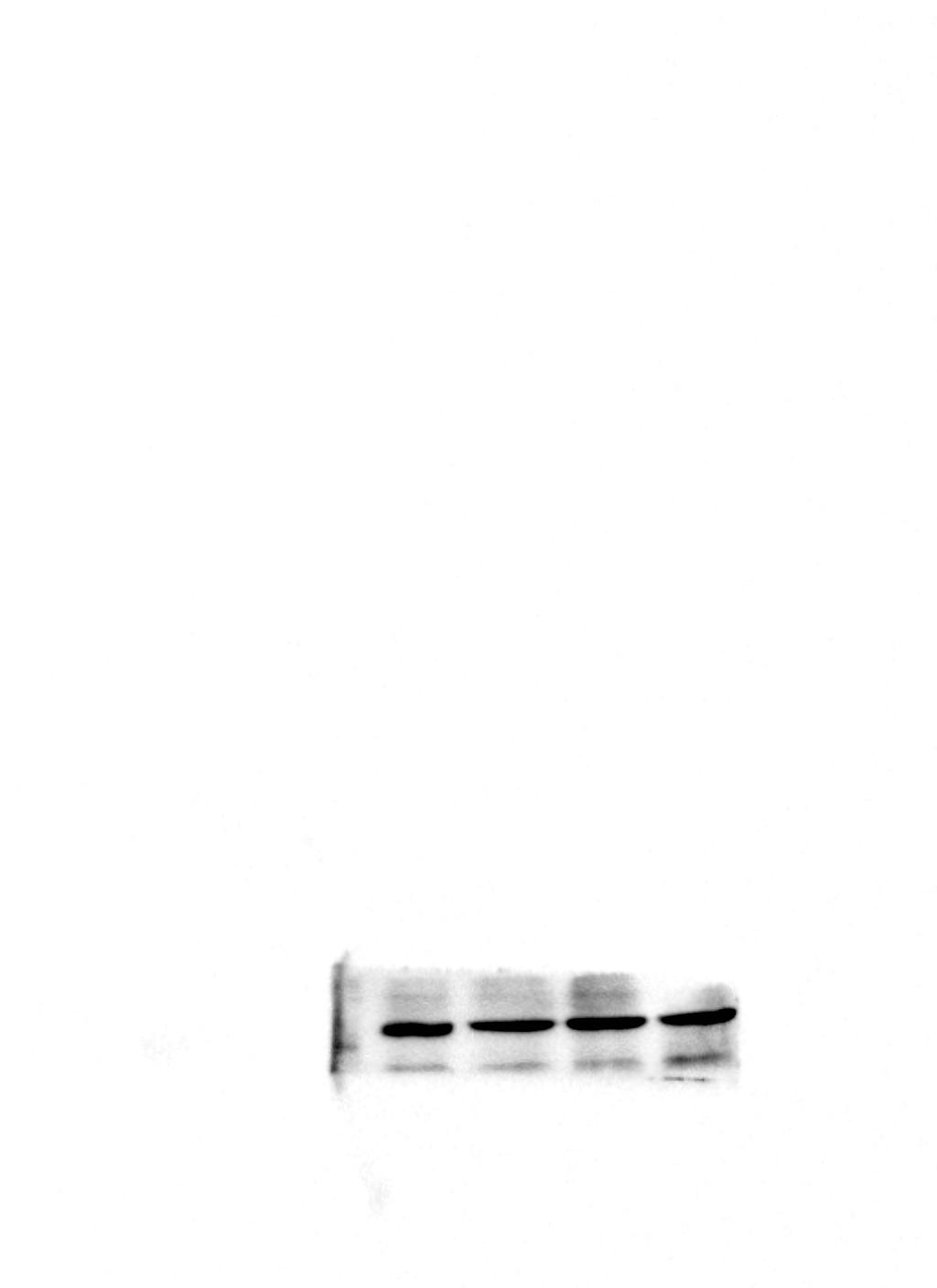

Supplement: Supplemental Information 2 [file peerj-10-14267-s002.zip › uncropped blots/figure3-uncropped blots/B-uncropped blots/actin-1.jpg]

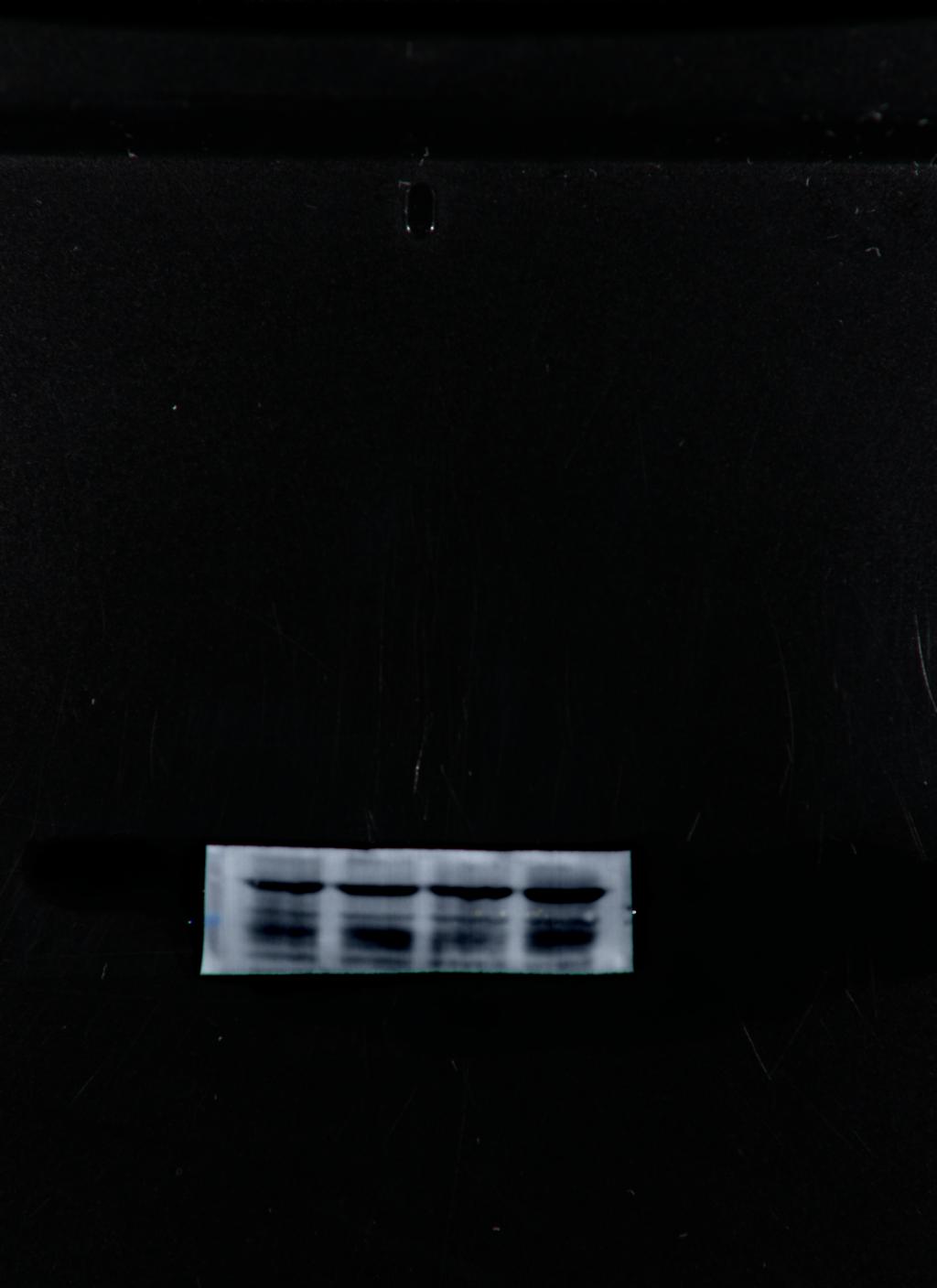

Supplement: Supplemental Information 2 [file peerj-10-14267-s002.zip › uncropped blots/figure3-uncropped blots/B-uncropped blots/actin-2.jpg]

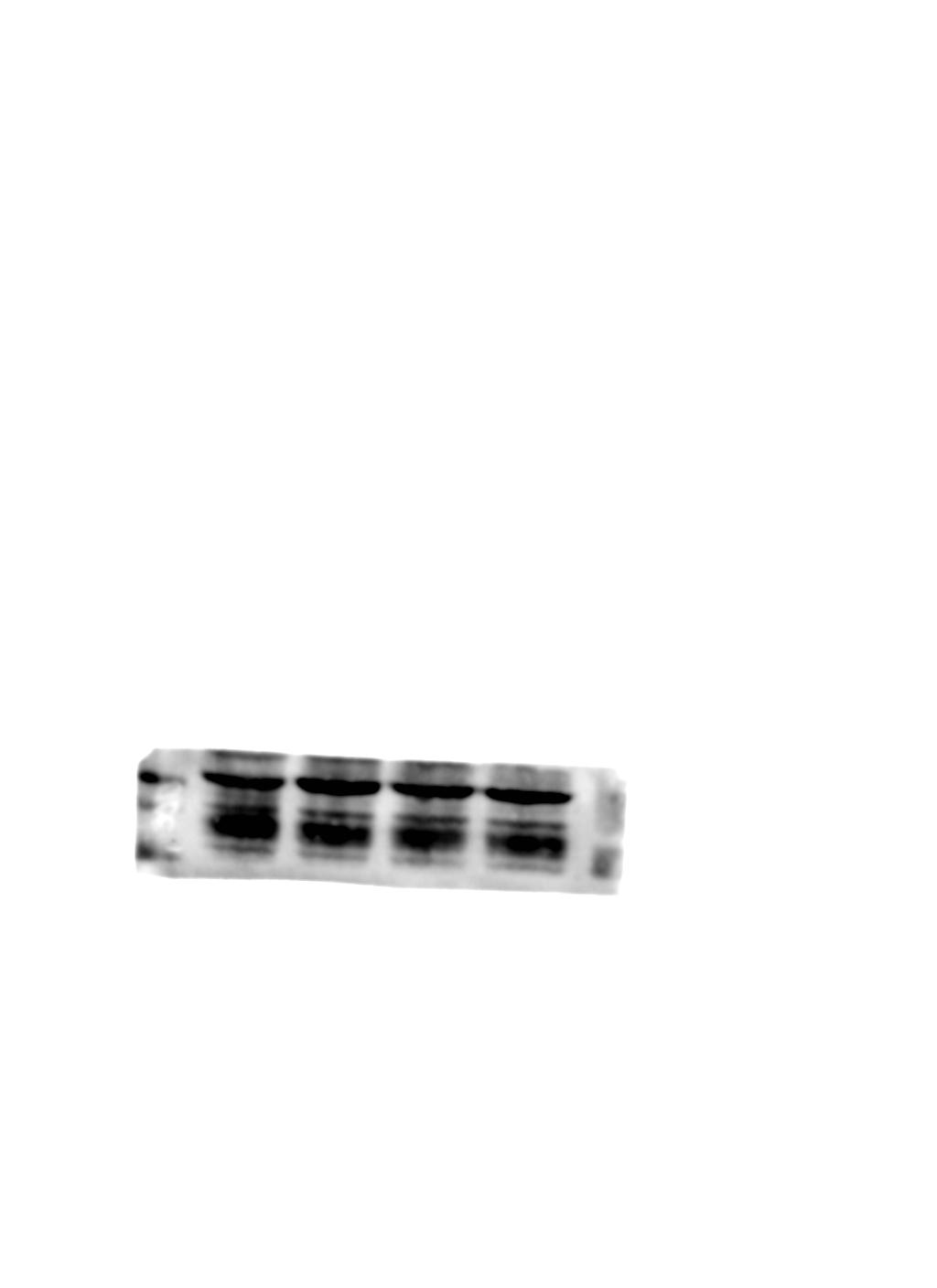

Supplement: Supplemental Information 2 [file peerj-10-14267-s002.zip › uncropped blots/figure3-uncropped blots/B-uncropped blots/actin-3.jpg]

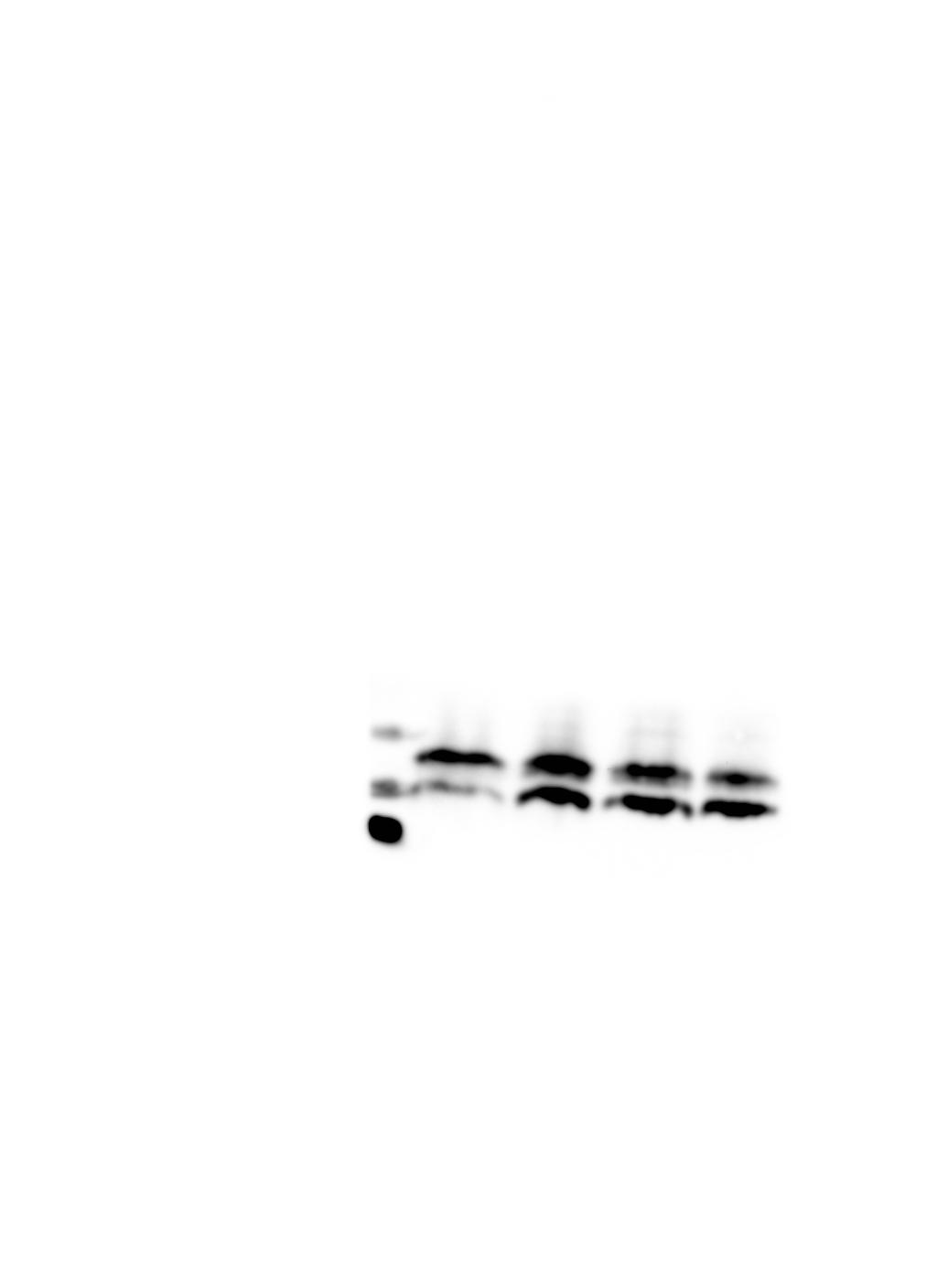

Supplement: Supplemental Information 2 [file peerj-10-14267-s002.zip › uncropped blots/figure3-uncropped blots/B-uncropped blots/LC3-1.jpg]

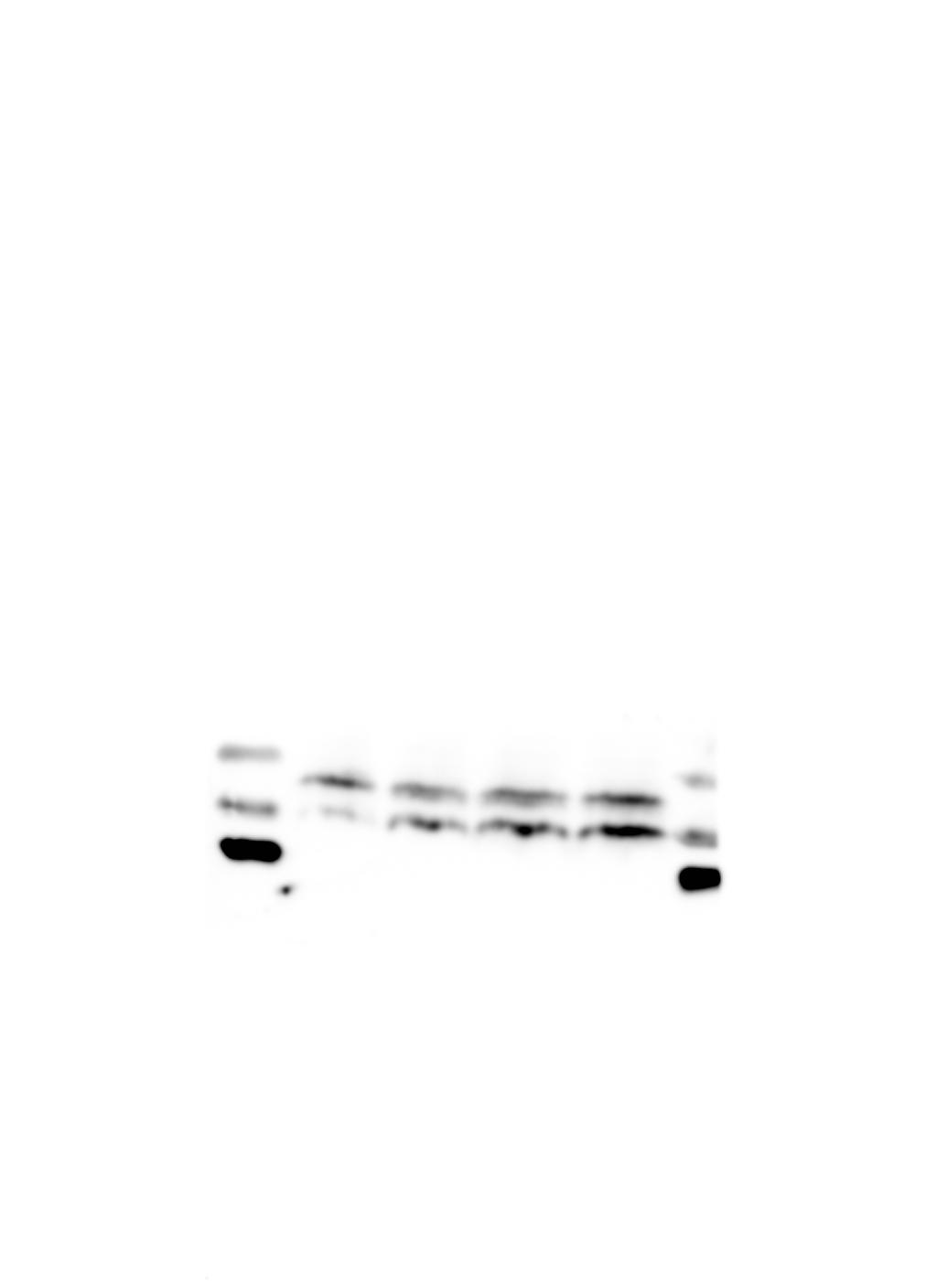

Supplement: Supplemental Information 2 [file peerj-10-14267-s002.zip › uncropped blots/figure3-uncropped blots/B-uncropped blots/LC3-2.jpg]

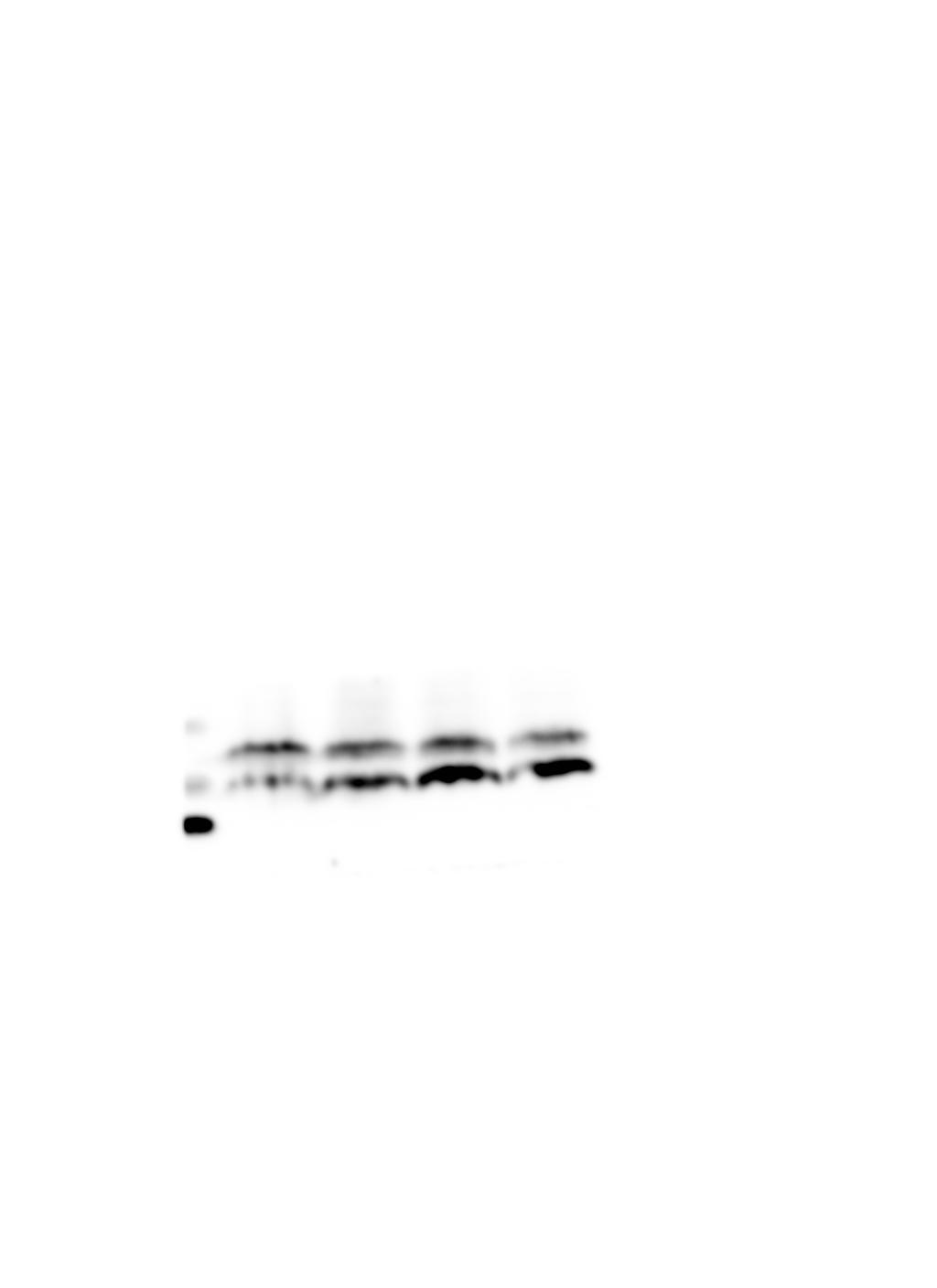

Supplement: Supplemental Information 2 [file peerj-10-14267-s002.zip › uncropped blots/figure3-uncropped blots/B-uncropped blots/LC3-3.jpg]

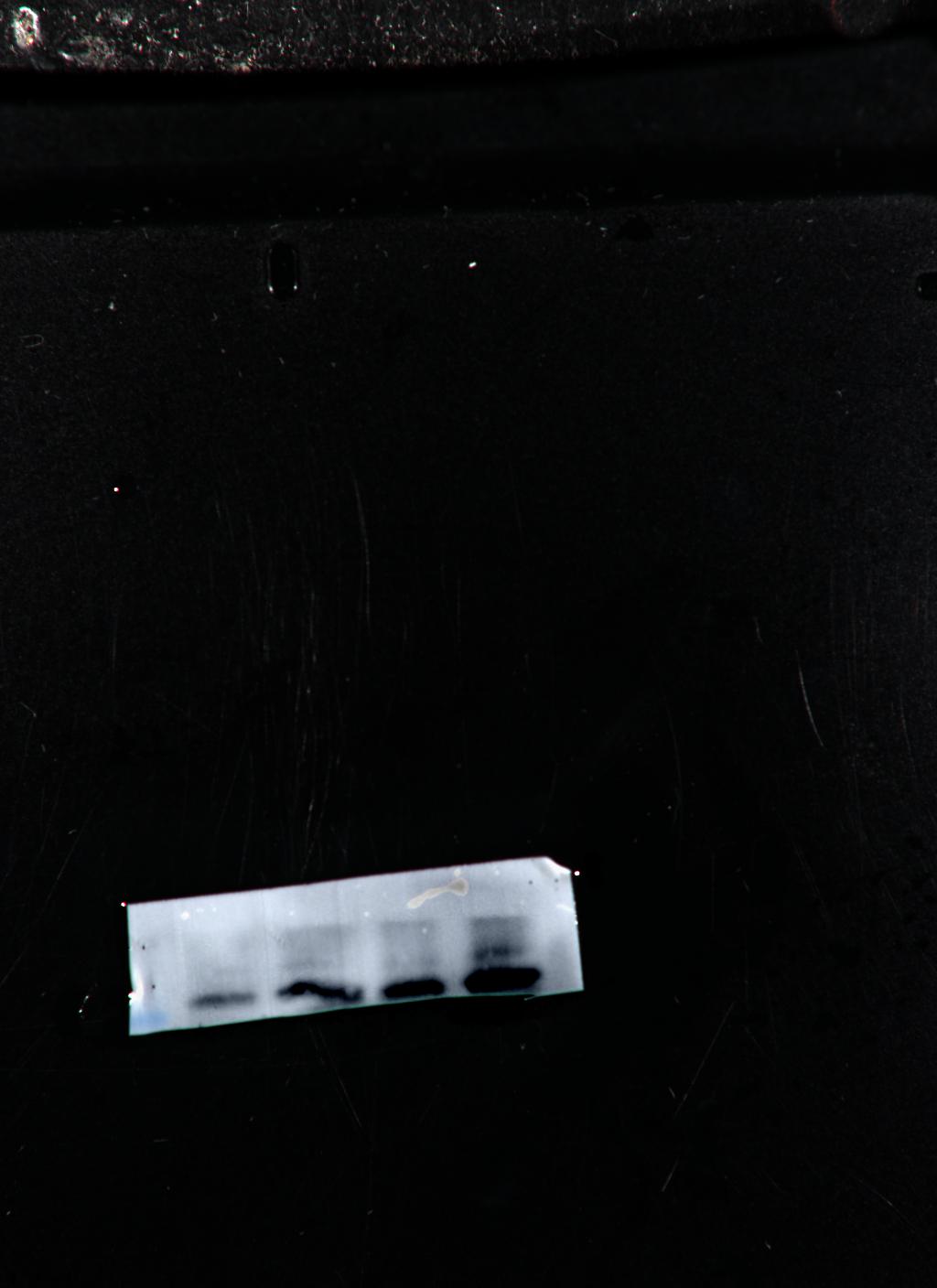

Supplement: Supplemental Information 2 [file peerj-10-14267-s002.zip › uncropped blots/figure3-uncropped blots/B-uncropped blots/p16-1.jpg]

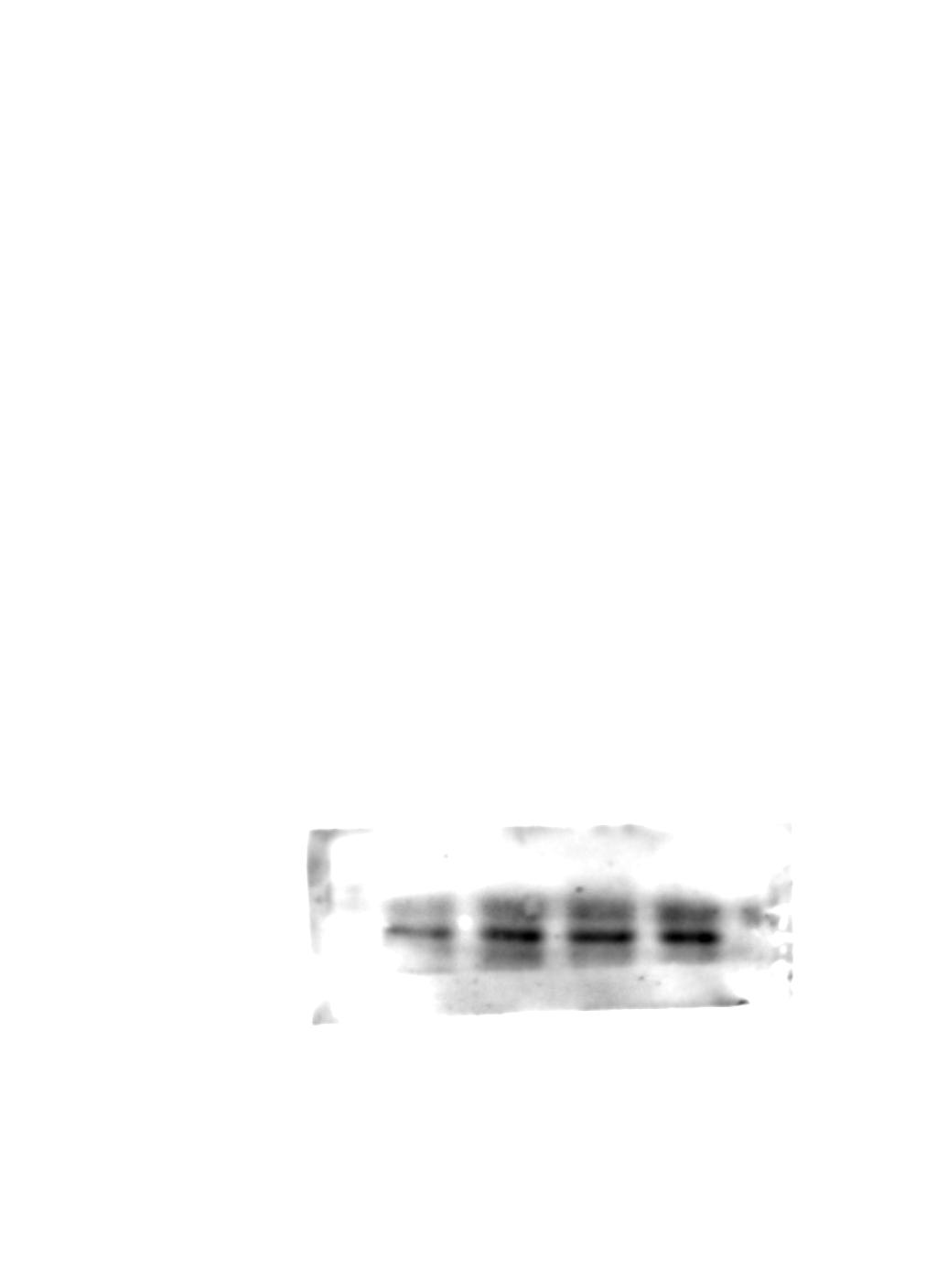

Supplement: Supplemental Information 2 [file peerj-10-14267-s002.zip › uncropped blots/figure3-uncropped blots/B-uncropped blots/p16-2.jpg]

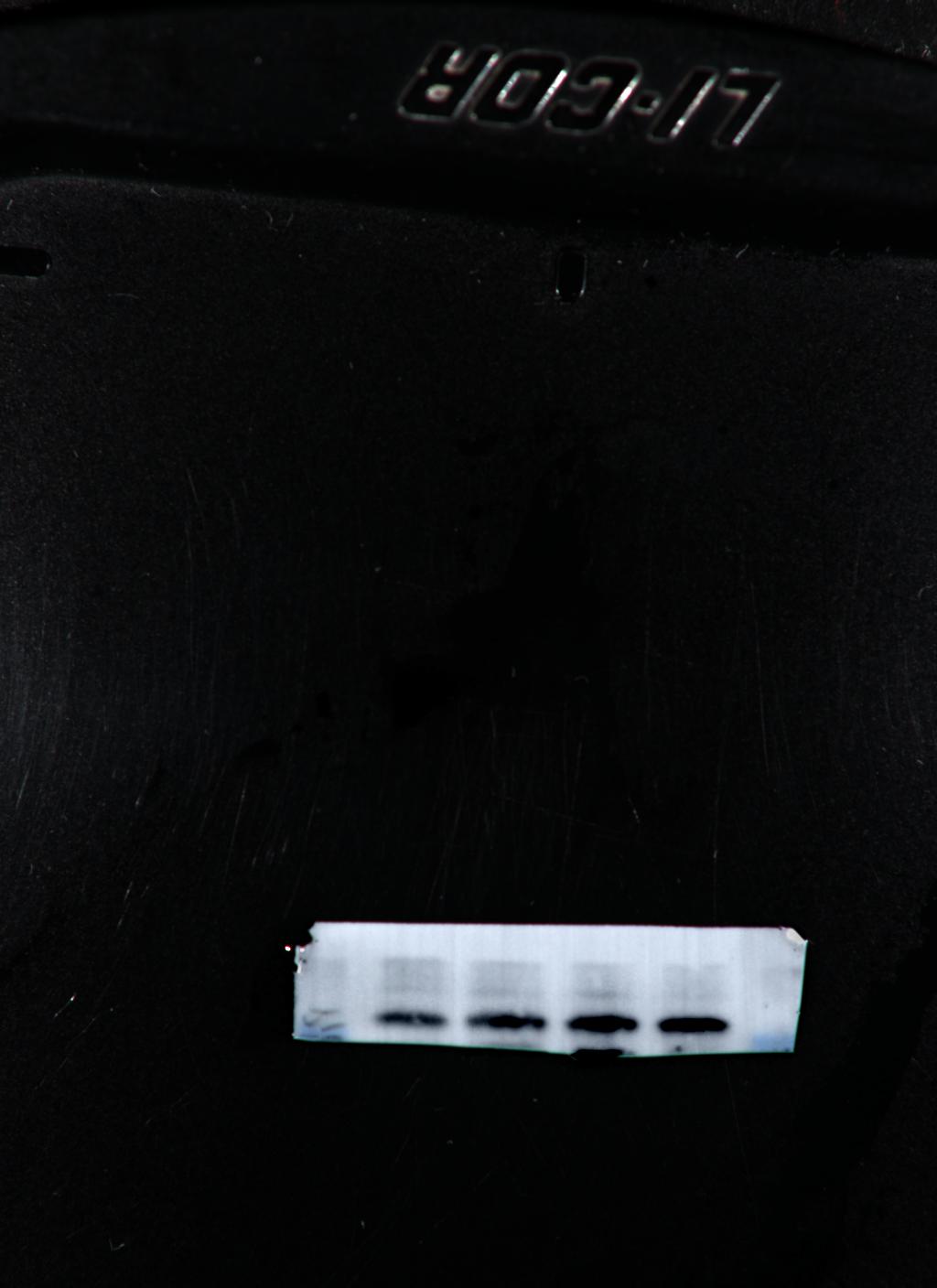

Supplement: Supplemental Information 2 [file peerj-10-14267-s002.zip › uncropped blots/figure3-uncropped blots/B-uncropped blots/p16-3.jpg]

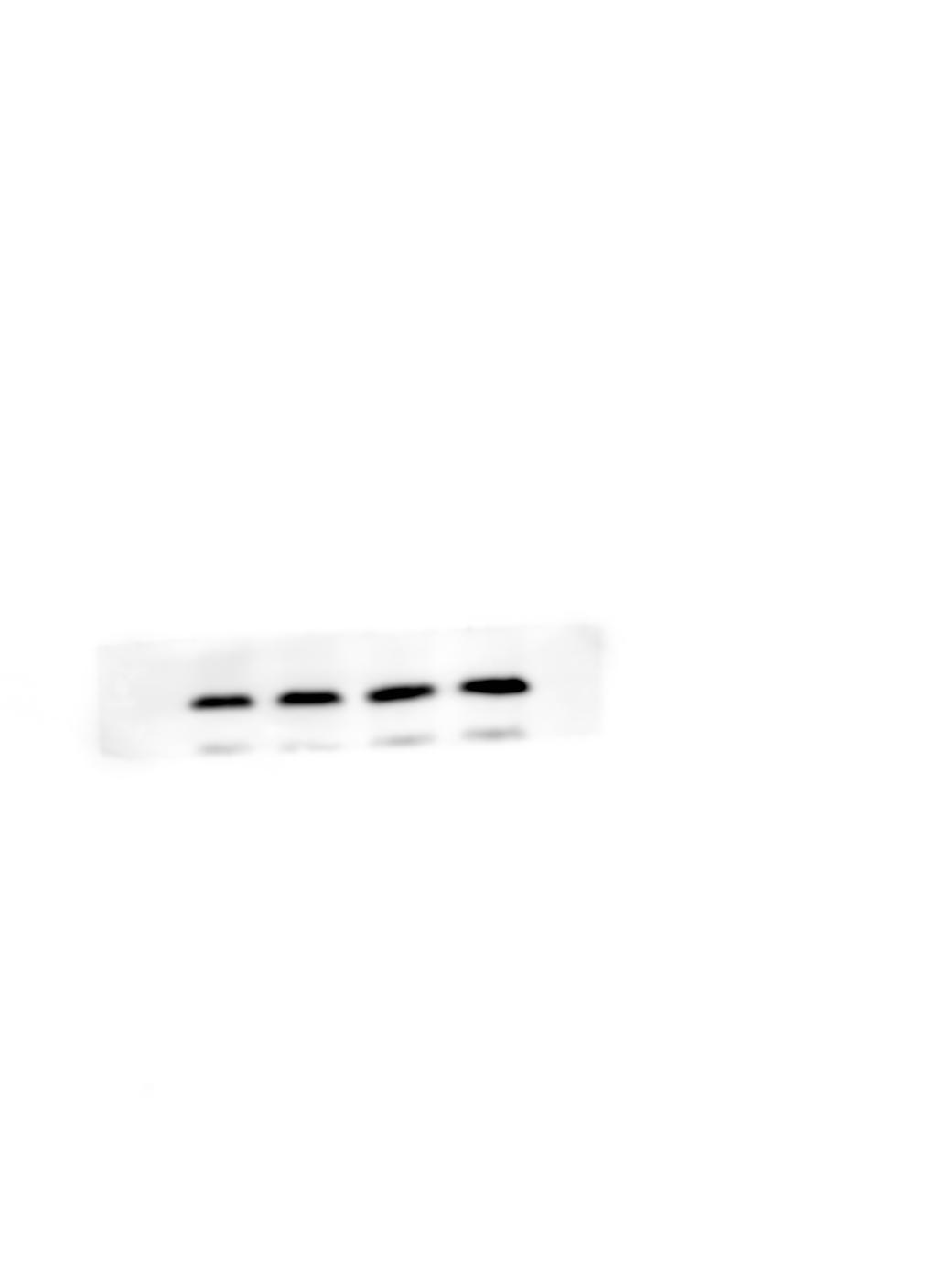

Supplement: Supplemental Information 2 [file peerj-10-14267-s002.zip › uncropped blots/figure3-uncropped blots/B-uncropped blots/p21-1.jpg]

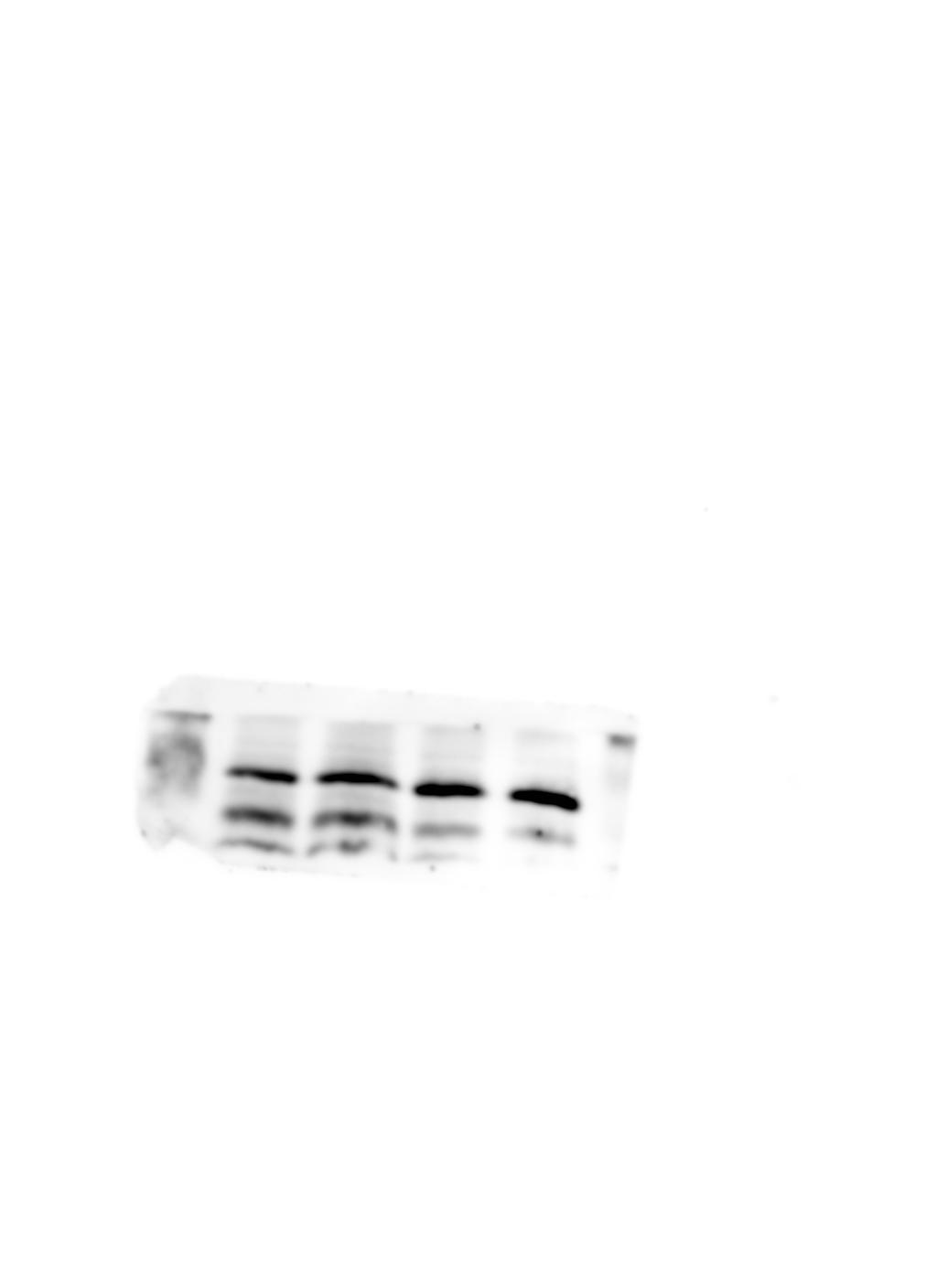

Supplement: Supplemental Information 2 [file peerj-10-14267-s002.zip › uncropped blots/figure3-uncropped blots/B-uncropped blots/p21-2.jpg]

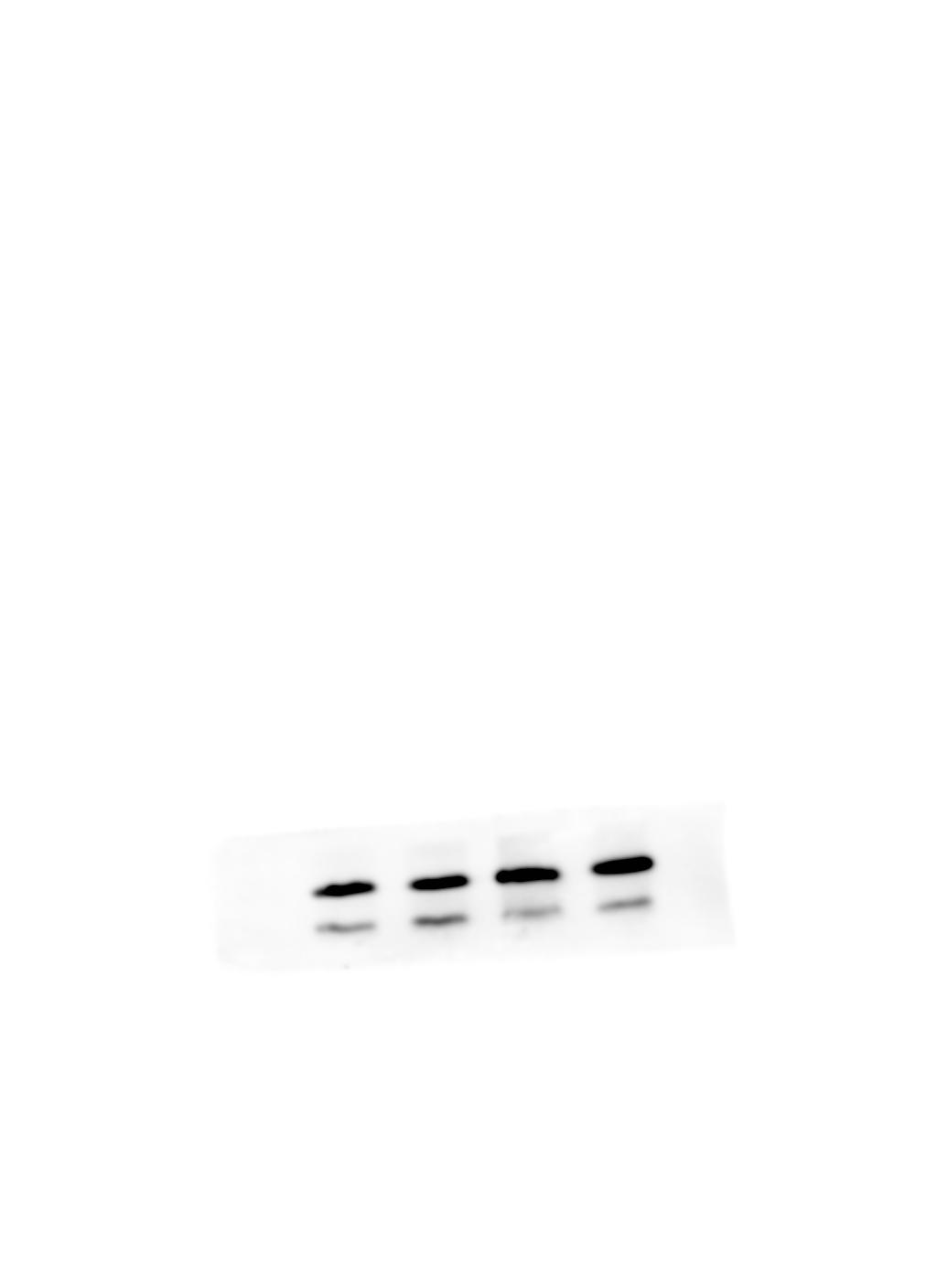

Supplement: Supplemental Information 2 [file peerj-10-14267-s002.zip › uncropped blots/figure3-uncropped blots/B-uncropped blots/p21-3.jpg]

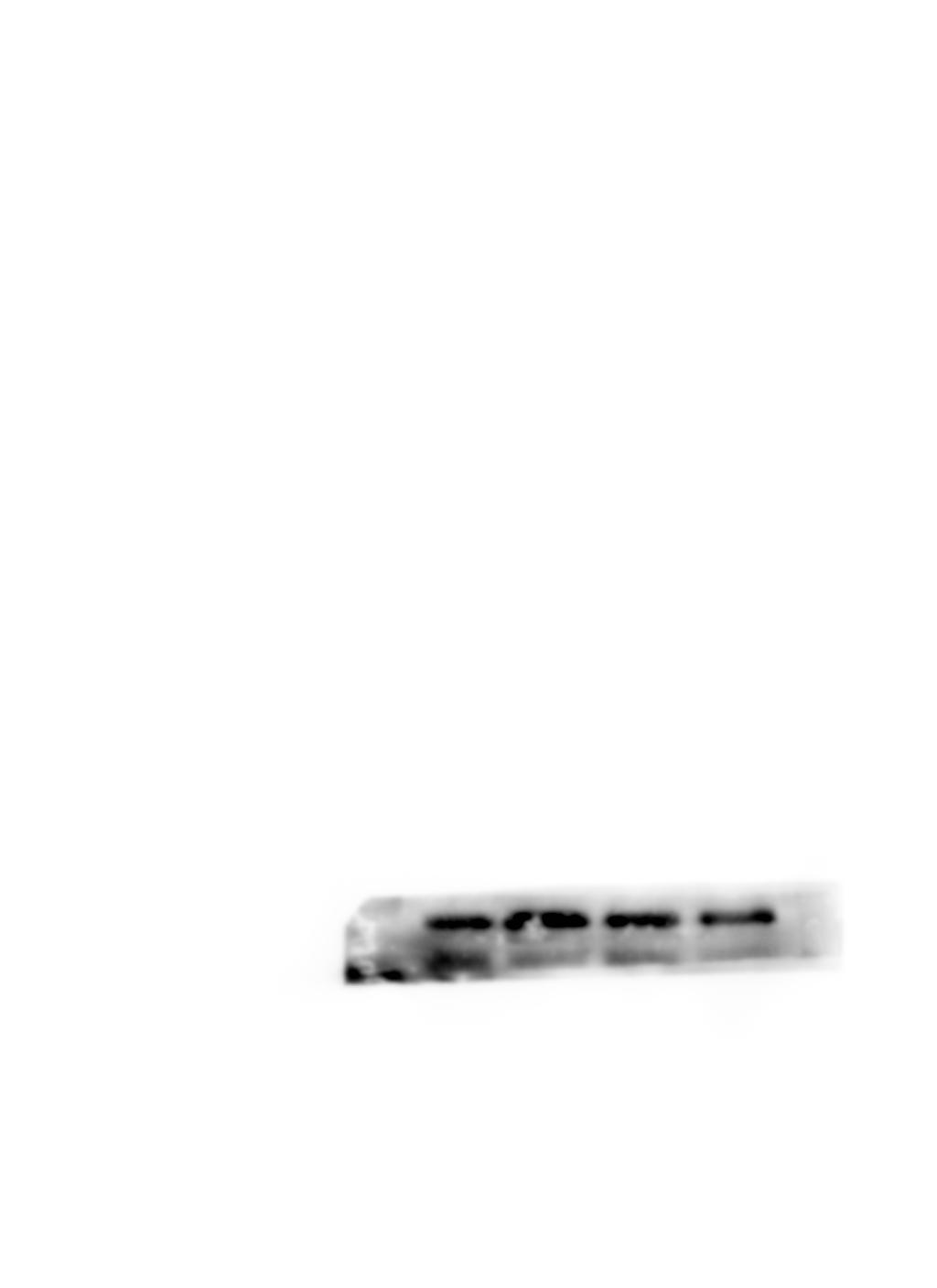

Supplement: Supplemental Information 2 [file peerj-10-14267-s002.zip › uncropped blots/figure3-uncropped blots/B-uncropped blots/p62-1.jpg]

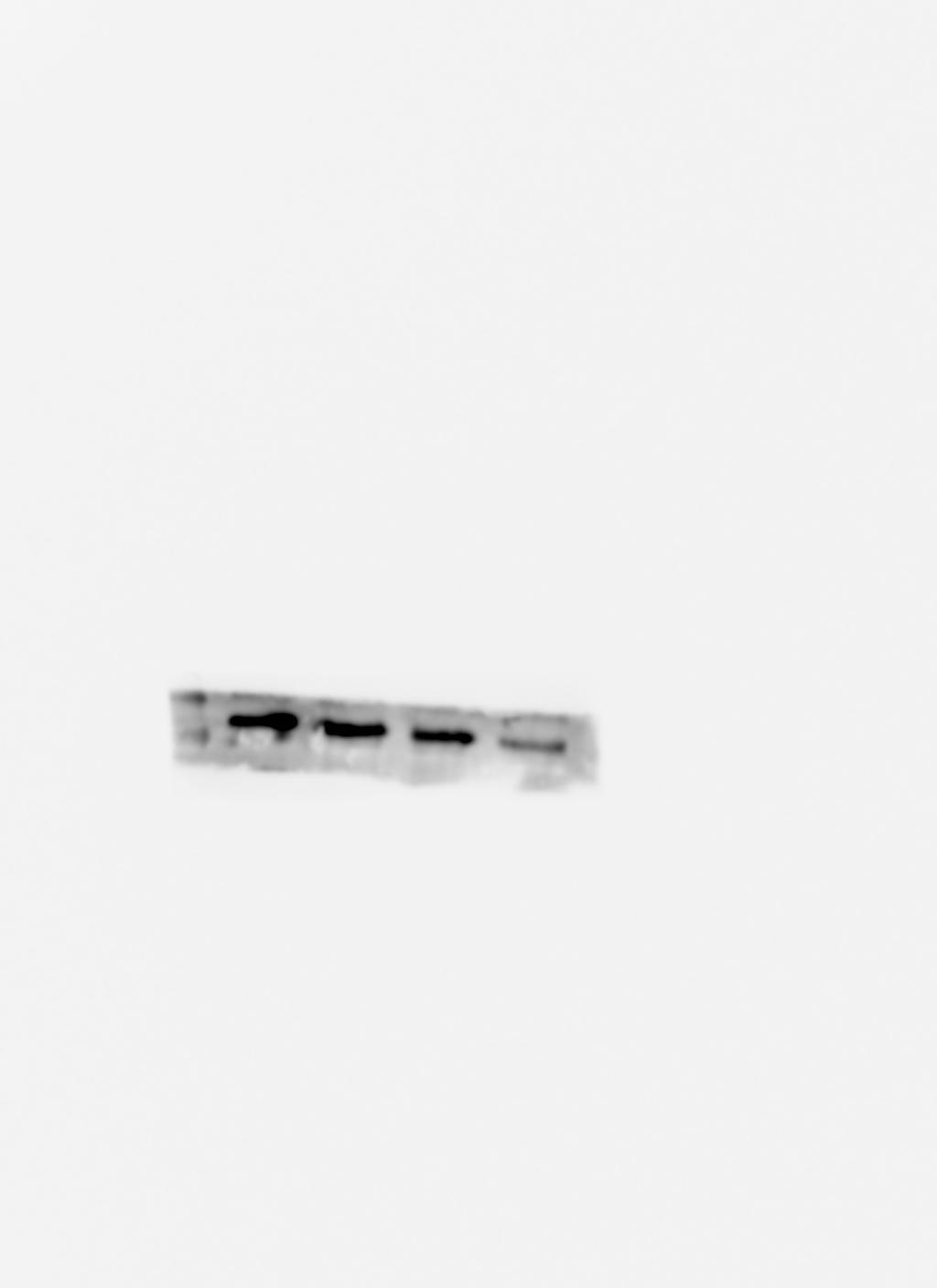

Supplement: Supplemental Information 2 [file peerj-10-14267-s002.zip › uncropped blots/figure3-uncropped blots/B-uncropped blots/p62-2.jpg]

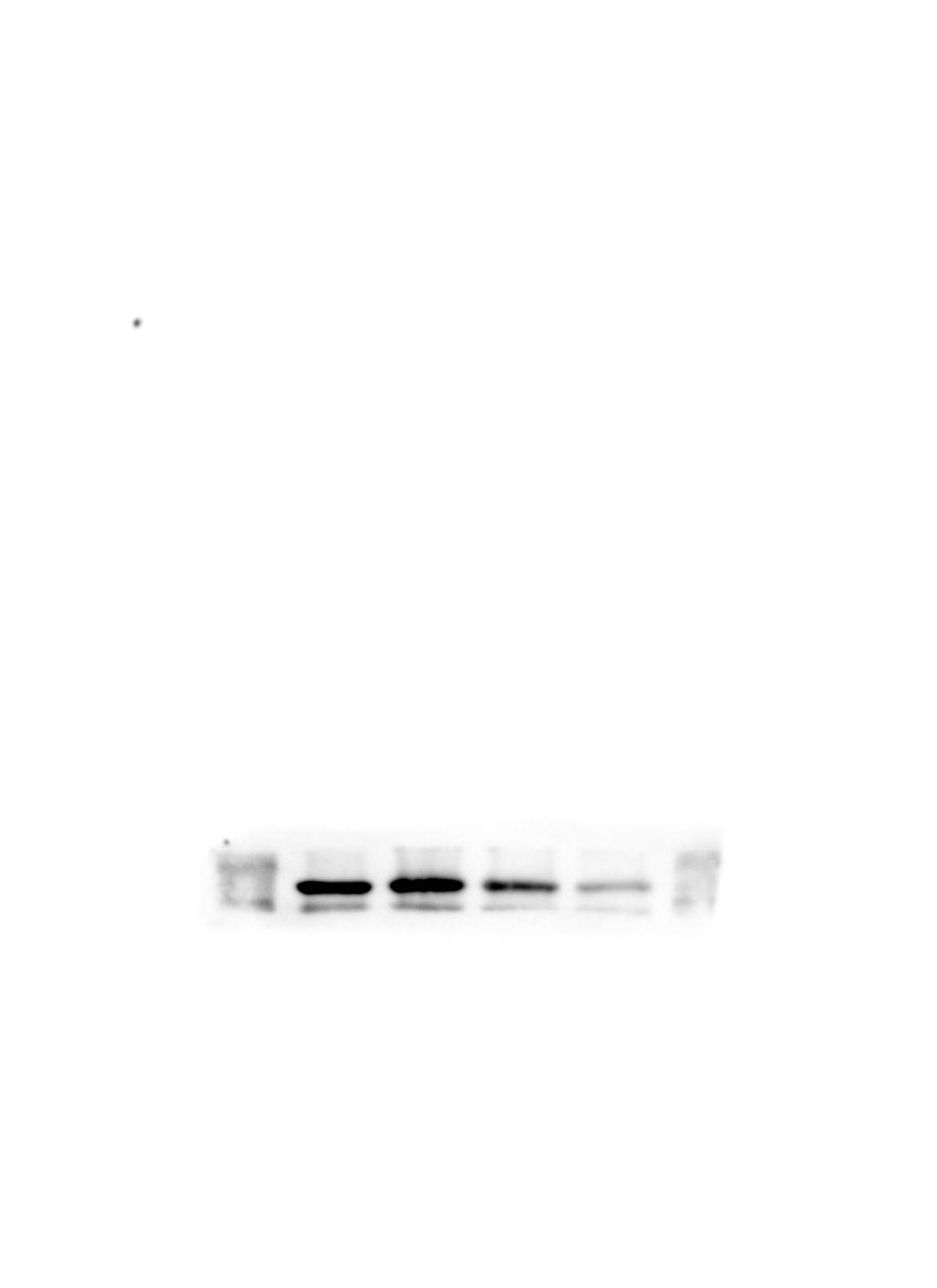

Supplement: Supplemental Information 2 [file peerj-10-14267-s002.zip › uncropped blots/figure3-uncropped blots/B-uncropped blots/p62-3.jpg]

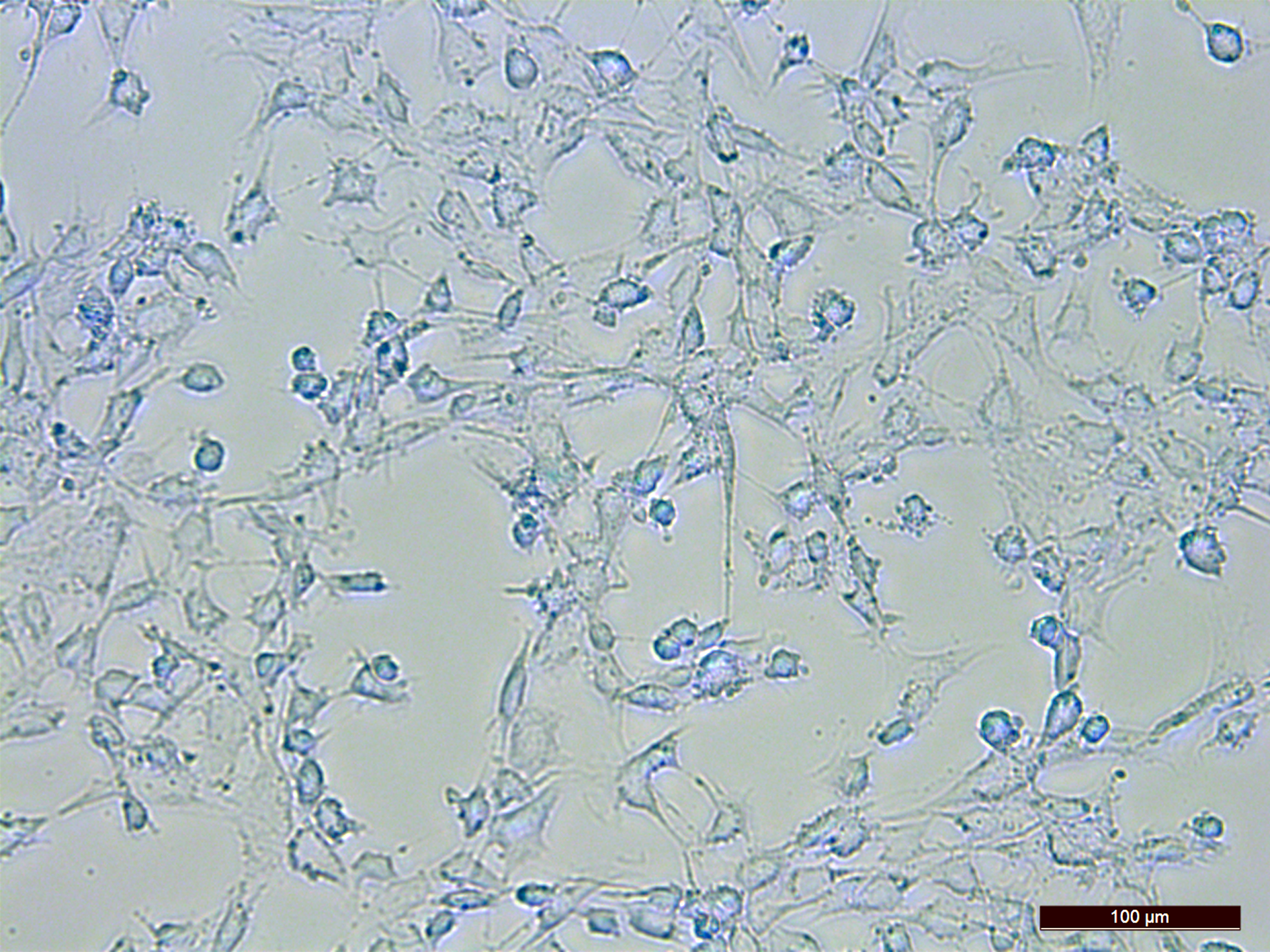

Supplement: Supplemental Information 3 — Raw data for ABR thresholds, protein expression, SA- β-gal positive cells and IOD [file peerj-10-14267-s003.zip › Raw date(figure1, 2)/figure2/SA-a┬-gal/3-MA 10mM.png]

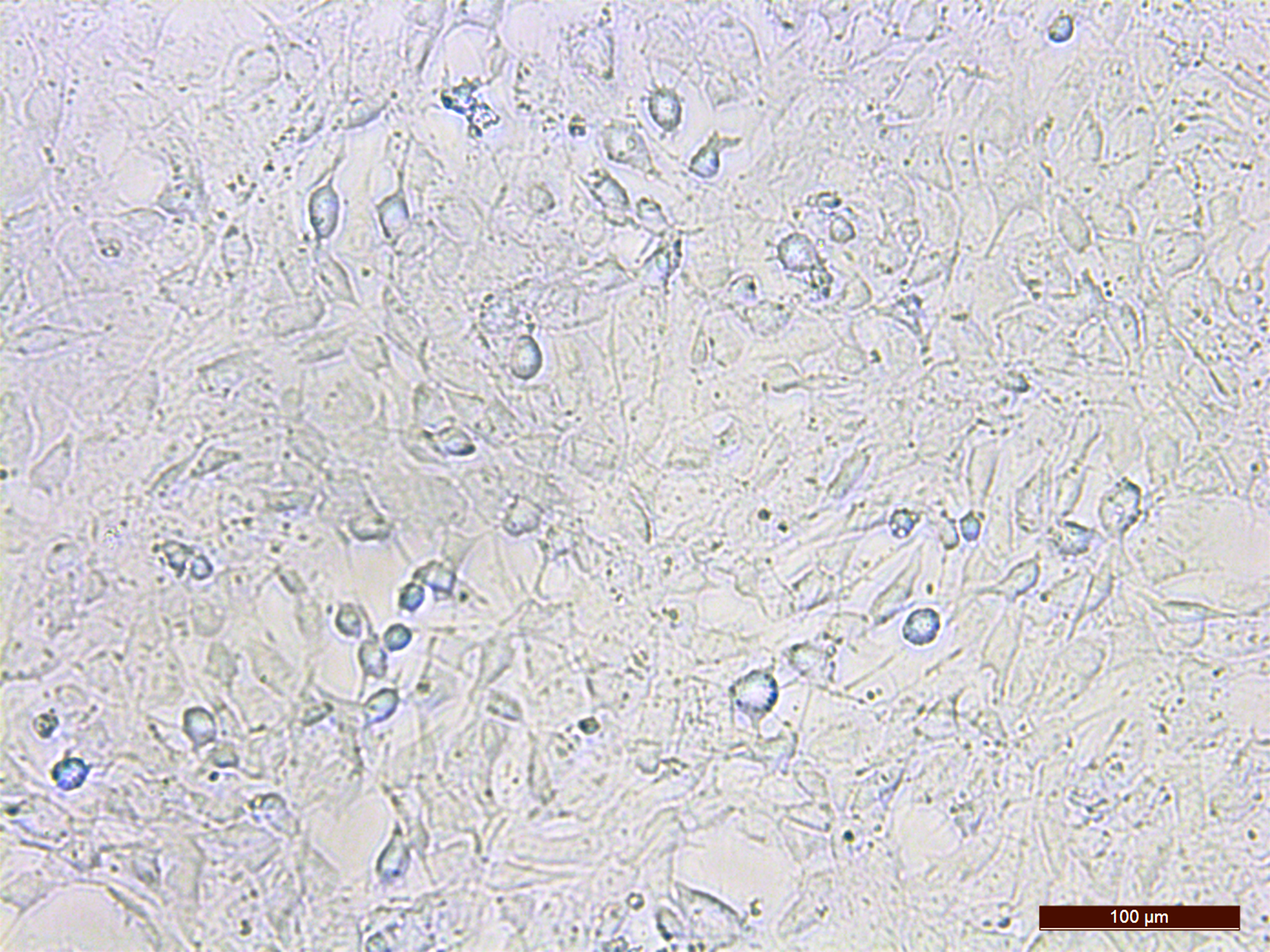

Supplement: Supplemental Information 3 — Raw data for ABR thresholds, protein expression, SA- β-gal positive cells and IOD [file peerj-10-14267-s003.zip › Raw date(figure1, 2)/figure2/SA-a┬-gal/3-MA 20mM.png]

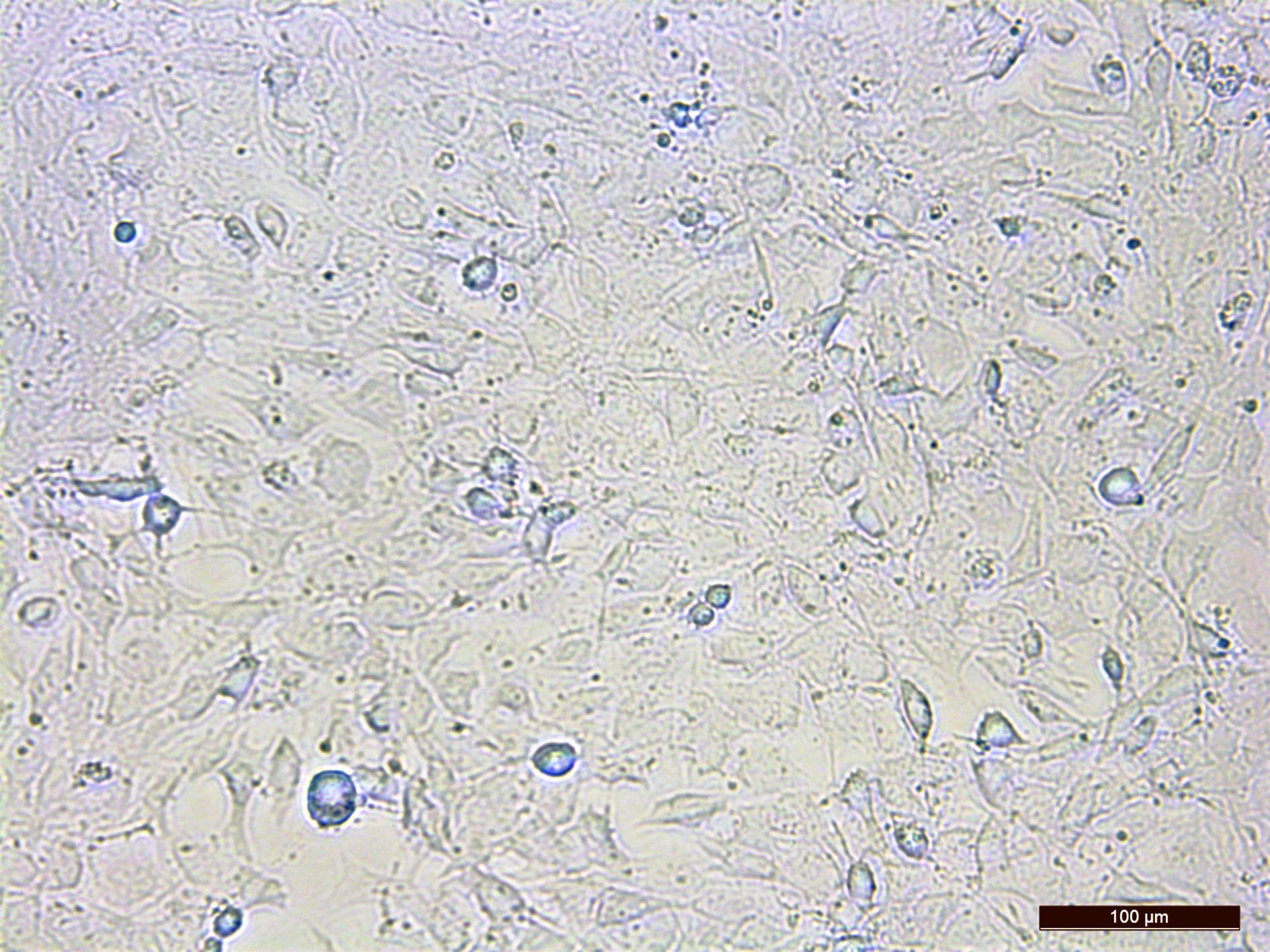

Supplement: Supplemental Information 3 — Raw data for ABR thresholds, protein expression, SA- β-gal positive cells and IOD [file peerj-10-14267-s003.zip › Raw date(figure1, 2)/figure2/SA-a┬-gal/Control.png]

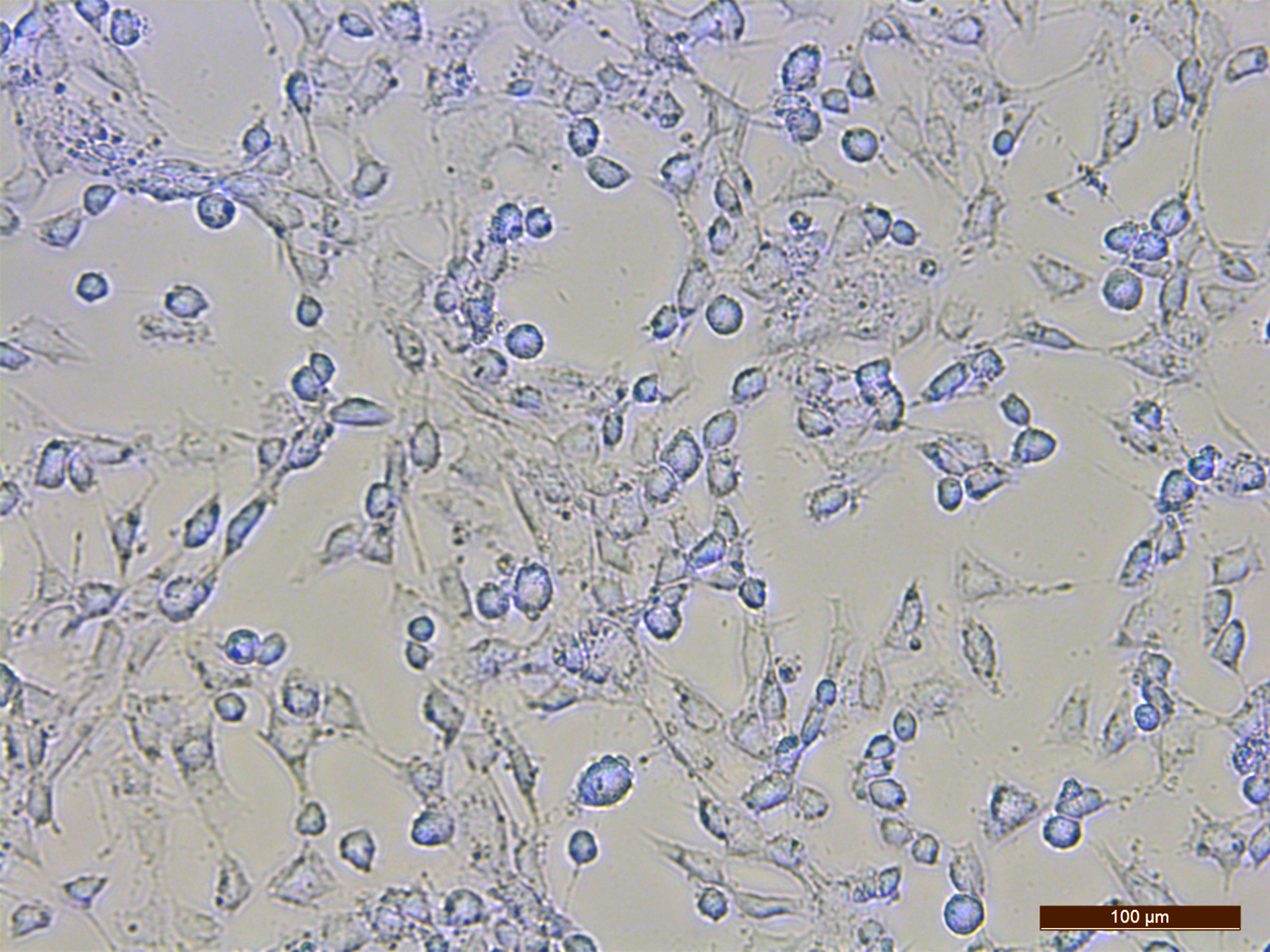

Supplement: Supplemental Information 3 — Raw data for ABR thresholds, protein expression, SA- β-gal positive cells and IOD [file peerj-10-14267-s003.zip › Raw date(figure1, 2)/figure2/SA-a┬-gal/H2O2-8h.png]

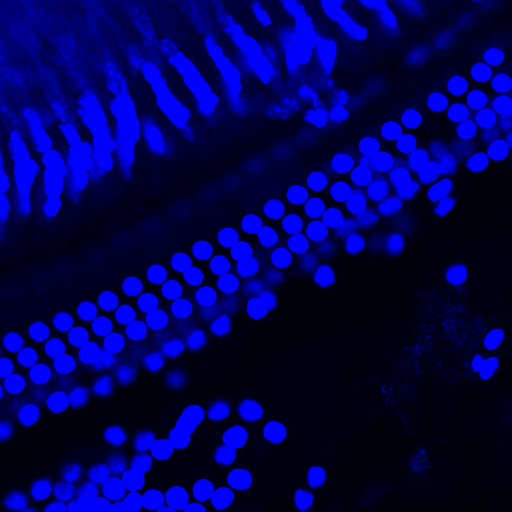

Supplement: Supplemental Information 3 — Raw data for ABR thresholds, protein expression, SA- β-gal positive cells and IOD [file peerj-10-14267-s003.zip › Raw date(figure1, 2)/figure4/LC3/Control/Image0024_C001.png]

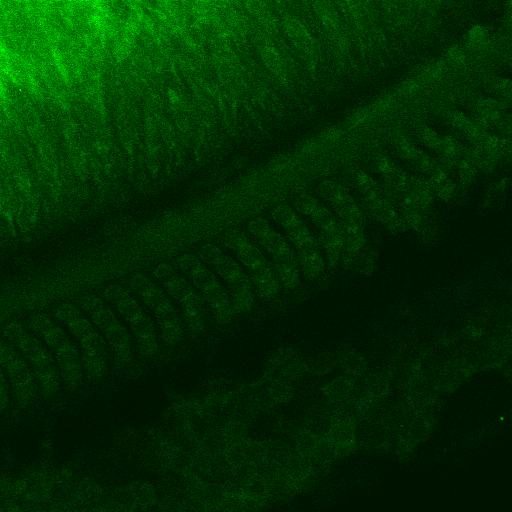

Supplement: Supplemental Information 3 — Raw data for ABR thresholds, protein expression, SA- β-gal positive cells and IOD [file peerj-10-14267-s003.zip › Raw date(figure1, 2)/figure4/LC3/Control/Image0027_C002.png]

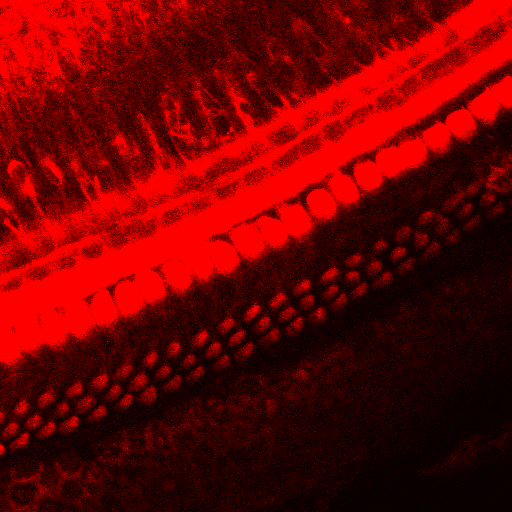

Supplement: Supplemental Information 3 — Raw data for ABR thresholds, protein expression, SA- β-gal positive cells and IOD [file peerj-10-14267-s003.zip › Raw date(figure1, 2)/figure4/LC3/Control/Image0028_C003.png]

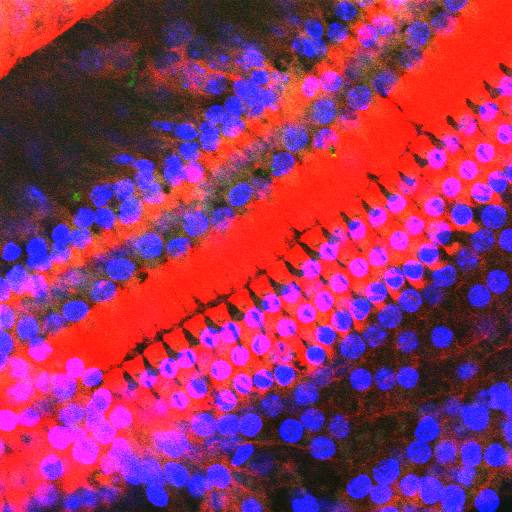

Supplement: Supplemental Information 3 — Raw data for ABR thresholds, protein expression, SA- β-gal positive cells and IOD [file peerj-10-14267-s003.zip › Raw date(figure1, 2)/figure4/LC3/Juglone/Image0039_.png]

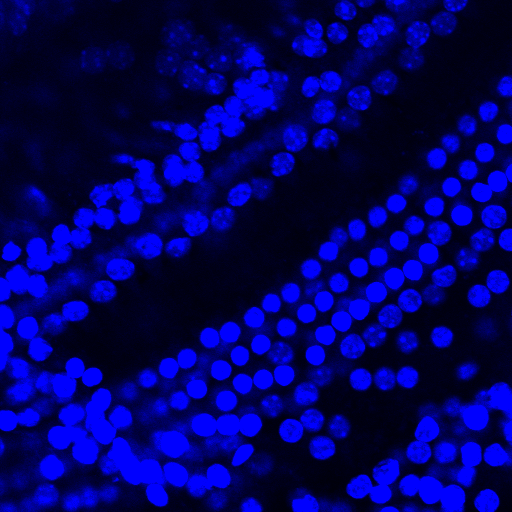

Supplement: Supplemental Information 3 — Raw data for ABR thresholds, protein expression, SA- β-gal positive cells and IOD [file peerj-10-14267-s003.zip › Raw date(figure1, 2)/figure4/LC3/Juglone/Image0039_C001.png]

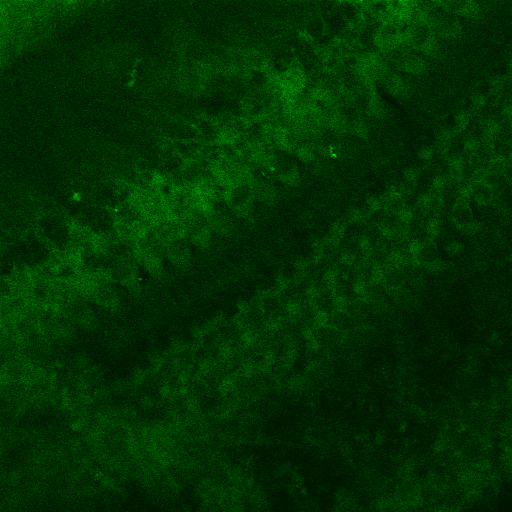

Supplement: Supplemental Information 3 — Raw data for ABR thresholds, protein expression, SA- β-gal positive cells and IOD [file peerj-10-14267-s003.zip › Raw date(figure1, 2)/figure4/LC3/Juglone/Image0039_C002.png]

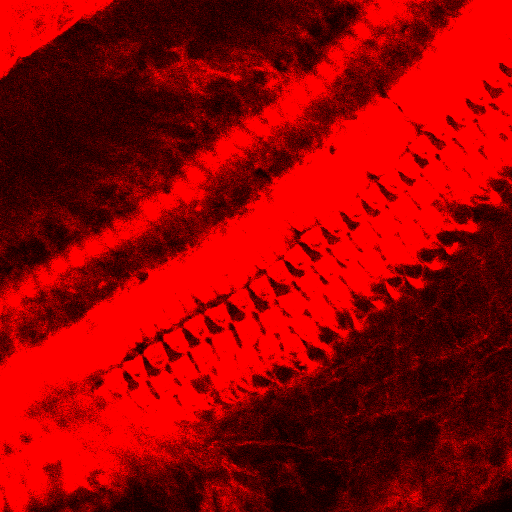

Supplement: Supplemental Information 3 — Raw data for ABR thresholds, protein expression, SA- β-gal positive cells and IOD [file peerj-10-14267-s003.zip › Raw date(figure1, 2)/figure4/LC3/Juglone/Image0039_C003.png]

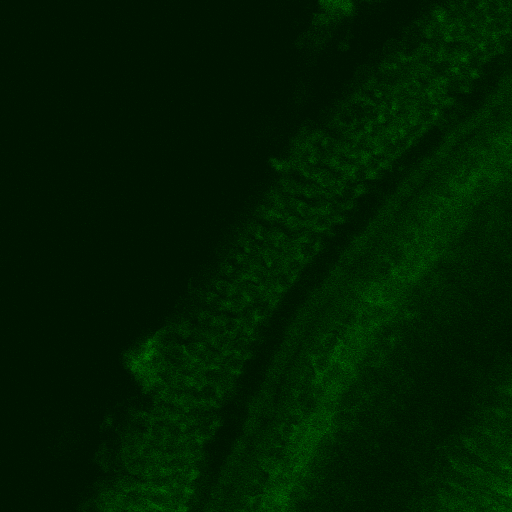

Supplement: Supplemental Information 3 — Raw data for ABR thresholds, protein expression, SA- β-gal positive cells and IOD [file peerj-10-14267-s003.zip › Raw date(figure1, 2)/figure4/p62/Control/Image0012.png]

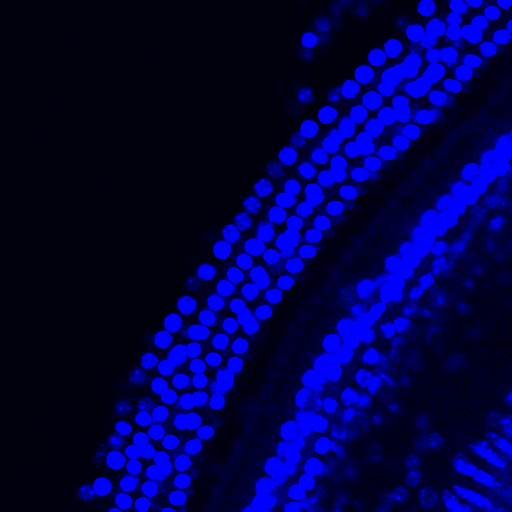

Supplement: Supplemental Information 3 — Raw data for ABR thresholds, protein expression, SA- β-gal positive cells and IOD [file peerj-10-14267-s003.zip › Raw date(figure1, 2)/figure4/p62/Control/Image0012-1.png]

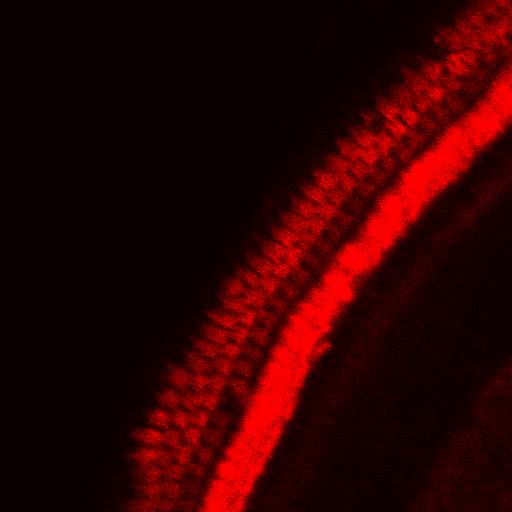

Supplement: Supplemental Information 3 — Raw data for ABR thresholds, protein expression, SA- β-gal positive cells and IOD [file peerj-10-14267-s003.zip › Raw date(figure1, 2)/figure4/p62/Control/Image0013_C003.jpg]

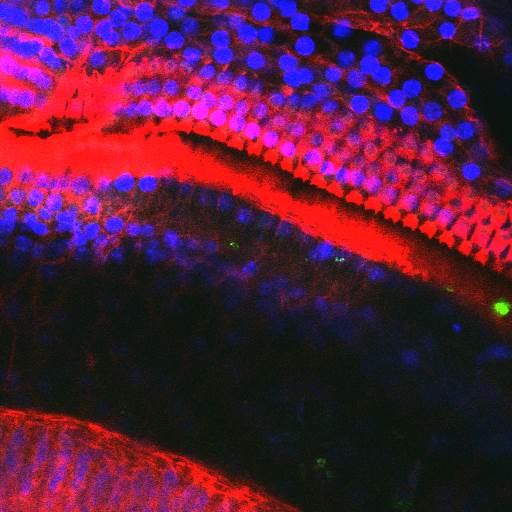

Supplement: Supplemental Information 3 — Raw data for ABR thresholds, protein expression, SA- β-gal positive cells and IOD [file peerj-10-14267-s003.zip › Raw date(figure1, 2)/figure4/p62/Juglone/Image0005_.jpg]

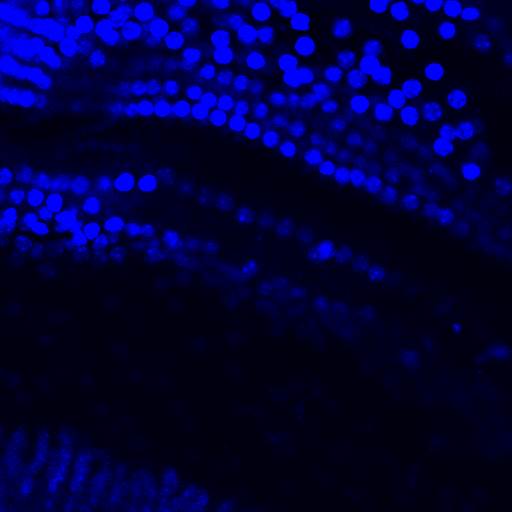

Supplement: Supplemental Information 3 — Raw data for ABR thresholds, protein expression, SA- β-gal positive cells and IOD [file peerj-10-14267-s003.zip › Raw date(figure1, 2)/figure4/p62/Juglone/Image0005_C001.jpg]

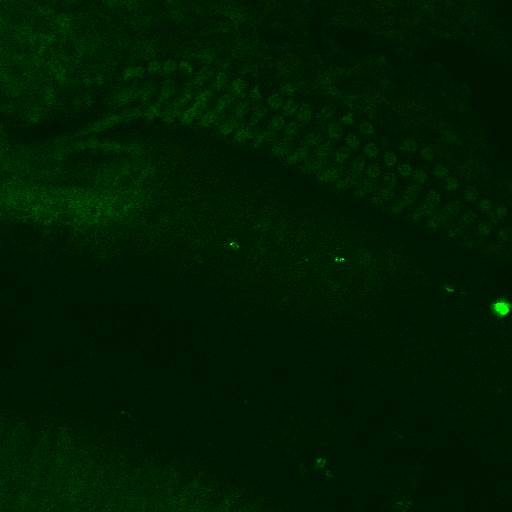

Supplement: Supplemental Information 3 — Raw data for ABR thresholds, protein expression, SA- β-gal positive cells and IOD [file peerj-10-14267-s003.zip › Raw date(figure1, 2)/figure4/p62/Juglone/Image0005_C002.jpg]

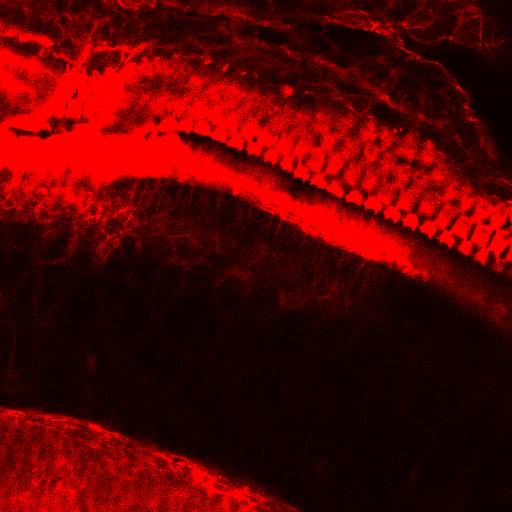

Supplement: Supplemental Information 3 — Raw data for ABR thresholds, protein expression, SA- β-gal positive cells and IOD [file peerj-10-14267-s003.zip › Raw date(figure1, 2)/figure4/p62/Juglone/Image0005_C003.jpg]

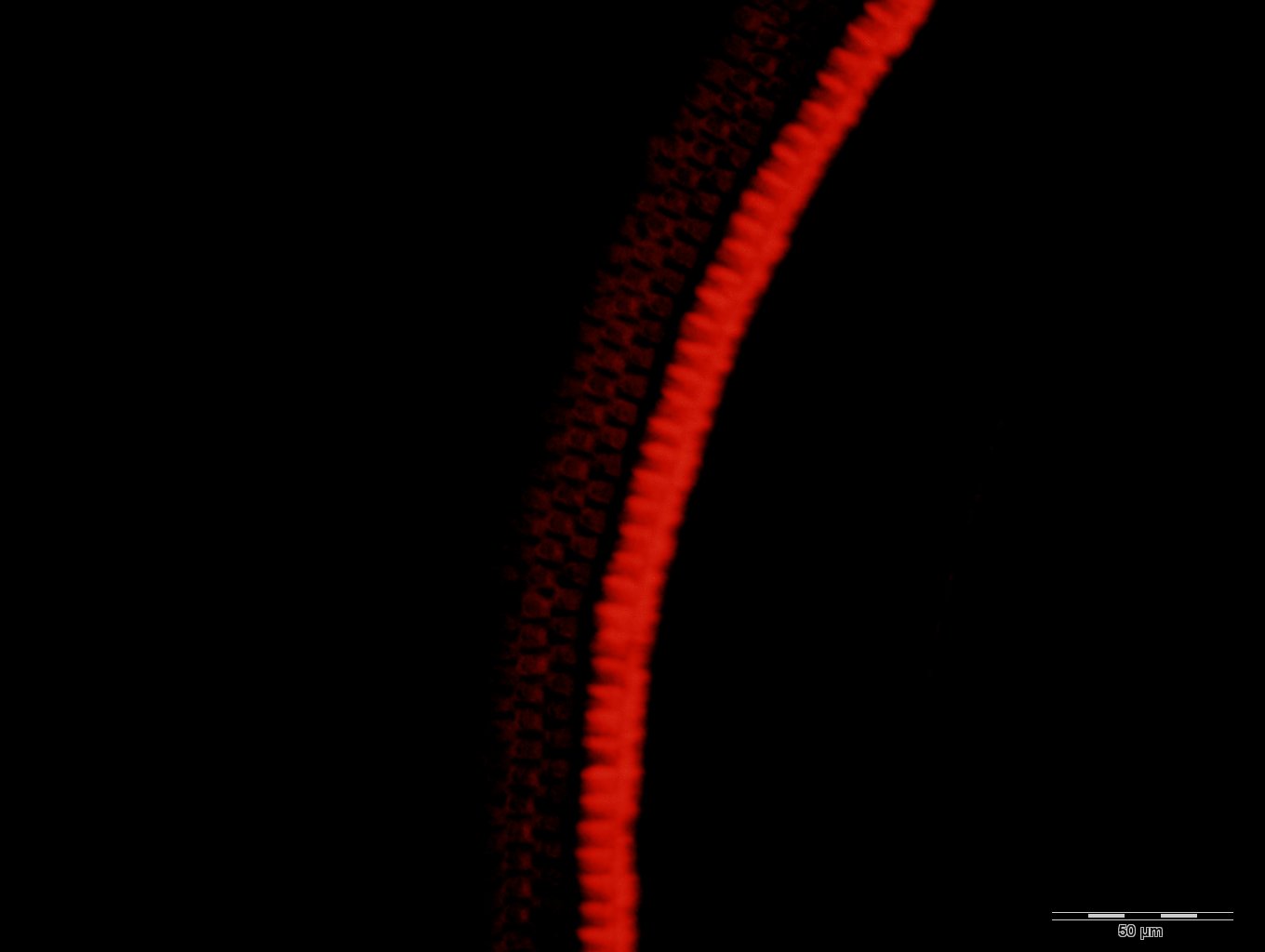

Supplement: Supplemental Information 3 — Raw data for ABR thresholds, protein expression, SA- β-gal positive cells and IOD [file peerj-10-14267-s003.zip › Raw date(figure1, 2)/figure4/PIN1/Control/═╝╧±_146866.jpg]

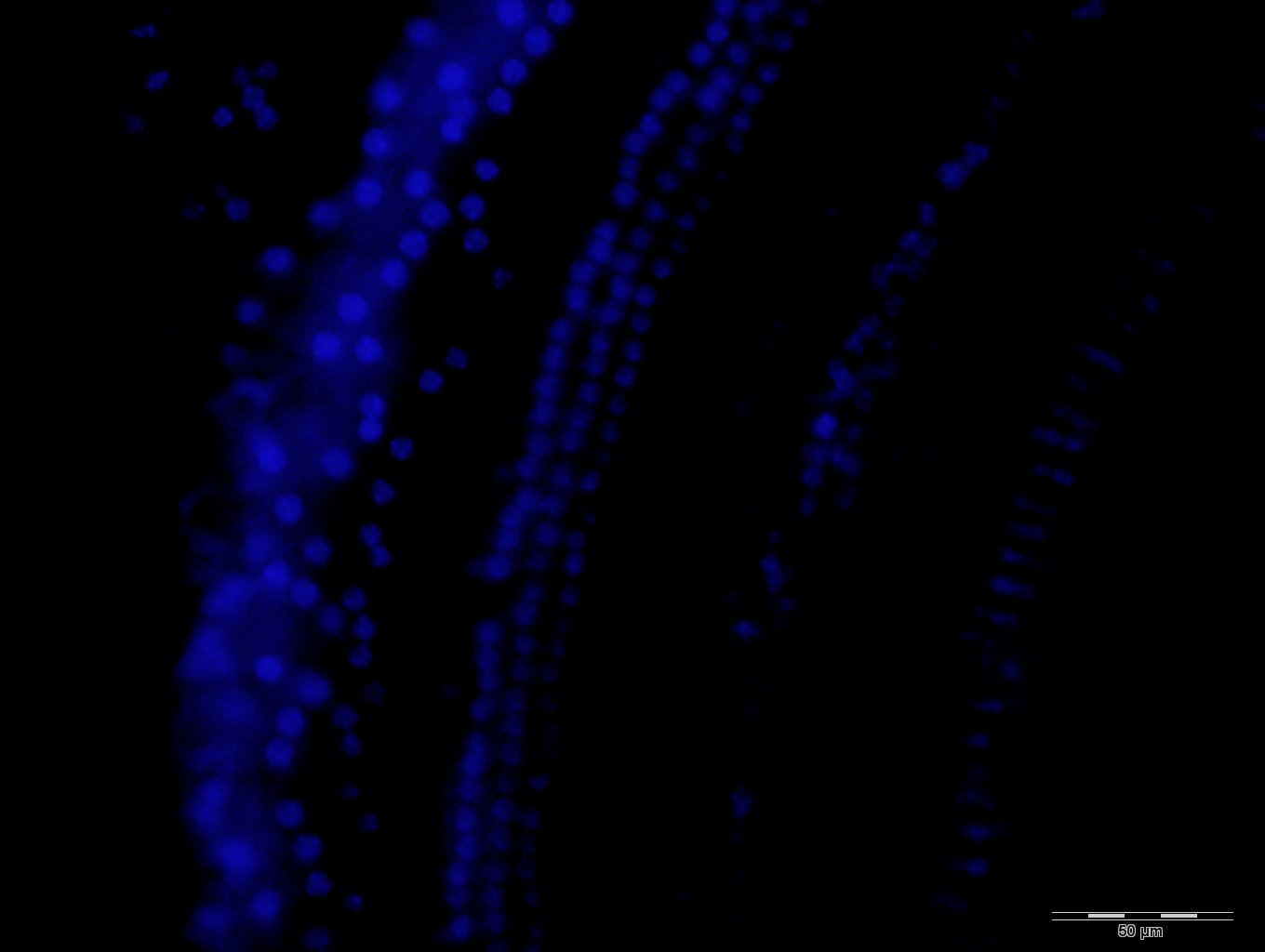

Supplement: Supplemental Information 3 — Raw data for ABR thresholds, protein expression, SA- β-gal positive cells and IOD [file peerj-10-14267-s003.zip › Raw date(figure1, 2)/figure4/PIN1/Control/═╝╧±_146867.jpg]

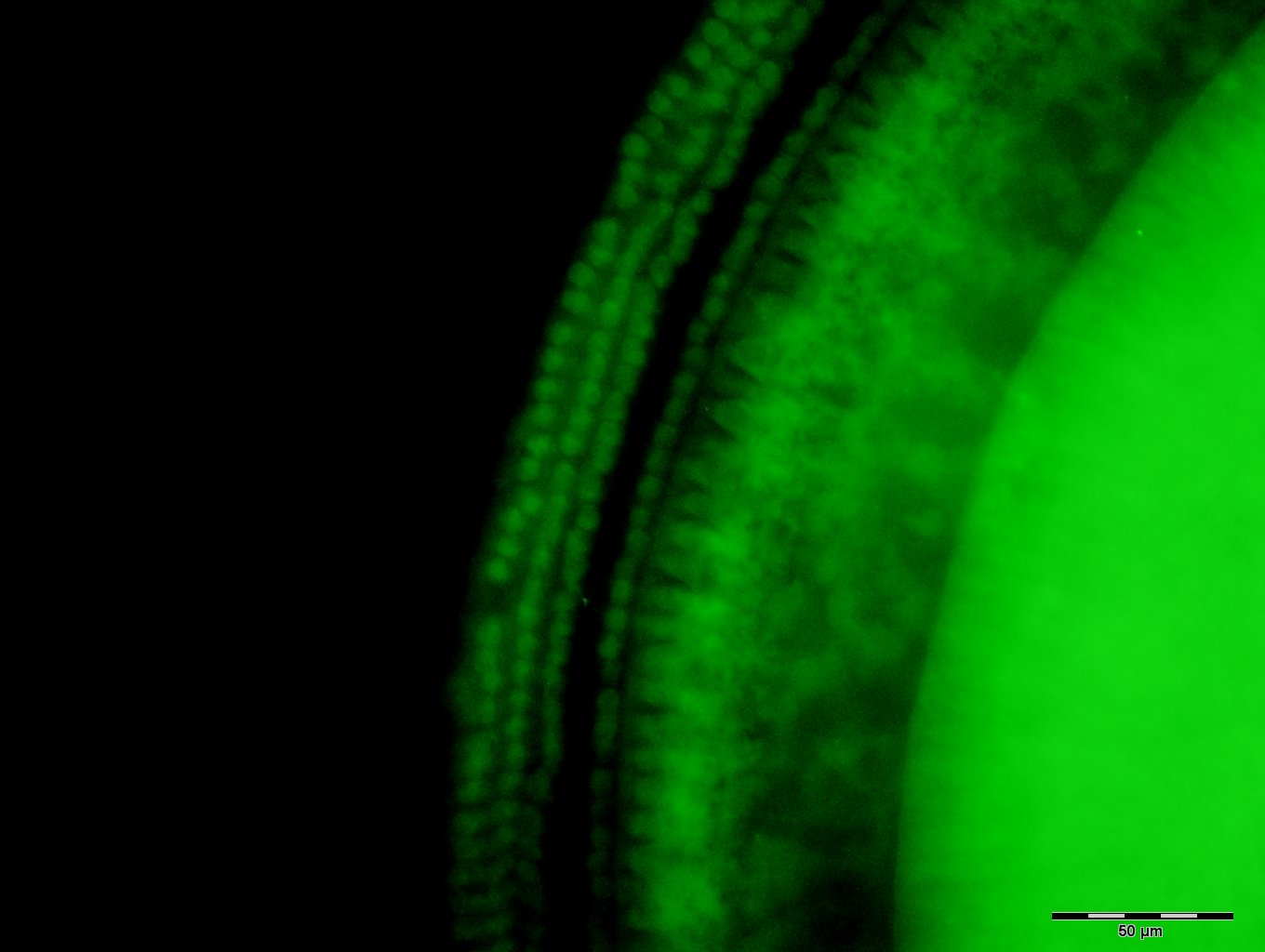

Supplement: Supplemental Information 3 — Raw data for ABR thresholds, protein expression, SA- β-gal positive cells and IOD [file peerj-10-14267-s003.zip › Raw date(figure1, 2)/figure4/PIN1/Control/═╝╧±_146868.jpg]

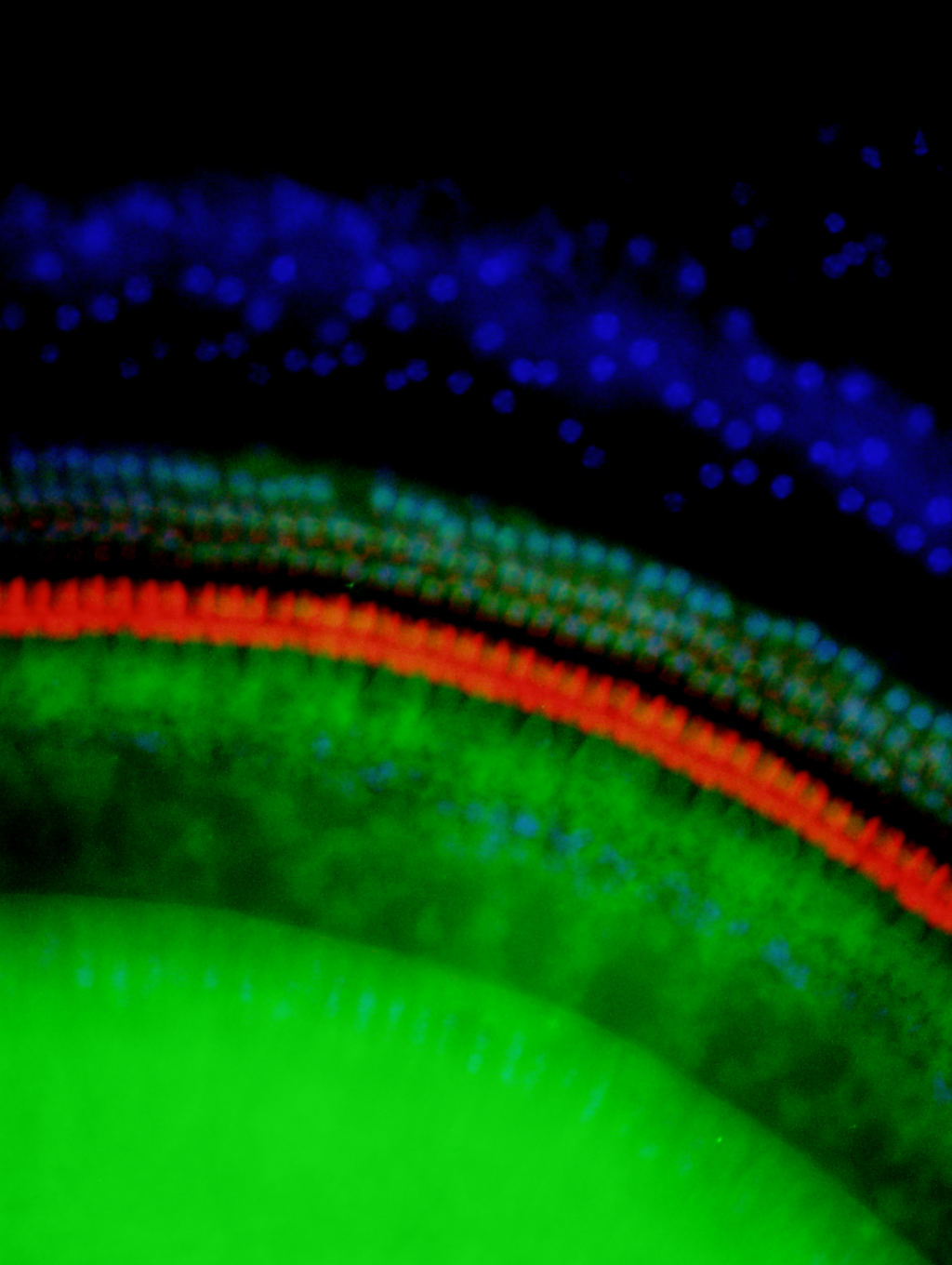

Supplement: Supplemental Information 3 — Raw data for ABR thresholds, protein expression, SA- β-gal positive cells and IOD [file peerj-10-14267-s003.zip › Raw date(figure1, 2)/figure4/PIN1/Control/═╝╧±_26.png]

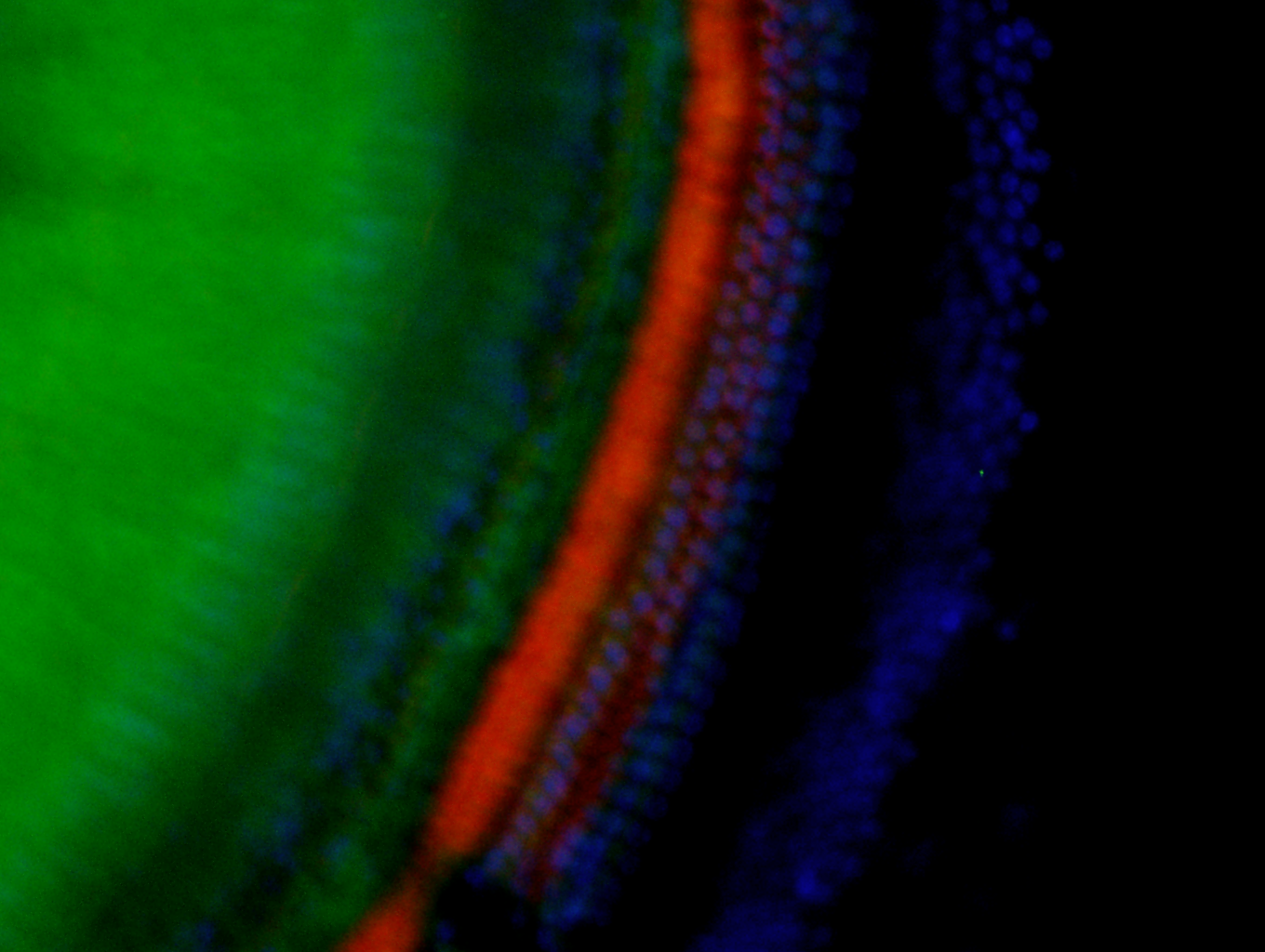

Supplement: Supplemental Information 3 — Raw data for ABR thresholds, protein expression, SA- β-gal positive cells and IOD [file peerj-10-14267-s003.zip › Raw date(figure1, 2)/figure4/PIN1/Juglone/09.png]

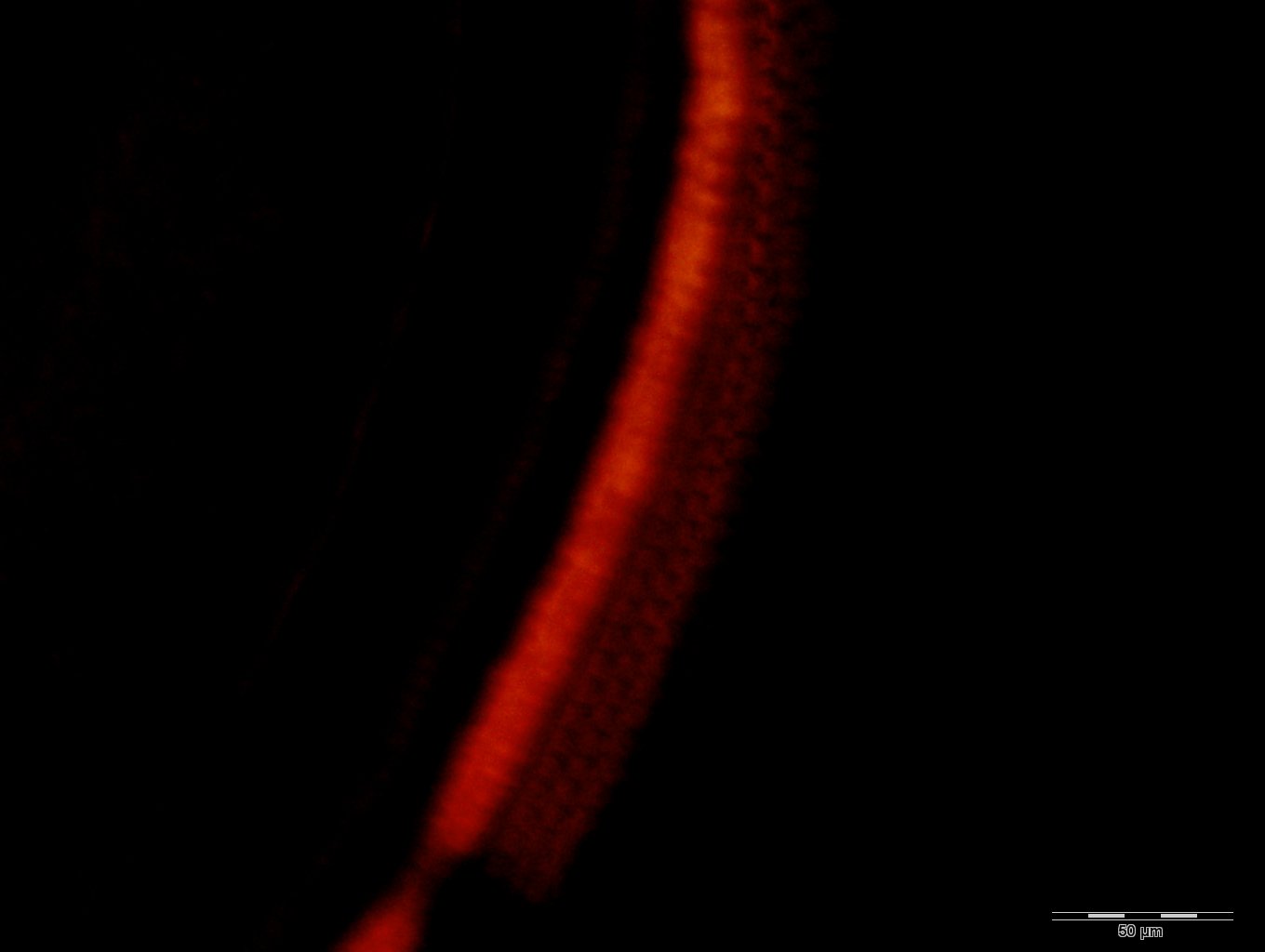

Supplement: Supplemental Information 3 — Raw data for ABR thresholds, protein expression, SA- β-gal positive cells and IOD [file peerj-10-14267-s003.zip › Raw date(figure1, 2)/figure4/PIN1/Juglone/147703.jpg]

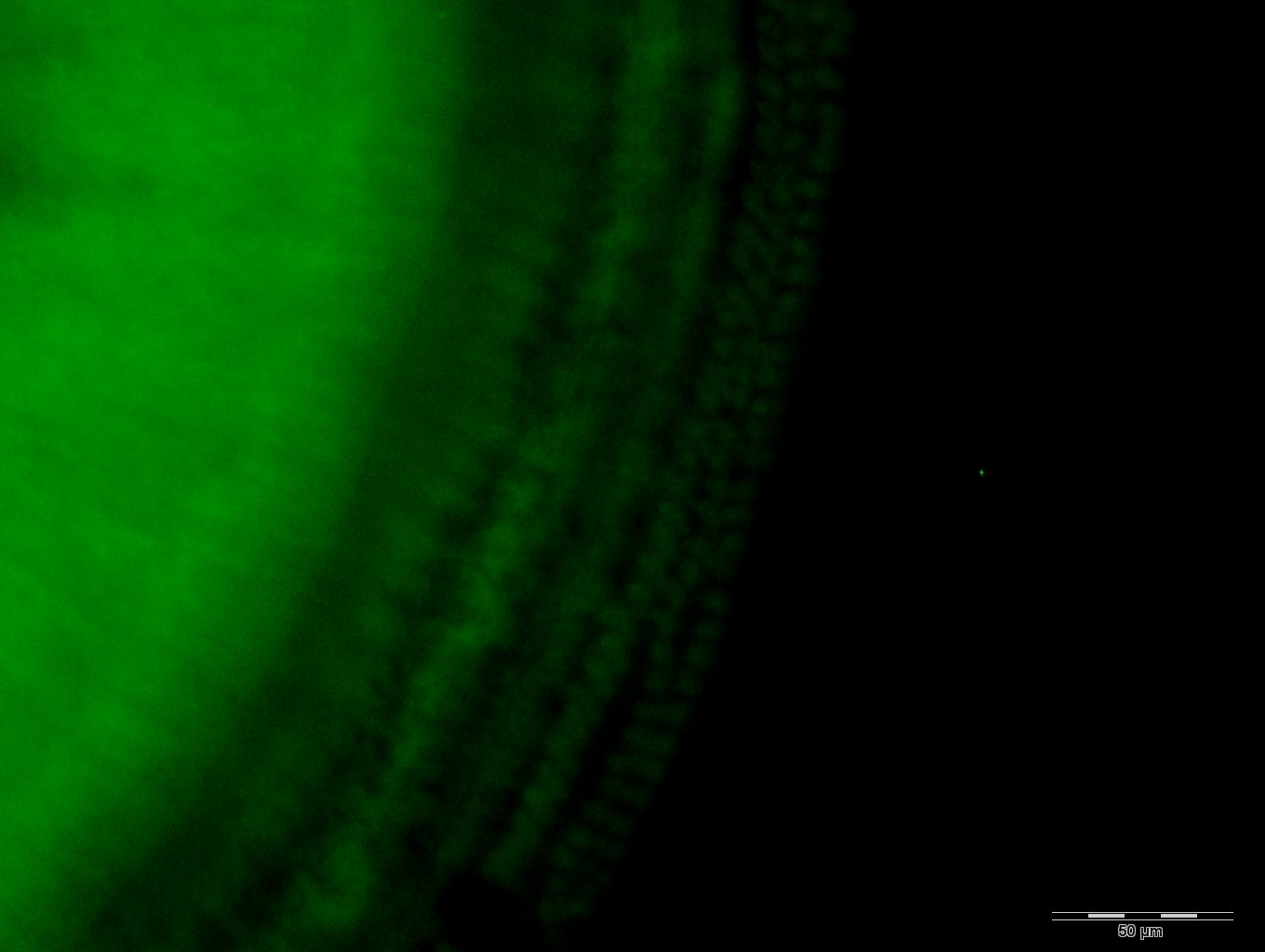

Supplement: Supplemental Information 3 — Raw data for ABR thresholds, protein expression, SA- β-gal positive cells and IOD [file peerj-10-14267-s003.zip › Raw date(figure1, 2)/figure4/PIN1/Juglone/147704.jpg]

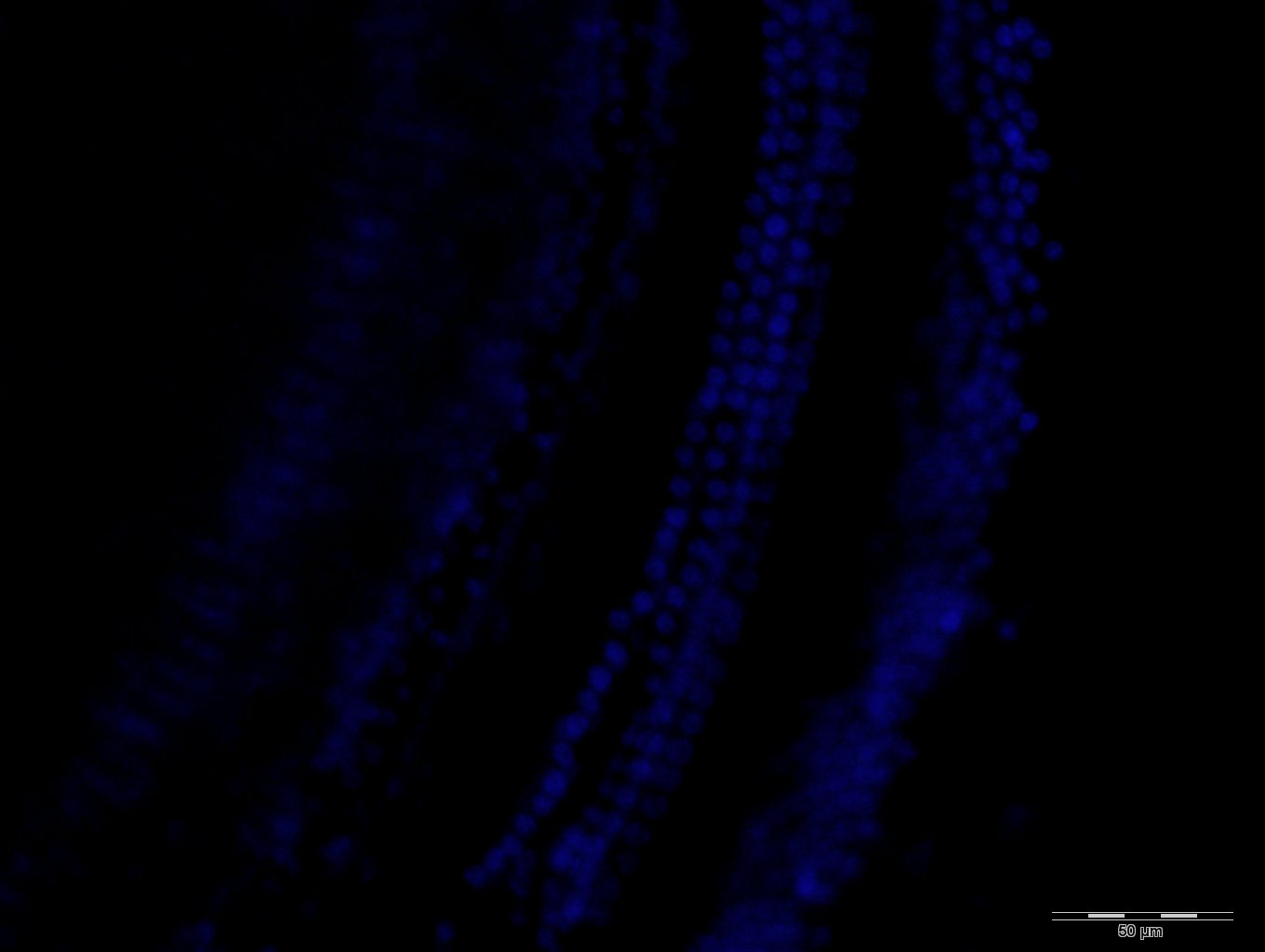

Supplement: Supplemental Information 3 — Raw data for ABR thresholds, protein expression, SA- β-gal positive cells and IOD [file peerj-10-14267-s003.zip › Raw date(figure1, 2)/figure4/PIN1/Juglone/147705.jpg]

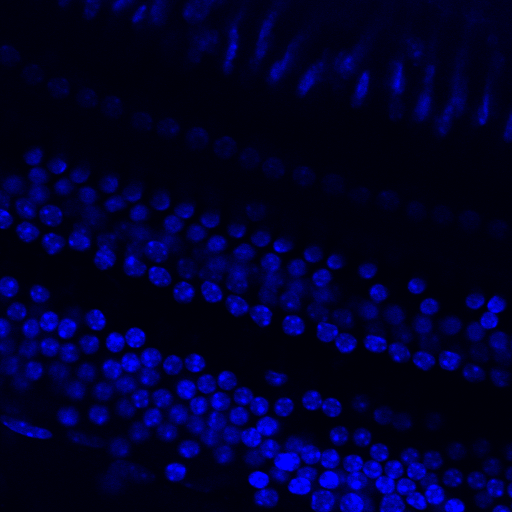

Supplement: Supplemental Information 4 — Raw data for ABR thresholds, protein expression, SA- β-gal positive cells [file peerj-10-14267-s004.zip › figure1/LC3B-Figure1/LC3B-Middle/Image0032_C001.png]

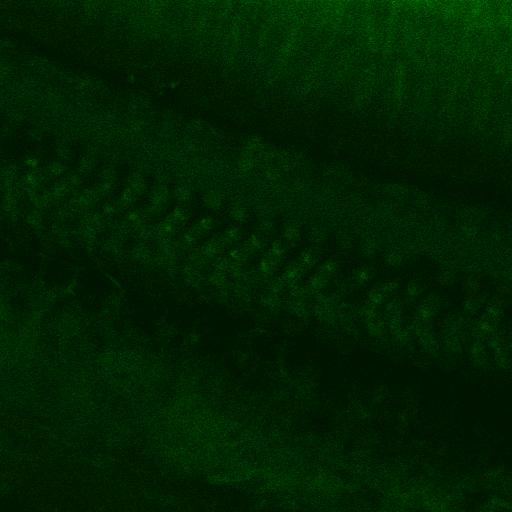

Supplement: Supplemental Information 4 — Raw data for ABR thresholds, protein expression, SA- β-gal positive cells [file peerj-10-14267-s004.zip › figure1/LC3B-Figure1/LC3B-Middle/Image0032_C002.png]

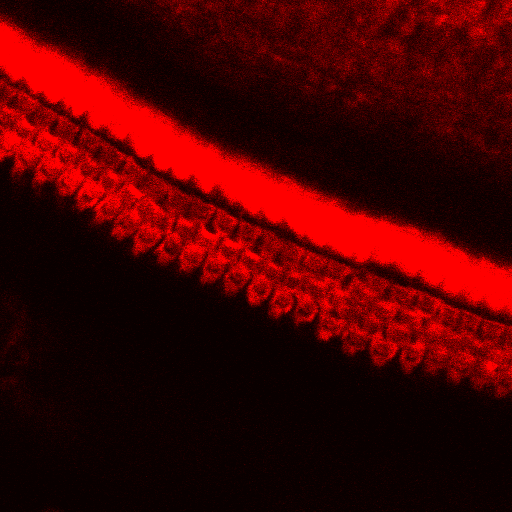

Supplement: Supplemental Information 4 — Raw data for ABR thresholds, protein expression, SA- β-gal positive cells [file peerj-10-14267-s004.zip › figure1/LC3B-Figure1/LC3B-Middle/Image0033_C003.png]

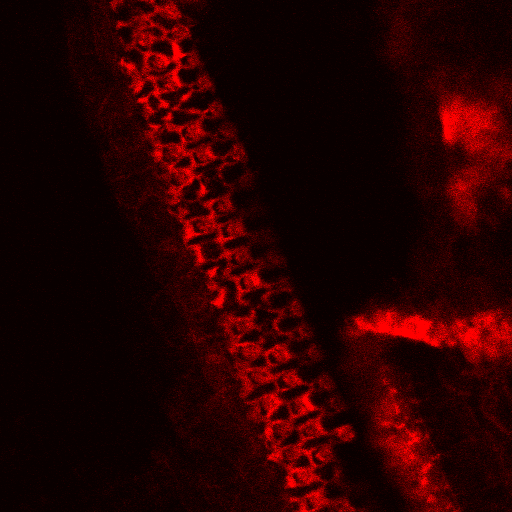

Supplement: Supplemental Information 4 — Raw data for ABR thresholds, protein expression, SA- β-gal positive cells [file peerj-10-14267-s004.zip › figure1/LC3B-Figure1/LC3B-Old/lc3Image0041_C003.png]

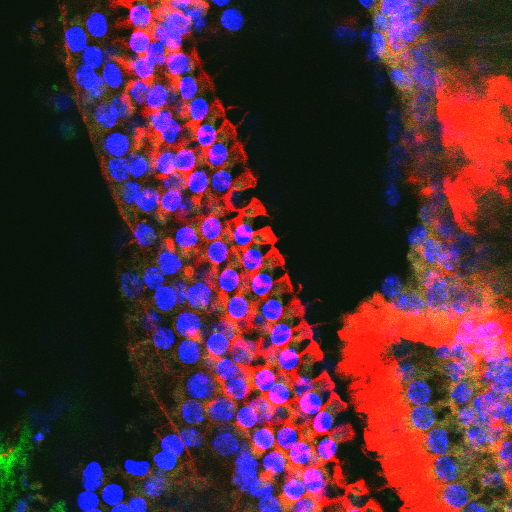

Supplement: Supplemental Information 4 — Raw data for ABR thresholds, protein expression, SA- β-gal positive cells [file peerj-10-14267-s004.zip › figure1/LC3B-Figure1/LC3B-Old/lc3Image0042_.png]

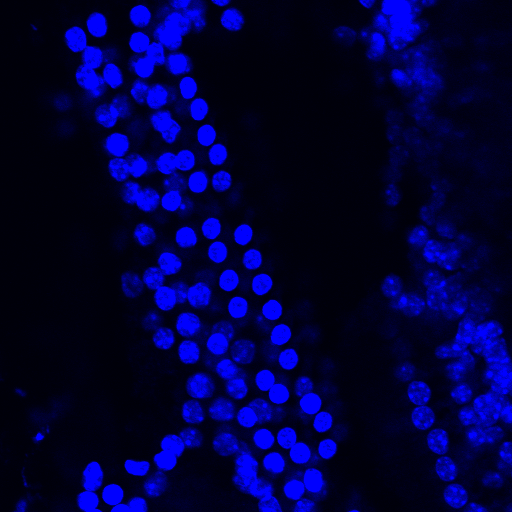

Supplement: Supplemental Information 4 — Raw data for ABR thresholds, protein expression, SA- β-gal positive cells [file peerj-10-14267-s004.zip › figure1/LC3B-Figure1/LC3B-Old/lc3Image0042_C001.png]

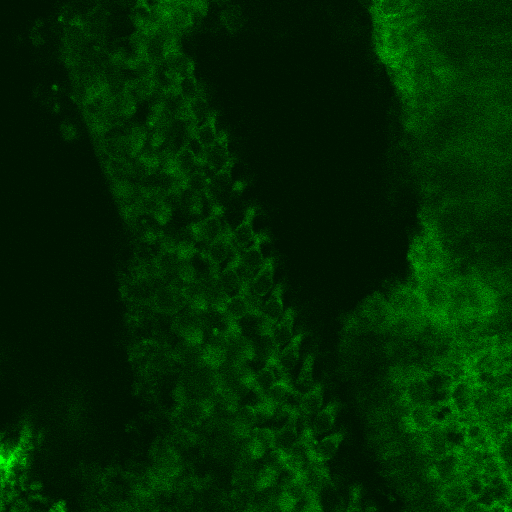

Supplement: Supplemental Information 4 — Raw data for ABR thresholds, protein expression, SA- β-gal positive cells [file peerj-10-14267-s004.zip › figure1/LC3B-Figure1/LC3B-Old/lc3Image0042_C002.png]

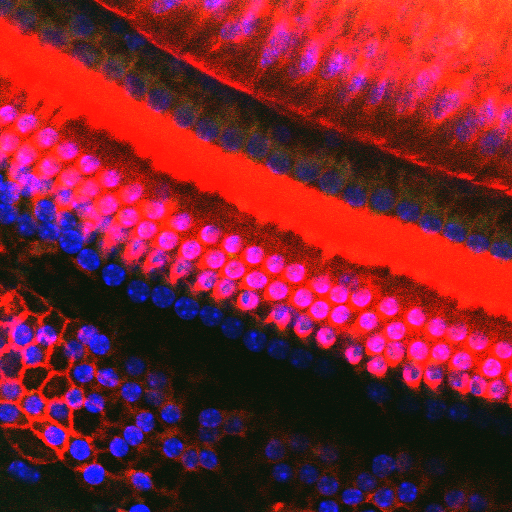

Supplement: Supplemental Information 4 — Raw data for ABR thresholds, protein expression, SA- β-gal positive cells [file peerj-10-14267-s004.zip › figure1/LC3B-Figure1/LC3B-Young/Image0034_.png]

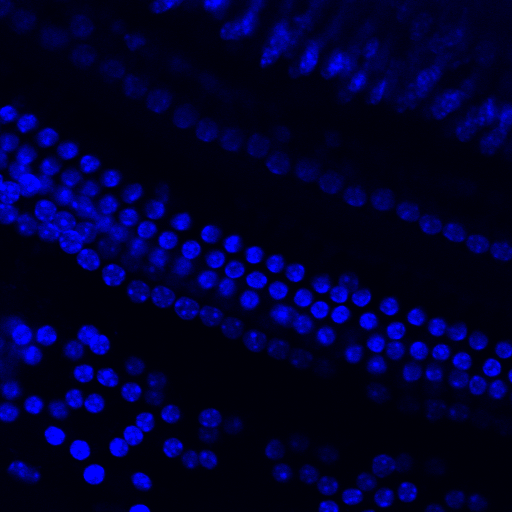

Supplement: Supplemental Information 4 — Raw data for ABR thresholds, protein expression, SA- β-gal positive cells [file peerj-10-14267-s004.zip › figure1/LC3B-Figure1/LC3B-Young/Image0034_C001.png]

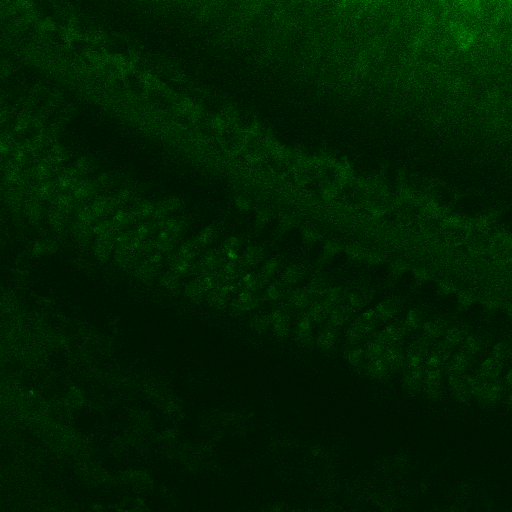

Supplement: Supplemental Information 4 — Raw data for ABR thresholds, protein expression, SA- β-gal positive cells [file peerj-10-14267-s004.zip › figure1/LC3B-Figure1/LC3B-Young/Image0034_C002.png]

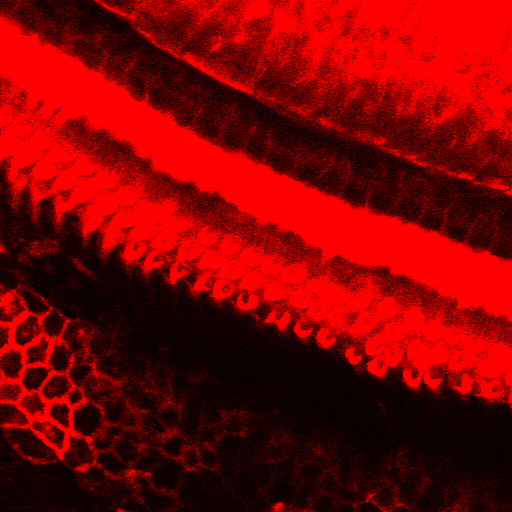

Supplement: Supplemental Information 4 — Raw data for ABR thresholds, protein expression, SA- β-gal positive cells [file peerj-10-14267-s004.zip › figure1/LC3B-Figure1/LC3B-Young/Image0034_C003.png]

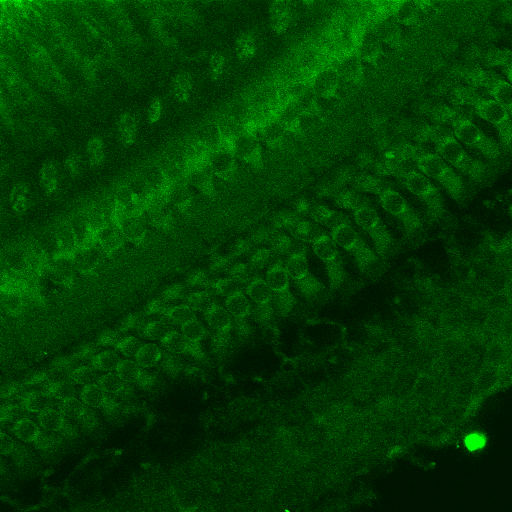

Supplement: Supplemental Information 4 — Raw data for ABR thresholds, protein expression, SA- β-gal positive cells [file peerj-10-14267-s004.zip › figure1/p62-Figure1/p62 -Young/Image0046_C002.png]

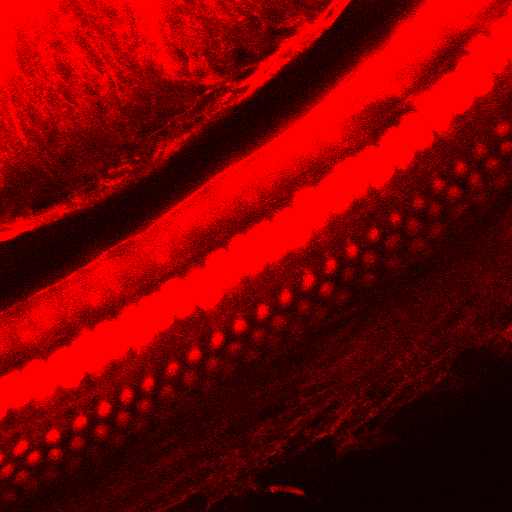

Supplement: Supplemental Information 4 — Raw data for ABR thresholds, protein expression, SA- β-gal positive cells [file peerj-10-14267-s004.zip › figure1/p62-Figure1/p62 -Young/Image0047_C003.png]
