# Supplementary figures and images for: PIN1 protects auditory hair cells from senescence via autophagy (part 2 of 2)
Source: PeerJ. 2022 Nov 1;10:e14267. doi: 10.7717/peerj.14267 (PMC9635358; doi:10.7717/peerj.14267)

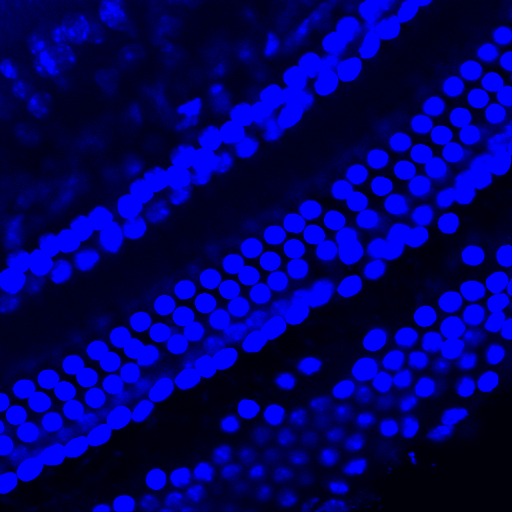

Supplement: Supplemental Information 4 — Raw data for ABR thresholds, protein expression, SA- β-gal positive cells [file peerj-10-14267-s004.zip › figure1/p62-Figure1/p62 -Young/Image0048_C001.png]

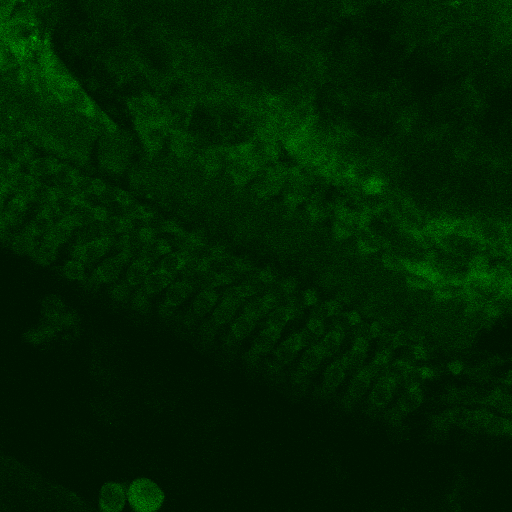

Supplement: Supplemental Information 4 — Raw data for ABR thresholds, protein expression, SA- β-gal positive cells [file peerj-10-14267-s004.zip › figure1/p62-Figure1/p62-Middle/Image0050_C002.png]

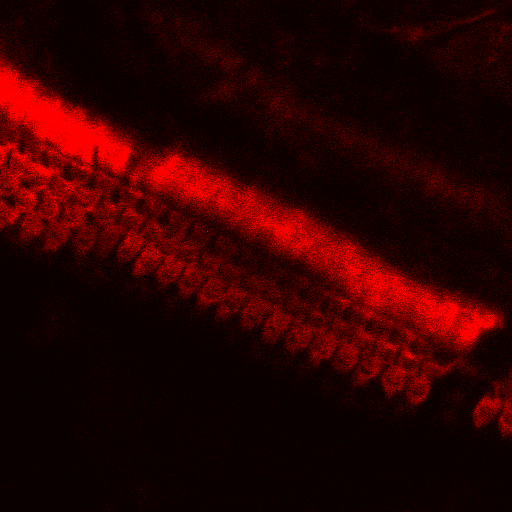

Supplement: Supplemental Information 4 — Raw data for ABR thresholds, protein expression, SA- β-gal positive cells [file peerj-10-14267-s004.zip › figure1/p62-Figure1/p62-Middle/Image0051_C003.png]

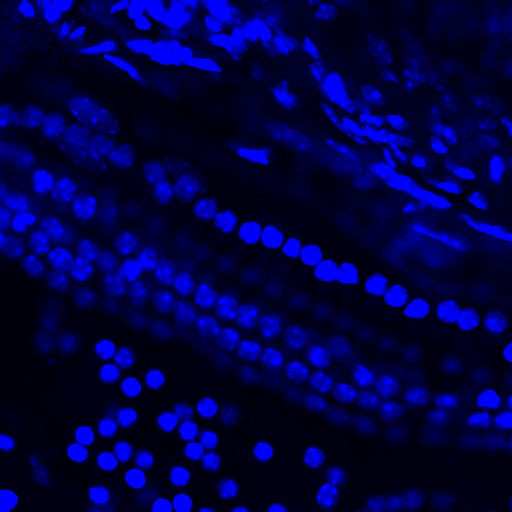

Supplement: Supplemental Information 4 — Raw data for ABR thresholds, protein expression, SA- β-gal positive cells [file peerj-10-14267-s004.zip › figure1/p62-Figure1/p62-Middle/p62mImage0049.png]

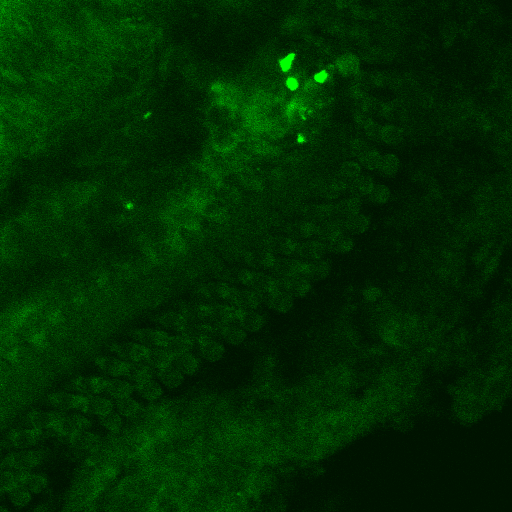

Supplement: Supplemental Information 4 — Raw data for ABR thresholds, protein expression, SA- β-gal positive cells [file peerj-10-14267-s004.zip › figure1/p62-Figure1/p62-Old/Image0054-1.png]

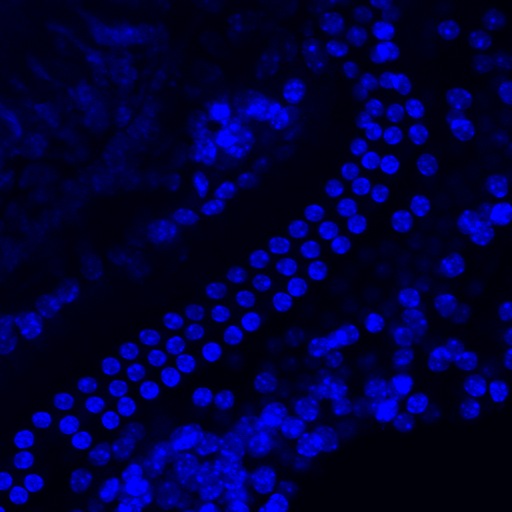

Supplement: Supplemental Information 4 — Raw data for ABR thresholds, protein expression, SA- β-gal positive cells [file peerj-10-14267-s004.zip › figure1/p62-Figure1/p62-Old/Image0054-2.png]

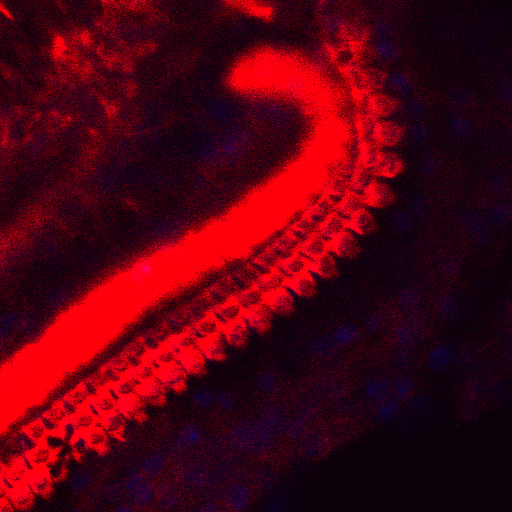

Supplement: Supplemental Information 4 — Raw data for ABR thresholds, protein expression, SA- β-gal positive cells [file peerj-10-14267-s004.zip › figure1/p62-Figure1/p62-Old/Image0055.png]

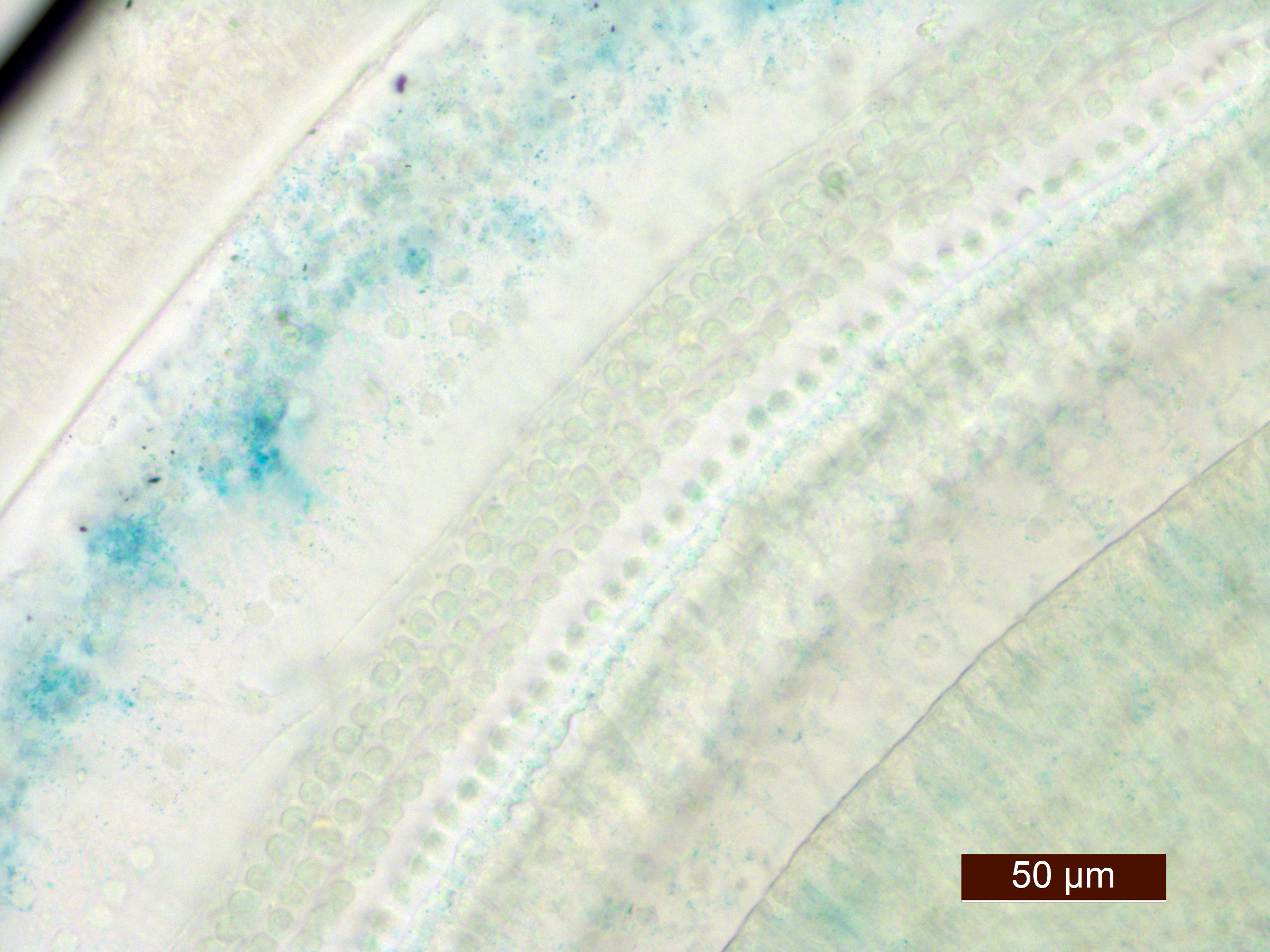

Supplement: Supplemental Information 4 — Raw data for ABR thresholds, protein expression, SA- β-gal positive cells [file peerj-10-14267-s004.zip › figure1/SA-a┬-gal-Figure1/Middle.png]

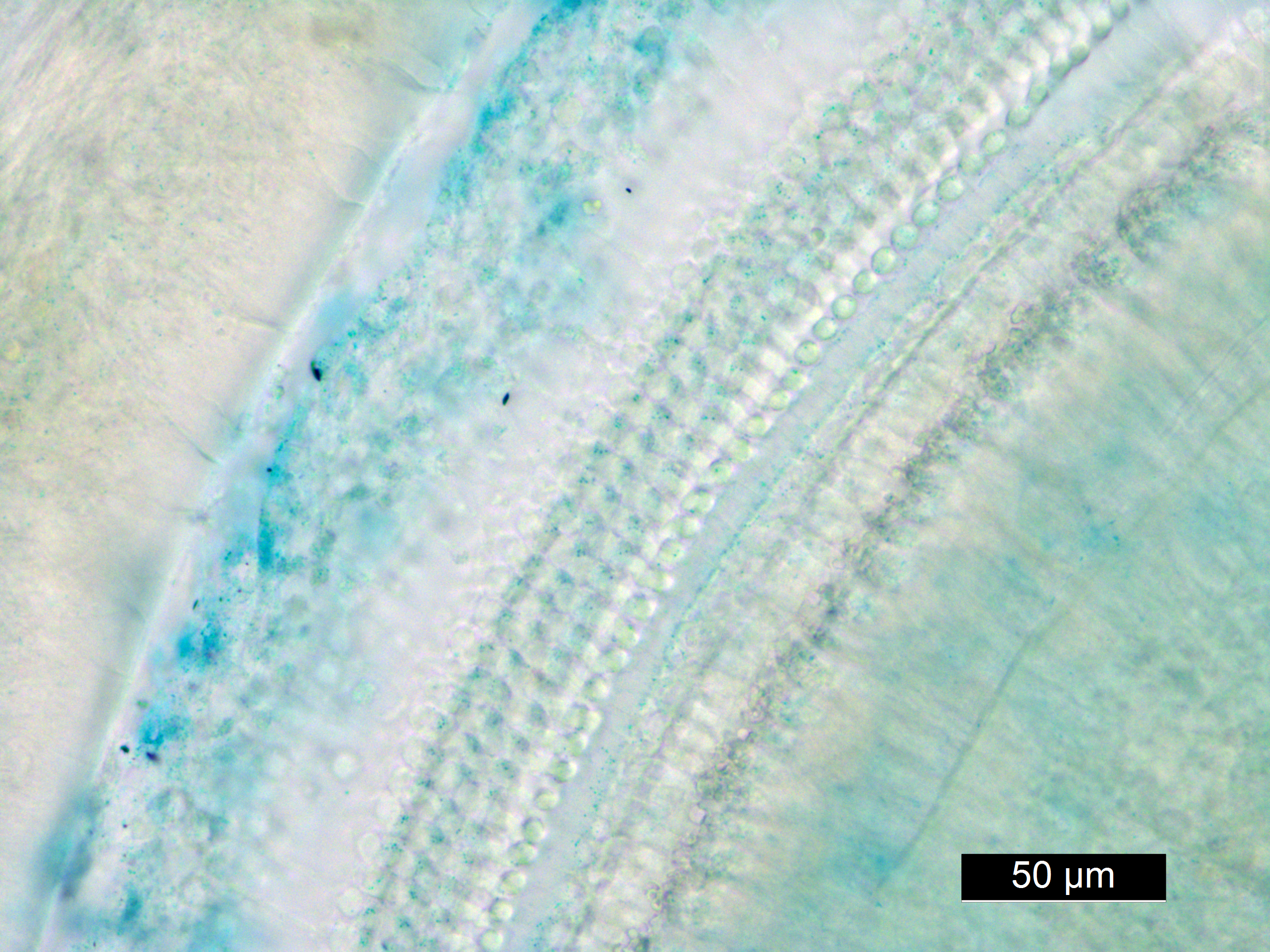

Supplement: Supplemental Information 4 — Raw data for ABR thresholds, protein expression, SA- β-gal positive cells [file peerj-10-14267-s004.zip › figure1/SA-a┬-gal-Figure1/Old.png]

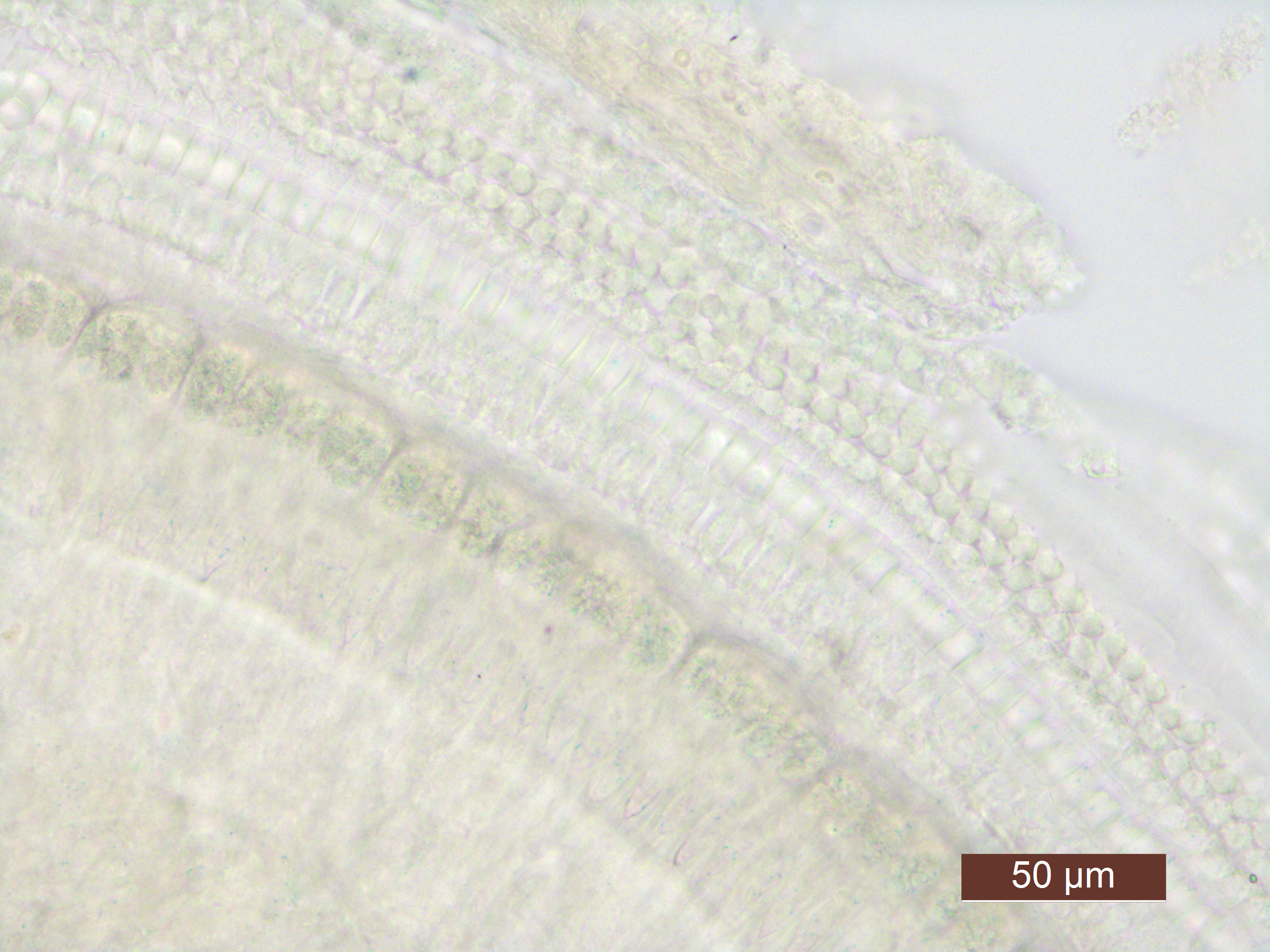

Supplement: Supplemental Information 4 — Raw data for ABR thresholds, protein expression, SA- β-gal positive cells [file peerj-10-14267-s004.zip › figure1/SA-a┬-gal-Figure1/Young.png]
